# Nd(III) and Gd(III) Sorption on Mesoporous Amine-Functionalized Polymer/SiO_2_ Composite

**DOI:** 10.3390/molecules26041049

**Published:** 2021-02-17

**Authors:** Khalid A. M. Salih, Mohammed F. Hamza, Hamed Mira, Yuezhou Wei, Feng Gao, Ayman M. Atta, Toyohisa Fujita, Eric Guibal

**Affiliations:** 1 Guangxi Key Laboratory of Processing for Non-Ferrous Metals and Featured Materials, School of Resources, Environment and Materials, Guangxi University, Nanning 530004, China; Immortaltiger7@gmail.com (K.A.M.S.); gaofeng@gxu.edu.cn (F.G.); fujitatoyohisa@gxu.edu.cn (T.F.); 2 Nuclear Materials Authority, P.O. Box 530, El-Maadi, Cairo 11381, Egypt; hamedmira@yahoo.com; 3 School of Nuclear Science and Technology, Shanghai Jiao Tong University, Shanghai 200240, China; 4 Chemistry Department, College of Science, King Saud University, P.O. Box 2455, Riyadh 11451, Saudi Arabia; 5 Polymers Composites and Hybrids (PCH), IMT Mines Ales, CEDEX, F-30319 Alès, France

**Keywords:** functionalized mesoporous silica, rare-earth elements, sorption isotherms, uptake kinetics, sorbent recycling, ore leachate

## Abstract

The strong demand for rare-earth elements (REEs) is driven by their wide use in high-tech devices. New processes have to be developed for valorizing low-grade ores or alternative metal sources (such as wastes and spent materials). The present work contributed to the development of new sorbents for the recovery of rare earth ions from aqueous solutions. Functionalized mesoporous silica composite was synthesized by grafting diethylenetriamine onto composite support. The physical and chemical properties of the new sorbent are characterized using BET, TGA, elemental analysis, titration, FTIR, and XPS spectroscopies to identify the reactive groups (amine groups: 3.25 mmol N g^−1^ and 3.41 by EA and titration, respectively) and their mode of interaction with Nd(III) and Gd(III). The sorption capacity at the optimum pH (i.e., 4) reaches 0.9 mmol Nd g^−1^ and 1 mmol Gd g^−1^. Uptake kinetics are modeled by the pseudo-first-order rate equation (equilibrium time: 30–40 min). At pH close to 4–5, the sorbent shows high selectivity for rare-earth elements against alkali-earth elements. This selectivity is confirmed by the efficient recovery of REEs from acidic leachates of gibbsite ore. After elution (using 0.5 M HCl solutions), selective precipitation (using oxalate solutions), and calcination, pure rare earth oxides were obtained. The sorbent shows promising perspective due to its high and fast sorption properties for REEs, good recycling, and high selectivity.

## 1. Introduction

The world demand for rare earth elements (REEs) is driven by the tremendous development of high-technical applications [1]. For example, neodymium and gadolinium have been used for manufacturing permanent magnets, motors, cry-coolers, lasers, high-tech glasses (for Nd), and for neutron therapy, magnetic resonance imaging, positron emission tomography, shielding in nuclear reactors, electronics, and electrolyte in solid oxide fuel cells (for Gd) [2]. The concentration of production facilities in a limited number of countries made the control of REEs supply a geostrategic issue [2]. Many incentive politics have been promoted in countries for developing the recovery of REEs from spent industrial materials [3,4,5,6], unconventional resources such as red mud by-products [7,8], or phosphoric acid and phosphogypsum processing [9,10,11,12]. Leaching remains the most frequently technique used for the processing of ores or waste materials [7]. Several alternatives exist for the recovery of REEs from leachates including solvent extraction [13,14,15] and impregnated resin [16]. However, most of the studies focus on sorption processes involving ion-exchange [17] or chelating resins [18,19,20], graphene-based sorbents, [21] carbon [22], clays, [23] functionalized silica [24,25], extractant impregnated layered double hydroxide [26], and functionalized bioadsorbent polymers [27,28,29]. Crystalline metal-organic frameworks (MOFs) derived from amino acid [30] camphorate MOFs with either acetate or formate as the auxiliary ligand [31]. Using silica-based materials offers many advantages, such as high mechanical strength and large versatility for designing size-controlled micro-particles with high specific surface areas (SSA). However, their sorption properties are generally relatively weak and poorly selective for metal ions. Immobilizing reactive thin layers allows enhancing their potential for metal recovery and maintaining appreciable SSA. Coating silica beads with glycidyl methacrylate is opening the possibility to graft additional and selective reactive groups. This is the strategy that was selected in the current study for synthesizing a functionalized composite sorbent (NH_2_/SiO_2_), which is applied for the sorption of two REEs—Nd(III) and Gd(III). Ethylenediaminetetracetic acid (EDTA) was used for the functionalization of mesoporous silica to improve Nd(III) sorption [25]. The polymer coating is processed by polymerization reaction between glycidyl methacrylate and *N*,*N*’-methylenebisacrylamide (as crosslinking agent) with azobisisobutyronitrile (AIBN) as the initiator and diluents additive. Polyamino ligands are well known as adsorbents for their affinity to bind dyes, and metal ions including REEs [32,33,34,35]. Inspired by these works, diethylenetriamine (DETA) was reacted with polymer-coated silica particles to produce the amino-rich silica sorbent. The main objectives for designing this novel material is to increase the chemical and mechanical stability of the composite by using silica core by choice of monomers, crosslinking agent, the diluent used for the polymerization, and additional reactive groups. The material is deeply characterized using a wide diversity of analytical tools (BET, SEM-EDX, TGA, FTIR, XPS, elemental analysis, and titration) for understanding the textural and structural properties, the chemical modification and composition of the sorbent, and for clarifying the binding mechanisms between reactive groups and target metal ions. The sorption properties of the prepared adsorbents are tested in a batch reactor for evaluating the pH effect, uptake kinetics, sorption isotherms, selectivity issues, metal desorption, and sorbent recycling. The application of the sorbent for the selective recovery of REEs was investigated by treatment of the acidic leachate of Gibbsite ore materials (polymetallic ore collected from mining sites). This study offers a very complete overview of the design of a new composite organic/functionalized polymer sorbent, with extensive characterization of the material and its interactions with two specimens of light rare earth element (i.e., Nd(III)), and heavy rare earth element (i.e., Gd(III)). This study demonstrates the remarkably high sorption capacities of the designed sorbent for REEs. The wide study of the sorption properties of these REEs through a progressive complexity of the metal-containing solutions (synthetic simple versus synthetic multi-component solutions and finally real ore leachates) is also offering a unique overview of the facilities and limits of this sorbent for valorizing strategic metals. In the case of the real acidic leachate, the study is driven up to the production of highly pure concentrate of REE oxides.

## 2. Results and Discussion

### 2.1. Characterization of Sorbent

**Figure 1:**
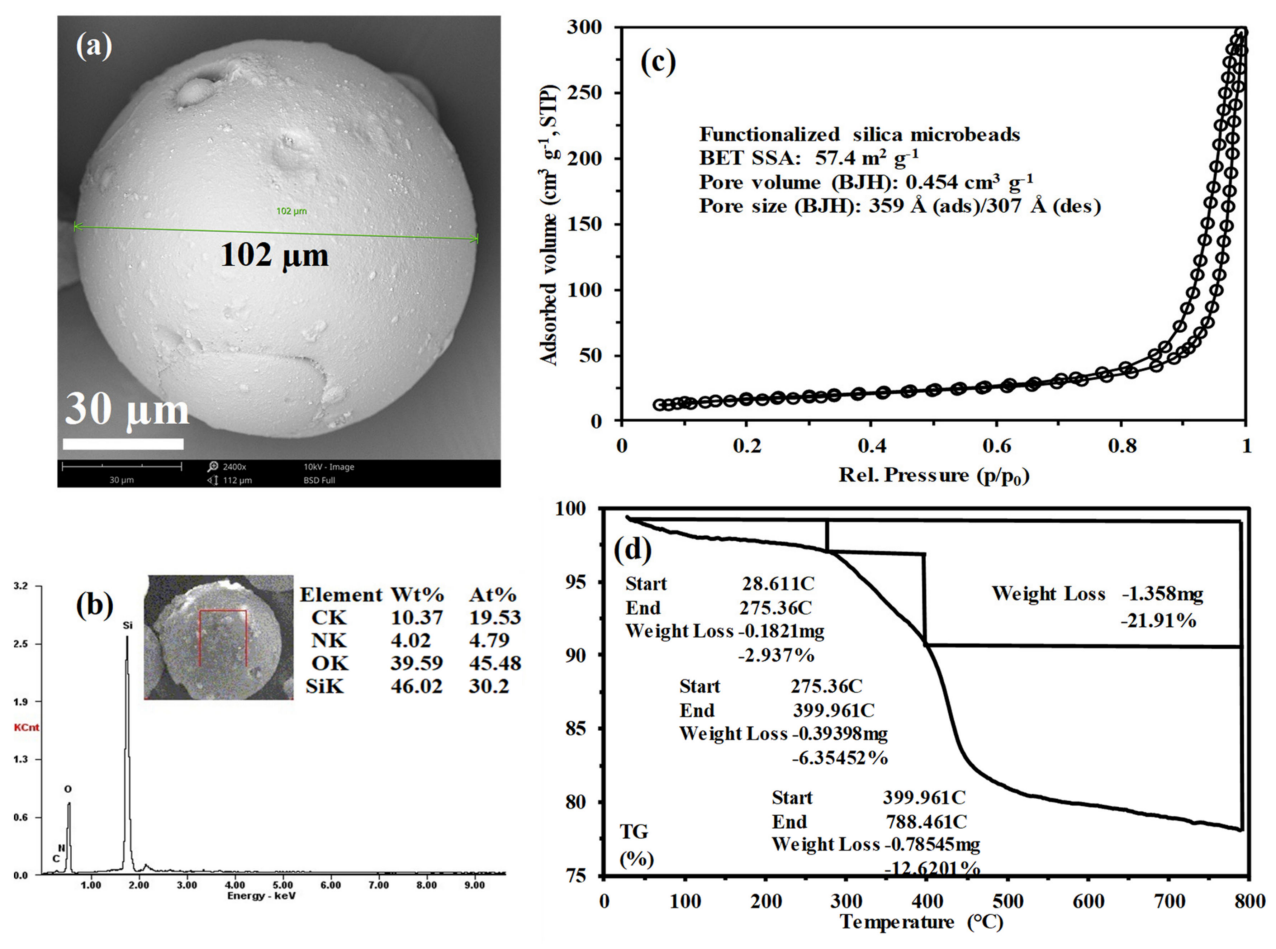
Characterization of functionalized sorbent: (**a**) SEM micrograph, (**b**) semi-quantitative EDX analysis of sorbent surface, (**c**) BET analysis of silica beads and silica microbeads (sorbent), and (**d**) TGA analysis of sorbent.

Figure 1 shows some of the physical characteristics of the sorbent—morphology of sorbent particles by SEM micrographs (Figure 1a), surface composition by semi-quantitative EDX analysis (Figure 1b), specific surface area by BET measurements (Figure 1c), and TGA measurements (Figure 1d). More detailed (and/or comparative with silica beads) presentations are reported in the Supplementary Materials Section. Table S1 compares the shape and size of SiO_2_ and composite functionalized sorbent (SEM micrographs of small/large particles), which was still characterized by spherical shape with a small increase in the size of the particles—from 85–100 µm to 100–115 µm. This observation confirms that the effective functionalization of raw material and a thin layer (about 15 µm) of polymer is deposited at the surface of silica particles. The small thickness of this layer suggests that the contribution of the resistance to diffusion of sorbate molecules through the polymer will have a limited impact on sorption kinetics.

Figure S1 reports the textural analysis of composite functionalized sorbent (and its precursor, raw SiO_2_ micro-beads). The specific surface area (SSA) is close to 69.1 m^2^ g^−1^ for SiO_2_ microbeads, and their functionalization decreases the SSA to 57.4 m^2^ g^−1^. The functionalization of mesoporous silica by grafting organic ligand reduces BET area and pore volume [36]. The grafting of polymers at the surface of porous silica materials usually decreases the SSA and porous characteristics of sorbents [37]. Actually, the functionalization of silica micro-beads only reduces by 18% their SSA. The shape of the isotherms is similar for the two materials; they can be roughly assimilated to type IV isotherm. The hysteresis loop, which is materialized by the non-superimposition of adsorption and desorption isotherms, corresponds to type H_2_ [38]; however, the width of the loop is weakly marked compared to other composite silica materials [39]. H2 loop is usually associated with the so-called ink bottle-neck porous system—this is even more marked for polymer functionalized material due to the particle obstruction of micropores at the surface of raw silica microbeads. This is confirmed by the comparison of SSA due to micropores that were found close to 7.6 m^2^ g^−1^ for raw SiO_2_ (corresponding pore volume: 0.0035 cm^3^ g^−1^); after functionalization, the microporous volume becomes negligible and the relevant SSA is limited to 0.55 m^2^ g^−1^. In addition, the loop is detected in the relative pressure range (p/p_0_) 0.7–0.99 for functionalized silica microbeads against 0.8–0.99 for raw silica microbeads. The distribution of pore size is shifted toward lower pore size after functionalization of silica microbeads; as consequence, the smallest pores are filled and remain only the largest pores (mesoporous) and the final average pore sizes (for both adsorption and desorption) tends to increase.

The mesoporous composite functionalized sorbent shows a specific surface one order of magnitude lower than the SSA of fibrous mesoporous silica microspheres and amine-functionalized microporous and mesoporous silica [40]. However, the porous volume is maintained at a relatively high value (i.e., 0.454 m^2^ g^−1^) for a material having large mesopores (average pore size is in the range 307–359 Å through N_2_ desorption and adsorption methods, respectively).

**Scheme 1:**
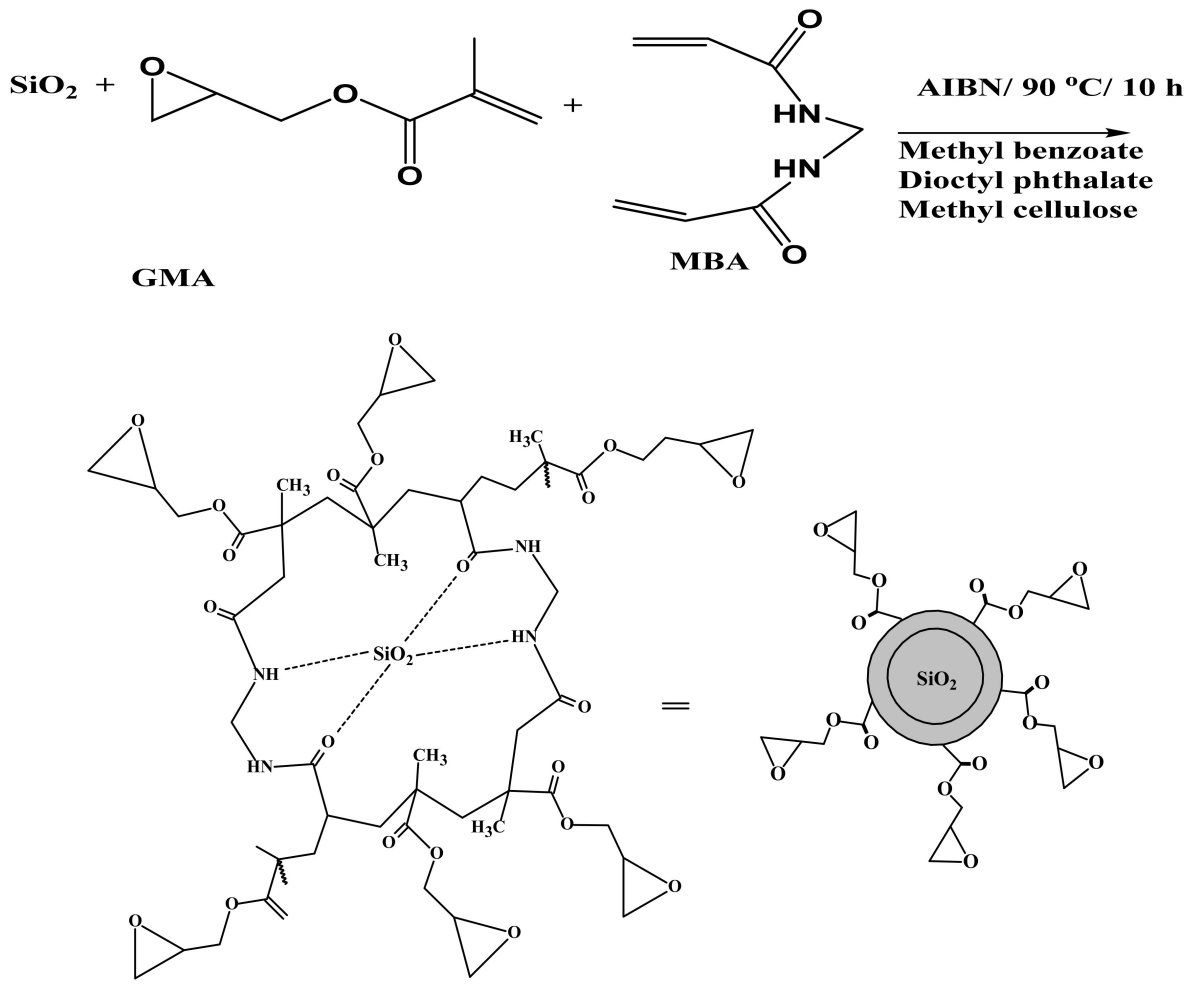

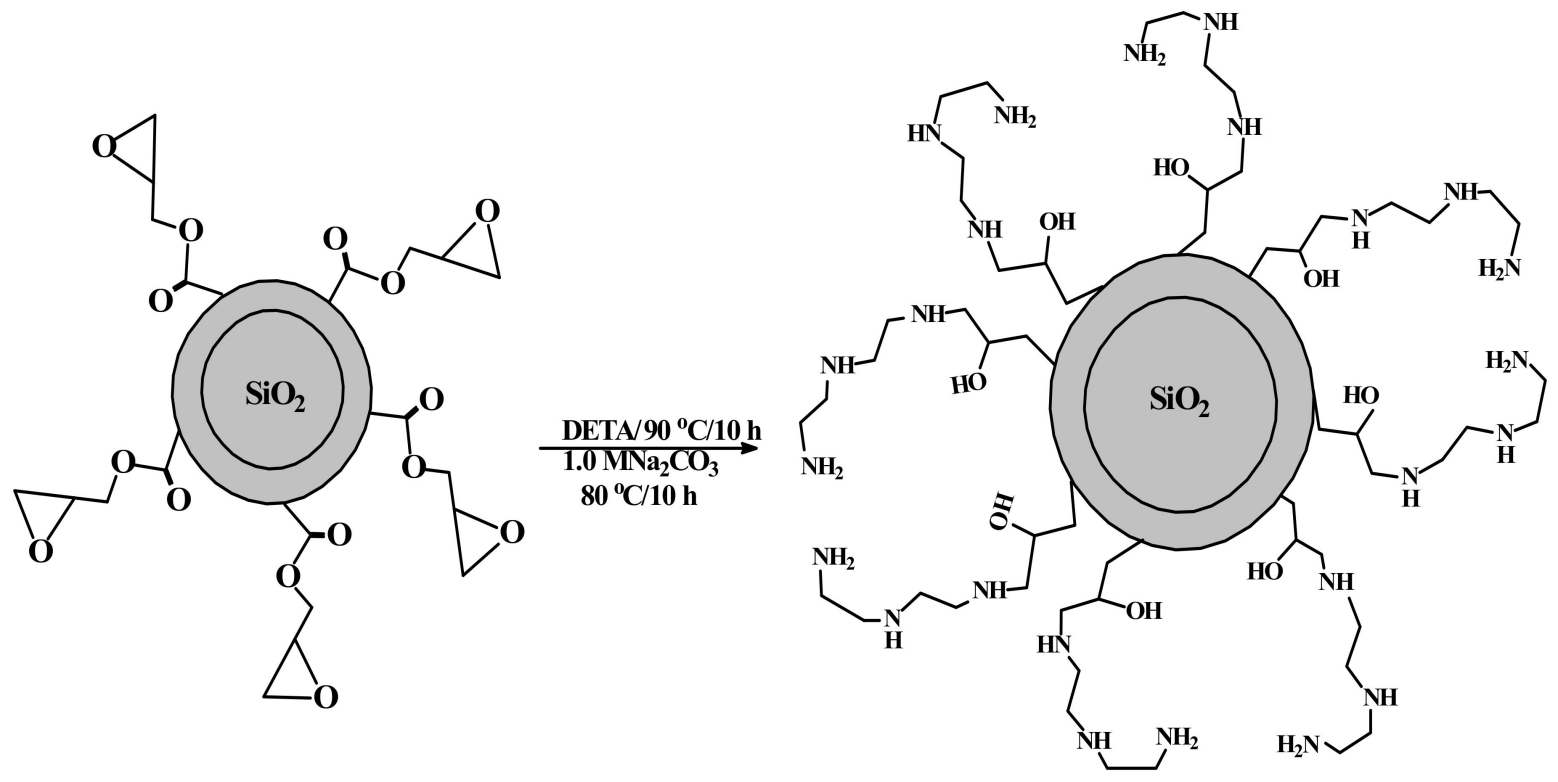
Synthesis pathway for mesoporous composite functionalized sorbent.

Table S2 compares the SEM micrographs and the EDX semi-quantitative analysis of the surface for silica beads. The substantial increase of C content (from 2.29% to 19.53% atomic percentage (AP)) and the appearance of N element (up to 4.79% AP) in the mesoporous composite functionalized sorbent confirms the effective grafting of polymer on the inorganic support. The elemental analysis shows that the relative percentages of C, H, and N elements are 22.96%, 5.02%, and 4.55% (*w*/*w*), respectively. The nitrogen content is consistent with semi-quantitative EDX analysis (i.e., 4.79%). This corresponds to a molar concentration of N close to 3.25 mmol N g^−1^. Taking into account the suggested structure of the composite (Scheme 1), this means that the sorbent bears 1.08 mmol –NH_2_ g^−1^ (primary amino groups, ending groups) and 2.17 mmol NH g^−1^ (secondary amino groups). The reactivity of these different amine groups is affected by their chemical environment (acid/base properties, inductive effect) and hindrance effect [41,42]. This may influence, in turn, the mechanisms that contribute to metal binding. Free amine groups are preferred for chelation, while the protonation of secondary amine groups may be useful for binding metal ions through ion-exchange/electrostatic attraction mechanism [43]. The glycidyl methacrylic grafting allows efficient insertion of amino groups on silica microbeads. Quantitative substitution of amines was calculated by volumetric titration. According to this, the content of amines in the synthesized sorbent close to 3.41 mmol N g^−1^, which is slightly higher than the nitrogen values that were reported by the elemental analysis by 0.16 mmol N g^−1^ (i.e., 3.25 mmol N g^−1^ (based on elemental analysis)). This indicates that the successfulness grafting of the amine moieties on the silica base materials. The variation is acceptable and may due to some amine groups in the sorbent bores are hardly detected by the EA tool than the titration process, (especially for the sufficient time reaction). Thermogravimetric analysis (Figure S2) shows different steps in the thermal degradation. A first step (occurring between 28.6 °C and about 150 °C) represents a weight loss of 2.2%, which is associated with the release of surface-absorbed water and internal water bound to silica or polymer coating. This is associated with a poorly resolved shoulder in the DSC profile close to 100 °C. Another strong DSC peak is detected at 275 °C. The second step in the thermal degradation counts for a weight loss close to 8.8% (i.e., total weight loss close to 11%) and takes place in the temperature range 150–370 °C. This weight loss is associated with the degradation of the amine-based polymer and/or the glycidyl methacrylic acid polymer fraction [44]. Another step is identified in the range 370–530 °C with an additional weight loss of 5%; total weight loss is close to 16% (which is consistent with the loss reported for amine-grafted silica composites). Above 530 °C, another weak and progressive degradation step is observed, which is associated with the final degradation of the polymer residues and the desorption of water released during silanol condensation [45]. This additional weight loss corresponds to 6% at 787 °C (total weight loss: 21.91%). This means that polymer coating represents about 21–22% of the total weight of the composite functionalized sorbent. It is noteworthy that this fraction is consistent with the relative proportions of inorganic and organic precursors (i.e., 4:1, respectively).

**Figure 2:**
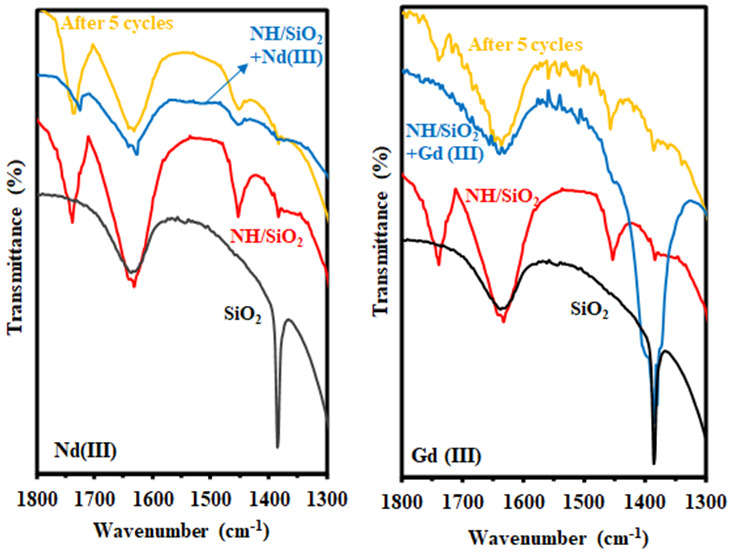
Selected wavenumber range (1800–1300 cm^−1^) for FTIR spectra of SiO_2_, functionalized sorbent (NH/SiO_2_), sorbent after Nd(III) and Gd(III), and after five sorption and desorption cycles.

Figures S3 and S4 show the FTIR spectra of mesoporous SiO_2_ and the composite functionalized sorbent (NH/SiO_2_), respectively. The main peaks on the SiO_2_ spectrum are identified at 3450 cm^−1^ (–OH stretching vibrations), 1650 cm^−1^ (associated with –OH groups due to residual adsorbed water [45], and Si–OH bending vibration). The bands at 1160 cm^−1^, 472 cm^−1^, and 802 cm^−1^ are assigned to Si–O–Si asymmetric stretching, bending or rocking mode, and Si–O symmetric stretching vibrations, respectively [45]. The peak at 472 cm^−1^ is also attributed to Si–O–Si bond (bending or rocking mode) [46]. The peak at 1378 cm^−1^ demonstrates the present CH_3_ groups (bending vibration). After silica functionalization, new bands appear at 2934 cm^−1^ and 2875 cm^−1^ (C–H stretching vibrations), and 1454 cm^−1^ (–CH stretching vibration). The peak at 1738 cm^−1^ is assigned to the C=O bond in ester groups [47]; this is directly correlated to the effective grafting of methacrylate moieties (Figure 2). The increase in the intensity of the peak at 3450 cm^−1^ can be explained by the successful immobilization of amine on glycidyl moieties. The large band between 1080 cm^−1^ and 1220 cm^−1^ on raw SiO_2_ is modified after functionalization with the appearance of a resolved peak at 1105 cm^−1^ and a shoulder at around 1213 cm^−1^. More details on the assignments of the peaks are reported in Table S3.

**Figure 3:**
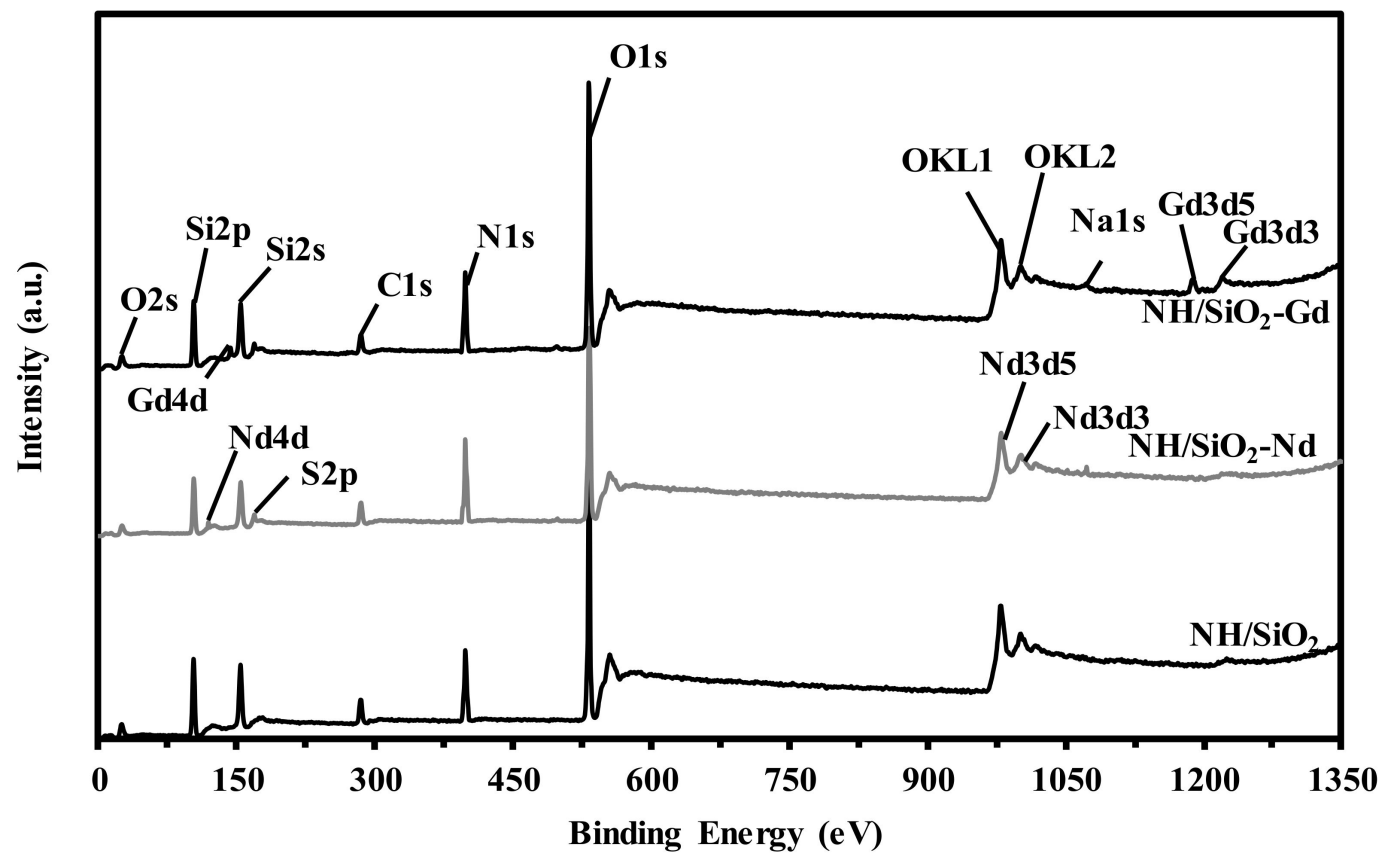
XPS spectra (survey) of silica functionalized sorbent (NH/SiO_2_) before and after sorption of Nd(III) and Gd(III) ions.

Figure 3 shows the XPS survey spectrum of mesoporous composite functionalized sorbent (NH/SiO_2_). The peaks representative of Si element (Si *^2^*p and Si *^2^*s at binding energies (BEs) 103 eV and 154 eV, respectively) are clearly identified (associated with O *^1^*s at BE 534 eV, O KL1, and O KL2 peaks). The functionalization of the support is marked by the presence of the signal assigned to N *^1^*s (BE: 398 eV). Tables S4 and S5 show the high-resolution XPS spectra of selected signals and the assignment of their relevant BEs. The Si *^2^*p signal is represented by two peaks corresponding to Si *^2^*p3/2 and Si *^2^*p1/2 at BEs 102.94 eV and 104.5 eV, respectively [48]. The C *^1^*s signal for composite functionalized sorbent is deconvoluted into two peaks at BEs 283.75 eV and 284.72 eV, corresponding to adventitious C (and C–H) [49] and to C–O, C=O, C–N, or –O–C environments, respectively. The O *^1^*s signal splits into three peaks at BEs 532.35 eV, 533.95 eV, and 535.3 eV, which may be assigned to O (C, H, =C), O–Si–O, and C–O–C, respectively [50]. The new peaks associated with organic fraction confirm the effective grafting of glycidyl methacrylate. Their environment is affected by the amination, which is demonstrated by the interpretation of the N *^1^*s signal (Figure 3). The deconvolution of N *^1^*s into two peaks at BEs 398.36 eV and 400 eV, which are attributed to N (C, =C, H) and O=C–NH– environments, respectively [51]. FTIR and XPS analyses confirm the accuracy of Scheme 1 for describing the functionalization of silica particles. Silica particles contain the siloxy groups on the surface, which are bound with the reactive functional groups that are assisted by the high temperature; this is performed by an either chemically or physically binding process. The chemically bonding type was performed through the hydroxyl groups and either the epoxy groups from the monomers or with amine moieties from the MBA. On the other hand, physical attraction can be performed by the hydrogen bonds with hydroxyl groups versus the polarized groups (i.e., amines and carbonyls), as reported in Scheme 1. This is emphasized by the presence of the Si ions in the EDX analysis after the sorption desorption cycles (see FTIR and EDX analysis).

Figure S5 shows the pH-drift method application for the determination of pH_PZC_. The value slightly hardly varies with the concentration of the background salt in the range 6.20–6.33. The pH_PZC_ can be evaluated at 6.27. This means that the sorbent is protonated in acidic conditions. This value is consistent with the values reported for the isoelectric point of 3-aminopropyl triethoxysilane-functionalized silica (i.e., 7).

### 2.2. Sorption Properties

#### 2.2.1. pH effect

**Figure 4:**
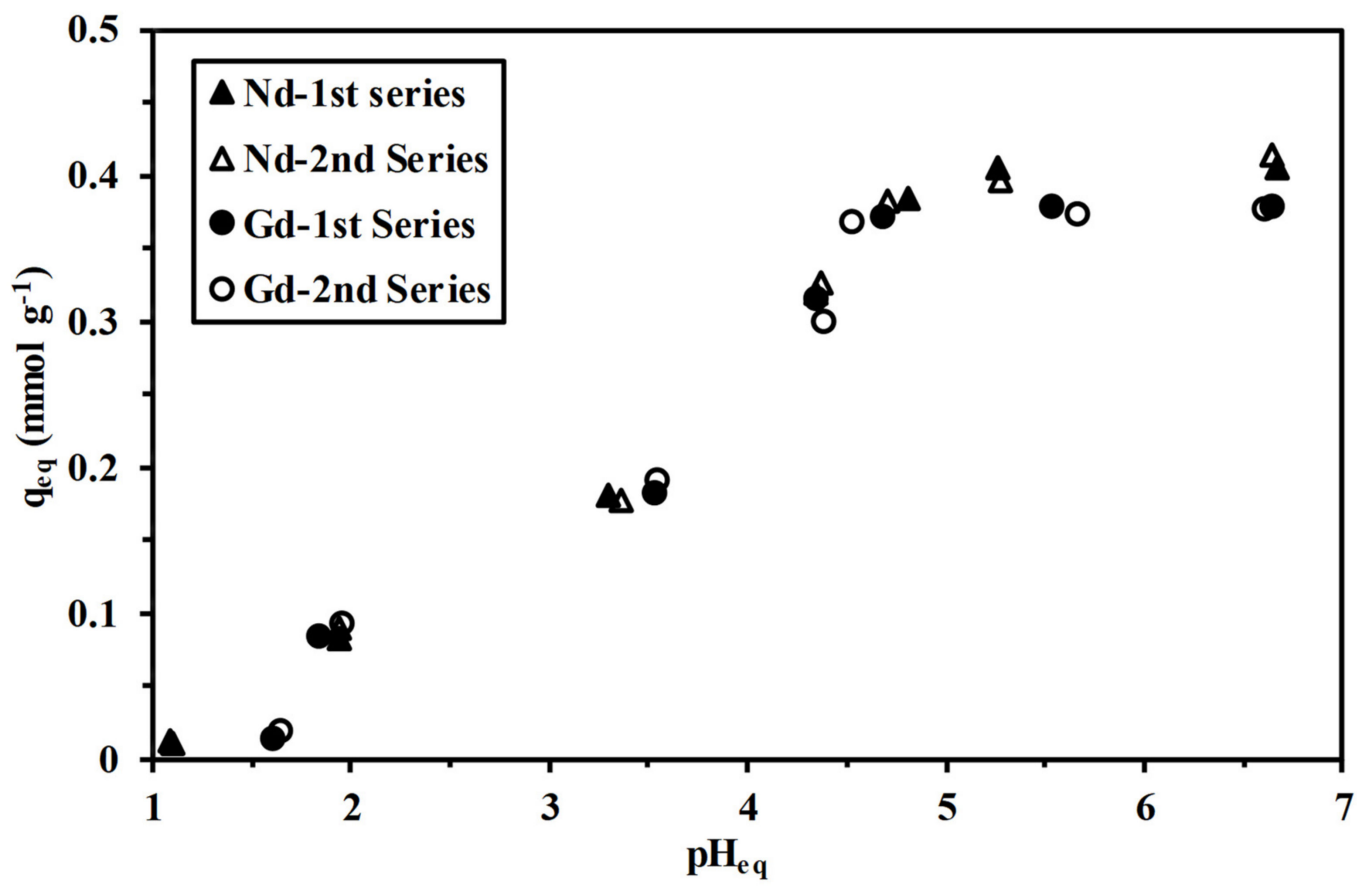
Effect of equilibrium pH on Nd(III) and Gd(III) sorption capacities using mesoporous composite functionalized sorbent (C_0_: 0.63–0.66 mmol metal L^−1^; sorbent dosage (SD): 1.42 g L^−1^; contact time: 48 h; agitation speed: 170 rpm; T: 22 ± 2 °C).

Figure 4 shows the effect of equilibrium pH on the sorption of both Nd(III) and Gd(III). The superimposition of the curves for the duplicates shows the good reproducibility of sorption performance. In addition, the superimposition of the curves for Nd(III) and Gd(III) binding confirm that the two REEs have a very similar sorption behavior for composite functionalized sorbent (NH_2_/SiO_2_). It is already possible to anticipate that the separation of the two metal ions with the sorbent will be difficult. It was also noticed that there is negligible adsorption below pH 1.5 that referred to protonation of amine groups of the adsorbent. Moreover, the H^+^ will compete with Nd(III) and Gd(III) to adsorb on the active site of the adsorbent. The sorption capacity linearly increases with pH from pH 1.5 to 5 before stabilizing (up to pH 6.5). With the pH_PZC_ being close to 6.2, the sorbent is protonated in acidic conditions. Figure S6, a shows the speciation diagrams for Nd(III) and Gd(III) under the experimental conditions for the study of the pH effect. Cationic metal species largely predominate in the whole pH range—above pH 3, Nd(III) and Gd(III) are both exclusively present as free REE^3+^ or REESO_4_ cationic species; between pH 1 and pH 3, the fraction of anionic species (REE(SO_4_)^2−^) decreases from 17% to 0%. In very acidic conditions, the competition of counter anions (i.e., sulfate anions) inhibits the sorption of anionic species on protonate amine groups. With the pH increases, the concentration of sulfate anions decreases, and the sorbent can bind anionic REEs species (present at low concentration) until pH 2.5. With the increase of the pH, the sorbent progressively deprotonates, making more efficient the sorption of REEs on the composite functionalized sorbent.

The XPS analysis of sorbent after metal sorption shows that sulfate is present on the sorbent; this may be due to direct binding of anions and through the binding of REE− sulfate species. It is noteworthy that Gd(III) begins to precipitate under selected experimental at pH higher than 6.25; the saturation plateau above pH 6 is thus not meaningful. A similar pH-edge was reported for the sorption of Nd(III) using EDTA and DTPA-chitosan/SiO_2_ sorbents, with DETA-functionalized magnetic chitosan and with amino-modified mesoporous sorbent [52]. Literature survey shows that the optimal pH range around 4–5 is consistent with previous studies; the sorption performances are compared below (with relevant compilations of data on tables in supplementary file). Figure S7 shows the log_10_ plot of the distribution ratio, D (q_eq_/C_eq_, L g^−1^) versus pH_eq_. The slope of the curve, in the pH range 2–5.7 is close to 0.5 (i.e., 0.48 for Nd(III) and 0.45 for Gd(III)). For ion-exchange processes, this is usually associated with the stoichiometry of proton exchange between sorbed species and reactive groups on the sorbent. The stoichiometric ratio is thus close to 2 –NH^3+^/–NH^2+^ for 1 REE species. Figure S8 summarizes the pH variation during Nd(III) and Gd(III) sorption using composite functionalized sorbent. The pH variation was, in most cases, less than 0.5 pH units. The pH tends to increases for pH below 4.5, due to sorbent protonation, while above pH 4.5 the solutions tend to be acidified probably due to proton exchange with REEs cations. It is noteworthy that pH variation during metal sorption is of the same order of magnitude as the ΔpH measured for the determination of pH_PZC_ (Figure S5).

#### 2.2.2. Characterization of Sorbent Interactions with Metal Ions

On FTIR spectra, the shifts and the decrease in the intensities of the bands associated with amine (and OH stretching, and amide groups) on metal-loaded sorbents confirm the contribution of these reactive groups in metal binding (or at least the modification of their chemical environment). On the other hand, the spectra of the sorbents after five cycles of sorption and desorption can be compared to the spectrum of the virgin sorbent (Figure 3). The spectra are very close—the material shows remarkable stability toward acidic conditions and reversal sorption.

**Figure 5:**
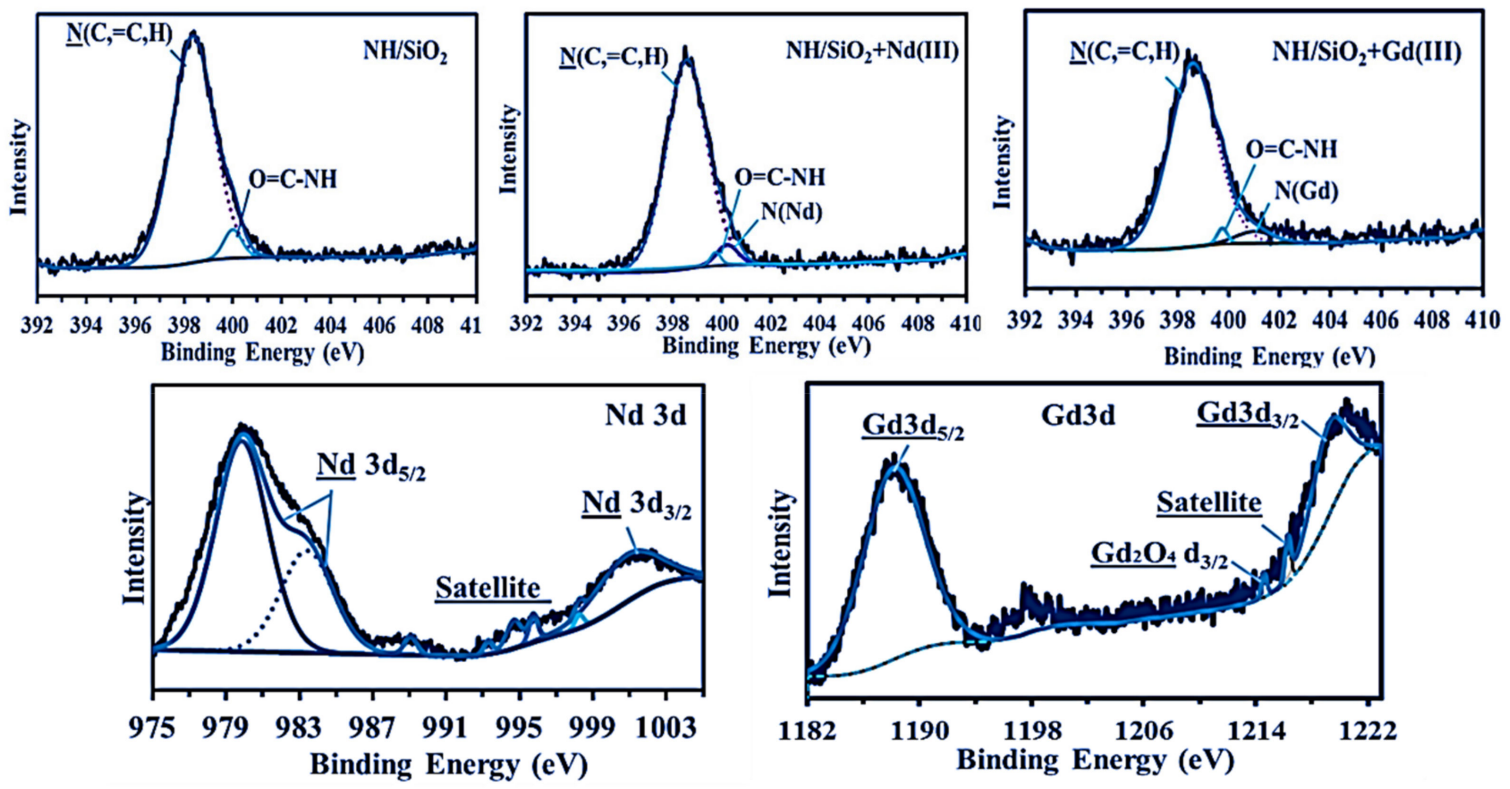
High-resolution (HRES) XPS spectra of N *^1^s* for either loaded and non-loaded sorbent and Nd or Gd *^3^*d species.

The sorption of Nd(III) and Gd(III) on mesoporous composite functionalized sorbent affects, the environment of C *^1^*s, O *^1^*s, and N *^1^*s signals. Tables S7 and S8 identify the change in the relative atomic fractions, the appearance of new peaks, or the shift in BEs associated with metal binding. After Nd(III) sorption, the signal C *^1^*s is split into three peaks at BEs; 284.48 eV, 285.82 eV, and 287.01 eV corresponding to C (C, H, N), C(–O, =N) [53], and (C–O–C, N–C=O, O–C=O) [54], respectively. After Gd(III) sorption, the band associated with C=O in the ester group (O–C=O and amide N–C=O) appears at higher BEs 289.55 eV and 287.8 eV, respectively. Other peaks corresponding to C (C, H, N), and C(–O, =N, O–C) are weakly shifted at BEs 284.56 eV and 286.25 eV, respectively. The O *^1^*s spectra show a new peak (M–O) after metal sorption at BE 529.3 eV, independently of the metal binding [55]. Other O *^1^*s peaks are shift to higher BEs level after Nd(III) binding, i.e., 532.47 eV, 534.25 eV, and 535.75 eV for O(C, H,=C) SiO_2_, and C–O–C, respectively. For Gd(III) sorption, O(C, H,=C) and SiO_2_ are identified at BE 532.43 eV [56], while the signal at BE 534.3 eV which assigned to O–S of sulfate groups [57] and to C–O–C. This means that metal binding is partly occurring through oxygen sharing. This is consistent with the decrease of the intensity of the –OH and C=O groups observed with FTIR spectrometry. At high pH, the tautomerization effect contributes to the enhancement of metal binding. The sorption of Nd(III) and Gd(III) hardly affects the BEs of the signals identified on the sorbent; however, new peaks appear at higher BEs (than reference peaks) at 400.65 eV for Nd(III) and 401 eV for Gd(III), corresponding to protonated amine groups (Figure 5) [58]. This observation confirms that positively charged complexes are bound onto *N*–bearing reactive groups (lone pair of electrons). The signals associated with Si *^2^*p are not significantly affected by metal sorption; this confirms that the binding of REEs proceeds exclusively through the reactive groups immobilized on the polymer coating.

After metal sorption, the signal S *^2^*p appears; this is directly correlated to the binding of metal ions through sulfate complexes (or to direct sorption of sulfate anions released from salt and acid dissociation). In the case of Nd(III), the signal splits into two peaks at BEs 165.39 eV and 166.5 eV, which are related to S *^2^*p1/2 [59]. For Gd(III) binding, the S *^2^*p signal is deconvoluted into six peaks at BEs 165.65 eV and 167 eV for S *^2^*p1/2 [59], while BE 164.1 eV is related to S *^2^*p3/2 [59], and BEs 168 eV, 167.85 eV, and 169 eV for SO_4_^2−^.

After Nd(III) sorption, Nd *^3^*d signal is deconvoluted into three main peaks corresponding to Nd *^3^*d5/2 at BEs 980.16 eV and 984.05 eV, and Nd *^3^*d3/2 at BE 1000.85 eV, while the satellite peaks appear at BEs 994.7 eV, 995.75 eV, 989.05 eV, 993.3 eV, and 998.25 eV with low AF% (around 4.49%) (Figure 5). These data confirm that neodymium is bound under its trivalent oxidation state; large FWHMs are associated with (a) the exchange interaction mode of ^3^d hole and ^4^f (partially filled) of Ln^3+^ (with Ln: Nd and Gd) [60] and (b) the oxidation state of Ln atoms [61]. Nd *^4^*d signal splits into two peaks at BEs 117.74 eV and 118.4 eV, while Nd *^4^*s and Nd *^3^*p3 show two identical peaks at BEs 316.21 eV and 1301.07 eV, respectively. Gd *3*d signal can be deconvoluted into four peaks at BEs 1188.17 eV, 1214.6 eV, 1216.3 eV, and 1219.2 eV for Gd *^3^*d5/2, Gd_2_O_3_ *^3^*d3/2, satellite peak, and Gd *^3^*d3/2, respectively (Figure 5). These signals have been correlated with Gd in the trivalent oxidation state and more precisely the Gd_2_O_3_ form [62]. The Gd *^4^*d signal splits into three peaks at BEs 143.15 eV, 148.6 eV, and 151.15 eV for (Gd *^4^*d5/2, in Gd_2_O_3_), Gd *^4^*d3/2, and satellite peaks, respectively [63]. As a conclusion, the presence of these typical deconvoluted bands confirms the trivalent state of REEs bound on the sorbent, and the shapeup satellites support the hypothesis of the hybridization of Gd/Nd (*^3^*d and *^4^*d) with O *^2^*p and N *^2^*p.

**Figure 6:**
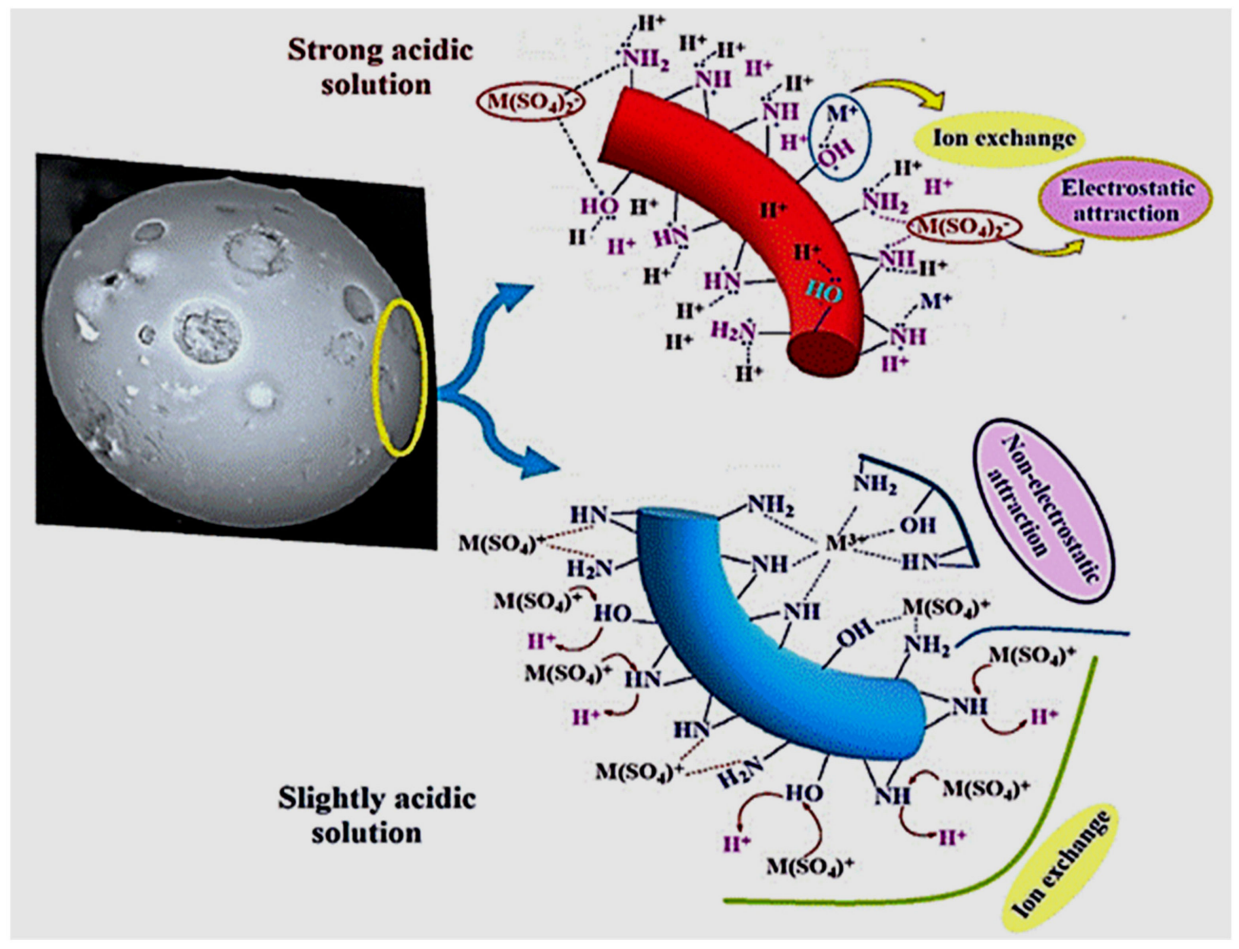
Tentative mechanisms for rare-earth elements (REEs) sorption on mesoporous NH/SiO_2_ sorbent.

Tables S6 and S7 compare the surface morphology of the sorbent and its semi-quantitative analysis before, after Nd(III) and Gd(III) sorption, and after metal elution. After metal sorption, the presence of the S element confirms that sulfate ions are bound on the sorbent (directly on protonated amine groups or as an REE–sulfate complex). The non-stoichiometric ratio between REEs and sulfate ions demonstrates that several species may be bound. After desorption, the metals are completely eluted (together with sulfate anions). Ramsamany et al. [24] reported the sorption of REEs on amino-functionalized mesoporous silica through an ion-exchange mechanism on hydroxyl groups at high pH (above 7), while amino groups are preferred at neutral or mid acidic pH for REEs binding. Different modes of interaction may be involved between the reactive groups and metal ions depending mainly on the pH of the solution (protonation/deprotonation of reactive groups and metal speciation). From XPS, FTIR analyses, and the slope of the log_10_ plot versus pH_eq_, it is possible to identify the ion-exchanger is the main sorption mechanisms through protons on reactive groups with positively charged metal ions. The pH_PZC_ being close to 6.27, which means that even at pH 5, the sorbent is not completely deprotonated making possible the proposed interaction; however, the reactive groups that are deprotonated may be marginally involved in chelation. At low pH values (fully protonated sorbent), positively charged REE ions (REE^+^ and REESO_4_^+^) that are mainly found in the solution at acidic pH bind through cation exchange mechanisms with protons found in the protonated amines and hydroxyls; however, the competition of protons and the electrostatic attraction of the anions (dissociated from the acid) leads to a decrease of REE binding. This effect is enhanced by the rather small ionic size of protons compared to rare-earth ions). In addition, a fraction of rare-earth elements that exist in the solution as anionic sulfate complexes (Figure S6) may bind through electrostatic attraction on protonated groups (see FTIR and XPS analysis that confirm the presence of sulfate and S groups, respectively). As pH increases, metal ions co-exist under the form of two positively charged species (REE^3+^ and REESO_4_^+^) (Figure S6), bind through the available electron pairs on either amines (primary, secondary, and tertiary) or hydroxyls by chelation. Figure 6 shows a tentative scheme representing the different mechanisms that can be involved in the sorption of REEs. It was reported that the two hydroxyl groups and one amino group of the amino-modified mesoporous silica (MCM-41) play an important role in the adsorption of divalent metal ions [64]. Moreover, the nitrogen containing groups will form complex with the metal ion by chelation, and their selectivity are based on the strength or stability constants for the bond formed between metal ions and the adsorbent surfaces. There are other mechanisms that will occur for adsorption of REE^3+^ and REESO_4_^+^, such as ion exchange, and electrostatic attractions that based on solution pH as illustrated in Figure 6 and supported by the effect of pH on their uptakes (Figure 4).

#### 2.2.3. Uptake Kinetics

**Figure 7:**
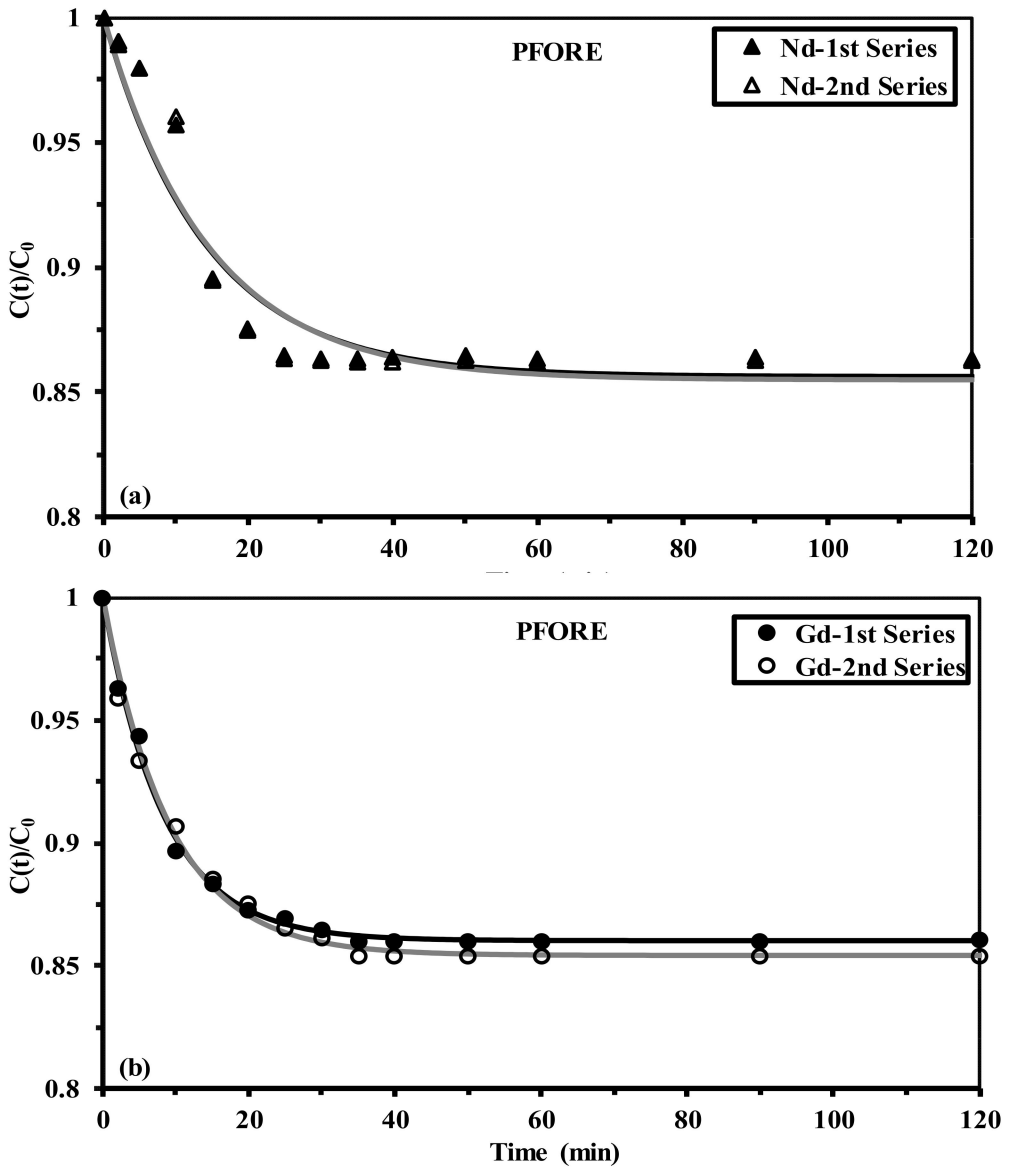
Nd(III) and Gd(III) uptake kinetics using mesoporous composite functionalized sorbent—modeling with the pseudo-first-order rate equation (C_0_: 0.65–0.71 mmol metal L^−1^; pH_0_: 5; pHeq: 4.69–4.76; SD: 0.25 g L^−1^; agitation speed: 170 rpm; T: 22 ± 2 °C).

Please note that experimental conditions (excess of metal compared with reactive groups through low sorbent dosage) have been selected to highlight the possible contribution of resistance to intraparticle diffusion; therefore, the residual concentration decrease by about 13%. An excess of sorbent would limit the sorption to external sites, minimizing the effect of resistances to mass transfer. The kinetic profiles show a good reproducibility in sorption performance (duplicated experiments) (Figure 7). In addition, the sorption is relatively fast under selected experimental conditions—25 min of contact is sufficient to reach the equilibrium for Nd(III) sorption, while 40 min are required for Gd(III). Despite this faster global kinetics for Nd(III), it is noteworthy that, the initial step of the uptake profiles (below 10 min of contact) show distinct trends—initial slope for Nd(III) sorption (i.e., 3.94–4.21 × 10^−3^ min^−1^) is weaker compared with that of Gd(III) uptake kinetics (i.e., 8.70–9.74 × 10^−3^ min^−1^). The differences in the ionic radius of hydrated species are not large enough to explain this difference in the initial sorption step (Table S8).

Figure 7 also shows the fits of experimental profiles with the PFORE for both Nd(III) and Gd(III), while the PSORE and RIDE fits are reported in Figures S9 and S10, respectively. The irregular shape of the initial part of the curve makes the modeling of Nd(III) uptake kinetics less accurate than for Gd(III). Table 1 summarizes the parameters of the models and the determination coefficients (i.e., R^2^). The superimposition of experimental data and simulated curves, the comparison of experimental and calculated q_eq_ values, and the values of R^2^ demonstrate that the PFORE fits the profiles preferentially to RIDE (and much better than PFORE). It is also confirmed by the values of the Akaike information criterion (AIC), which are, in most cases, much lower than the values obtained with PSORE and RIDE fits. The PFORE is usually associated with physical sorption (compared to PSORE, which is correlated to chemical sorption). The comparison of apparent rate coefficients k1 is higher for Gd(III) (i.e., 12.1–11.0 × 10^−2^ min^−1^) than for Nd(III) (i.e., 7.11–6.91 × 10^−2^ min^−1^); this is contradictory with the comparison of equilibrium times. This unexpected conclusion is probably due to the unexpected initial sorption that affects the global modeling of the kinetic profiles. The evaluation of the diffusivity coefficient of Nd(III) (in the range 4.71–4.61 × 10^−11^ m^2^ min^−1^) and Gd(III) (7.18–6.49 × 10^−11^ m^2^ min^−1^) are several orders of magnitude lower than the self-diffusivity of REEs in water (3.70 × 10^−8^ m^2^ min^−1^ for Nd(III) and 3.58 × 10^−8^ m^2^ min^−1^ for Gd(III)) [65]. This means that the resistance to intraparticle diffusion could contribute to control the mass transfer of REEs (despite the limited thickness of the polymer layer and the large pore size compared with the size of REEs ions). Michelsen et al. [66] discriminated the systems controlled by resistance to film diffusion and pore diffusion by the order of magnitude of the effective diffusivity—film diffusion predominates for D_eff_ in the range 6 × 10^−9^ m^2^ min^−1^ to 6 × 10^−11^ m^2^ min^−1^, while resistance to pore diffusion operates when D_eff_ ranges between 6 × 10^−14^ m^2^ min^−1^ and 6 × 10^−16^ m^2^ min^−1^. The current values of the effective diffusivity clearly fall in the range of diffusivities associated with film diffusion limitations. The pore radius (around 333 Å; the average value for the data derived from sorption and desorption branches of N2 isotherm, Figure S1) is much larger than the radius of REE hydrated species (around 1.1 Å, Table S8); this may explain the limited effect of resistance to intraparticle diffusion and the fast kinetics.

#### 2.2.4. Sorption Isotherms

**Figure 8:**
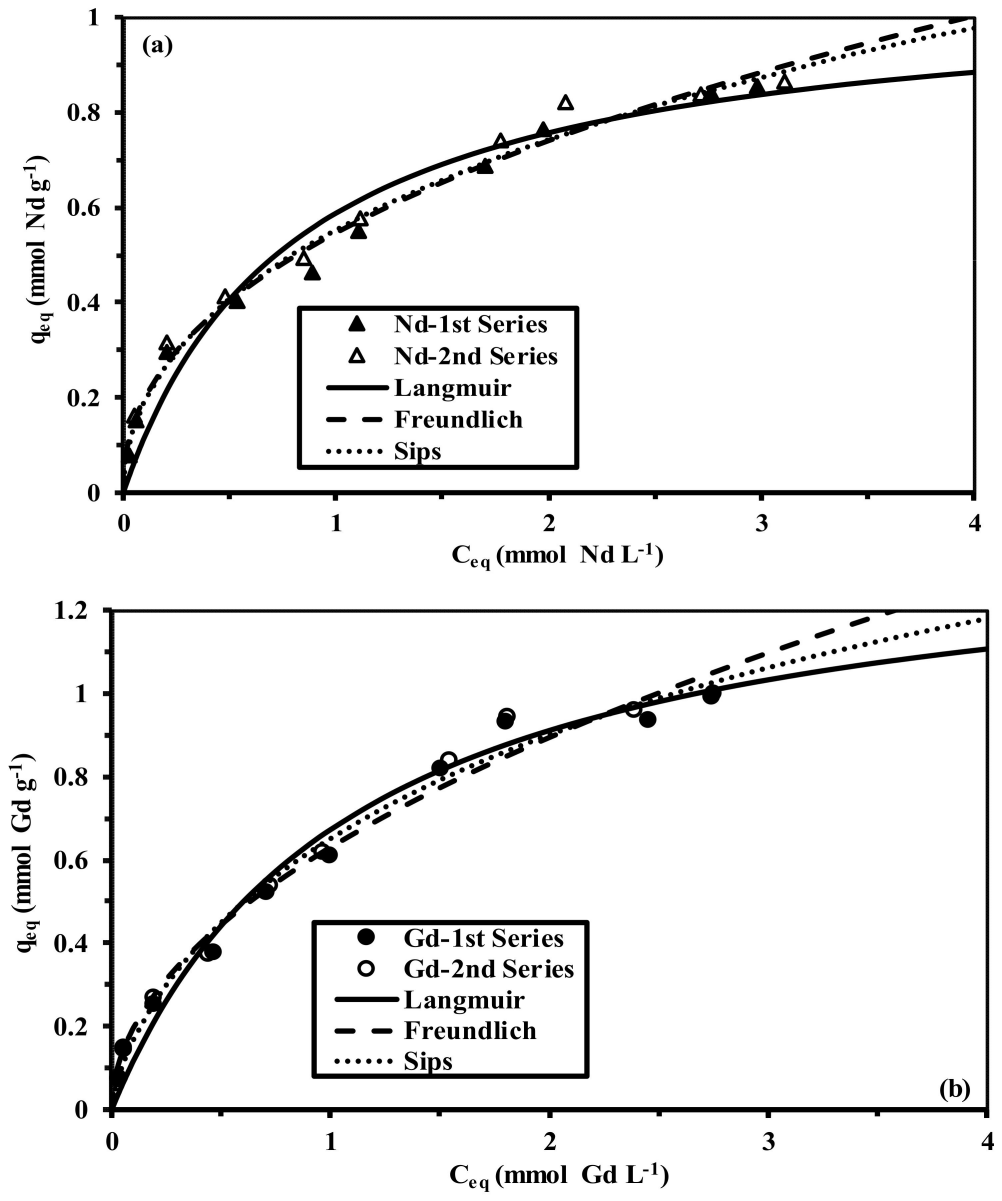
Nd(III)(**a**) and Gd(III)(**b**) sorption isotherms using mesoporous composite functionalized sorbent—modeling with the pseudo-first-order rate equation (C_0_: 0.62–3.54 mmol metal L^−1^; pH_0_: 5; pH_eq_: 4.69–4.48 for Nd(III) and 4.61–4.7 for Gd(III); SD: 0.5 g L^−1^; agitation speed: 170 rpm; T: 22 ± 2 °C).

The sorption isotherms represent the distribution of the solute between liquid and solid phases at equilibrium at a fixed temperature (and pH). The sorption capacity is plotted against equilibrium concentration at pH_0_ 5 (Figure 8). The superimposition of the series (duplicate experiments) confirms the reproducibility of sorption performances. The sorption capacity progressively increases and reaches a saturation plateau for residual concentrations in the range 2–2.5 mmol L^−1^. The initial slopes are not very steep; this means that the sorbent does not have a strong affinity for both Nd(III) and Gd(III). The Langmuir, Freundlich, and Sips equations are compared in Figure 7 and Table 2.

The Langmuir equation fits better the profiles of the isotherms as shown by the determination coefficients. However, the comparison of AIC values shows conclusions that are more debatable. For Gd(III), the three models have comparable values of AIC, and the differences are not meaningful; it is usually accepted that differences become significant when ǀ∆AICǀ > 2. In the case of Nd(III), the Freundlich equation gives a much lower AIC value than Langmuir and Sips equations. A more detailed discussion shows that the Freundlich and the Sips equations fit better the initial section of the curves (at low residual concentrations, i.e., below 1 mmol Nd(III) L^−1^ and 1.5 mmol Gd(III) L^−1^), but fail to represent the saturation plateau. The Langmuir equation was developed to describe systems where the sorption occurs as a monolayer without interactions between sorbed molecules and with homogeneous binding energies [67]. Therefore, the sorbent can be considered homogeneous in terms of reactive groups involved in metal binding. This equation is mechanistic due to being based on the equilibrium of sorption and desorption kinetics (with reference to occupied and free reactive sites, respectively). On the other hand, the Freundlich equation is purely empirical; this model was frequently associated with phenomena involving multilayer sorption and with possible interactions between sorbed molecules and non-uniform distribution of heats of sorption—the sorption involves different reactive groups and/or the sorption of different species (having different affinities for reactive groups). The Sips equation combines Langmuir and Freundlich equations, where the parameter *n_S_* is correlated with the heterogeneity of sorbent surface. At low metal concentrations, the preferential fit of experimental profiles with the Freundlich and Sips equation means that the heterogeneity of the surface affects metal binding. However, when the concentration of uranium increases the sorption tends to become more homogeneous (with the appropriateness of the Langmuir for describing the saturation plateau of the isotherms). The *n_S_* coefficient is the reciprocal of heterogeneity factor reported by Anirudhan and Radhakrishnan [67]—*n_S_* decreased from 1.985 for Nd(III) to 1.437 for Gd(III); it means that the heterogeneity of the sorbent in terms of interaction with REEs is more marked for Gd(III) than for Nd(III).

The maximum sorption capacities for Nd(III) and Gd(III) are close to 0.87 mmol g^−1^ and 1 mmol g^−1^, respectively. These values are lower than the sorption capacities at saturation of monolayer (as deduced from Langmuir equation), which are 1.06 mmol Nd g^−1^ and 1.41 mmol Gd g^−^^1^. These orders of magnitude (0.87–1 mmol g^−1^) are consistent with the fraction of primary amine groups on the sorbent (deduced from the expected structure and from elemental analysis), which is 1.08 mmol NH_2_ g^−1^. This signifies that the probable sorption mechanism involves the interaction of one protonated (primary) amine groups with a single REE ion (probably under the form REE(SO_4_)^+^), and the coordination sphere being completed by other interactions with OH groups on the sorbent or from water molecules). The affinity coefficient (i.e., b_L_) for Nd(III) is a little higher (i.e., 1.24 L mmol^−1^) than the corresponding value for Gd(III) (i.e., 0.913 L mmol^−1^). It is noteworthy that the coefficient q_m,L_ × b_L_ (L g^−1^), which corresponds to the initial slope of the sorption isotherm, is almost independent of the metal ion (i.e., 1.317 L g^−1^ for Nd(III) and 1.286 L g^−1^ for Gd(III)). The ionic radius of hydrated Nd(III) and Gd(III) and their electronegativities are very close; this cannot explain the little higher sorption capacities reached with Gd(III). The only significant difference is related to the coordination number, which is higher for Nd(III) (i.e., 9) than for Gd(III) (i.e., 8).

Tables S9 and S10 compare the sorption performances of a series of sorbents for Nd(III) and Gd(III), respectively. Some sorbents have remarkable performances, such as calixarene-functionalized graphene oxide composite, [68] carboxylic acid-modified corn stalk gel for Nd(III) [69], carbon nanotubes/graphene oxide [70], amino-phosphonic functionalized hollow silica nanospheres [71], or DTPA-chitosan/magnetite for Gd(III) [72]. Except for these “super-adsorbents,” the composite functionalized sorbent (NH_2_/SiO_2_) show comparable sorption properties for the two REEs with the most efficient sorbents. The combination of good sorption capacities with fast kinetics makes this material very promising for REE recovery from slightly acidic solutions. The less favorable parameter of this material remains its weak affinity coefficient.

#### 2.2.5. Competitive Sorption and Selectivity

Multi-components solutions containing equimolar concentrations (1 mmol each metal L^−1^) of alkali-earth metal ions, i.e., Ca(II), Mg(II)), and Sc(III) (frequently associated to REE family), in addition to Nd(III) and Gd(III). These experimental conditions are not supposed to reflect the composition of real effluents realistically; the objective is to evaluate the selectivity of the sorbent for target metals against base metals under identical conditions. Figure 8 reports the selectivity coefficients of Nd(III) against other metal ions (Figure 8a) and Gd(III) against other co-existing metal cations (Figure 8b) at different equilibrium pH values. The selectivity coefficient is calculated as the ratio of the distribution ratio (D, q_eq_/C_eq_, L g^−1^) of target metal against the distribution ratio of the competitor metal ion SC_Nd/Metal_ = D_Nd_/D_metal_.

**Figure 9:**
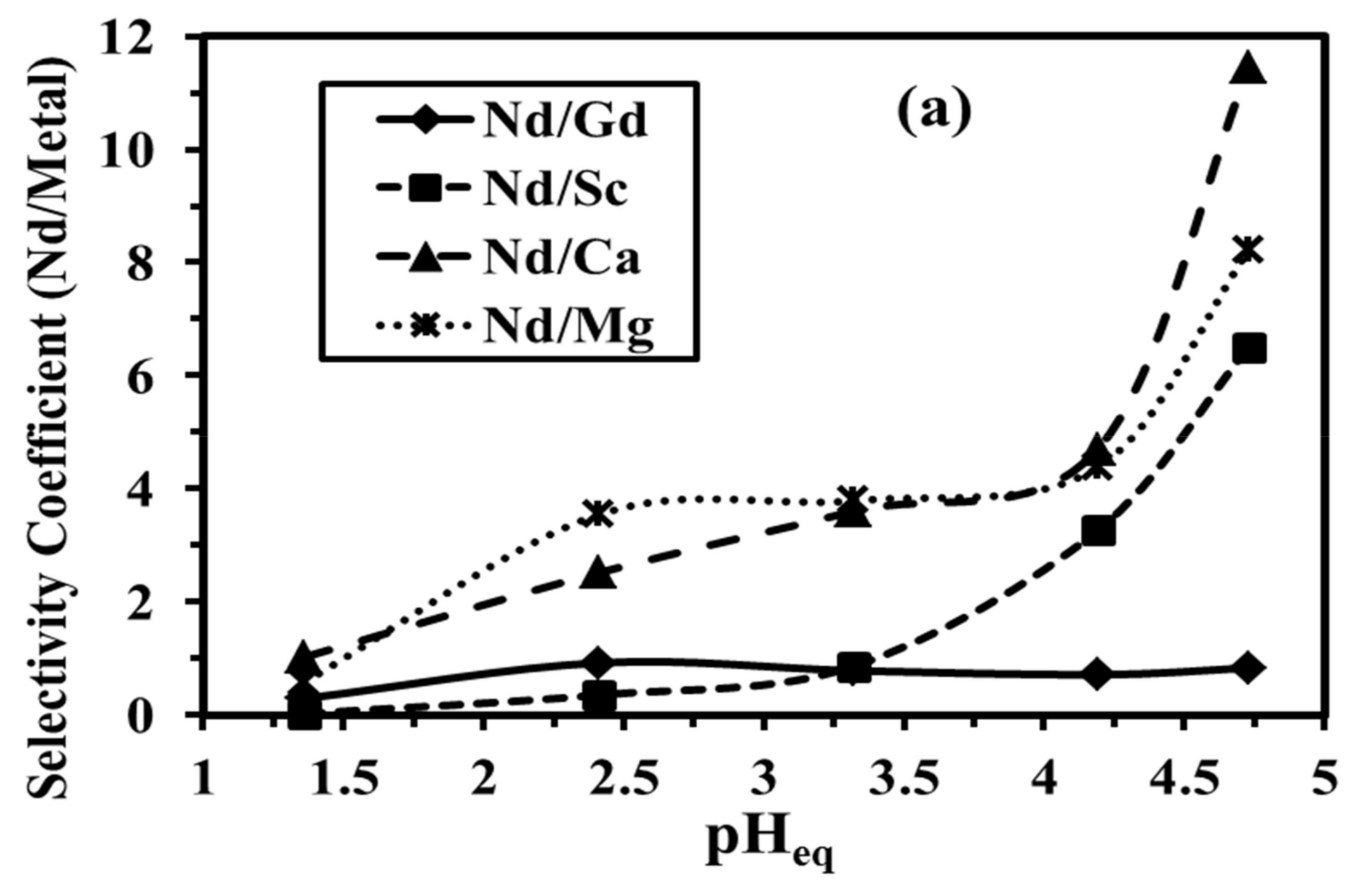

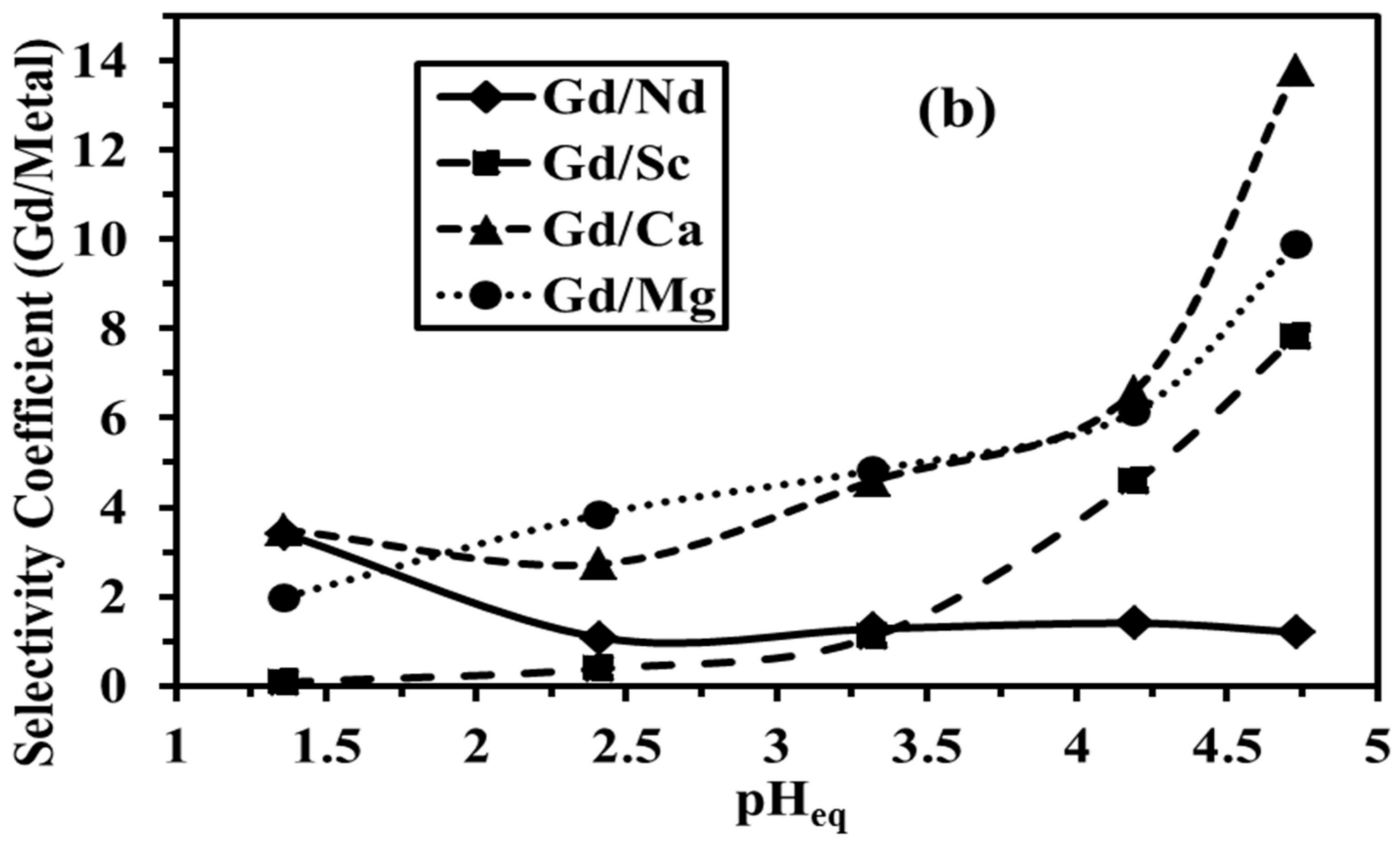
Sorption of Nd(III) and Gd(III) from multi-metal solutions—selectivity coefficient versus equilibrium pH (C_0_: 1 mmol metal L^−1^; pH_0_: 1–5; pH_eq_: 1.36–4.73; SD: 0.125 g L^−1^; agitation speed: 170 rpm; T: 22 ± 2 °C).

Previous characterizations (pH effect, uptake kinetics, and sorption isotherms) have shown the close similarities in the sorption behavior of Nd(III) and Gd(III); the sorption of these REEs from multi-component solutions confirm the difficulty for their mutual separation. Indeed, SC_(Nd/Gd)_ remains between 0.29 and 0.92—the two metals cannot be separated; the greatest selectivity is achieved at pH 1.36 with a preference for Gd(III) over Nd(III) as shown in Figure 9. However, the sorption capacities at pH 1.36 do not exceed 0.036 mmol Gd g^−1^. On the other hand, the sorbent has a marked preference for Nd(III) and Gd(III) over alkali-earth metal ions, especially increasing at the highest pH value—the SC values vary between 8.2 and 13.8 at pH 4.76. The behavior of Sc(III) is completely different: the sorbent is selective of Sc(III) at pH 1.36 with SC values ranging between 12.1 (against Gd(III), lowest selectivity) and 41–42 (against Nd(III) and Ca(II), highest selectivity). When the pH increases, the sorbent progressively loses its selectivity for Sc(III), especially against REEs (as commented above, SC_Sc/Nd_ or Gd being the reciprocal of SC_Nd_ or _Gd/Sc_). These differences are also illustrated by the enrichment factor (EF = q_eq_/C_0_, L g^−1^) (Figure S11a)—high enrichment is obtained in acidic conditions for Sc(III) (up to 290 L g^-1^) and in weakly acidic conditions (i.e., pH 4.73) for Nd(III) and Gd(III) (up to 237–268 L g^−1^). Based on the choice of the initial metal concentration of metal ions (i.e., 1 mmol L^−1^), the EF is homothetic of the sorption capacity of the sorbent for selected metals, expressed in µmol g^−1^. The cumulative sorption capacity for the five metal ions increases from 0.375 mmol g^−1^ at pH 1.36 (mainly due to Sc(III) sorption) up to 0.636 mmol g^−1^ at pH 4.73 (essentially Nd(III) and Gd(III)). It is noteworthy that at pH 4.73 is the optimum pH for selective recovery of REEs, the residual concentration, C_eq_, tends to 1.99 mmol (Nd + Gd) L^−1^. Based on sorption isotherm (Figure 8), for this residual concentration, the expected sorption capacity would be 0.76 mmol Nd g^−1^ or 0.91 mmol Gd g^−1^. The lowest sorption capacity means that the other metal ions directly compete with Nd(III) and Gd(III) for binding on the same sorption sites (despite the preference for REEs). Figure S11b shows the log_10_ plots of the distribution coefficients versus pH_eq_. Very low values are observed for alkali-earth metal ions, while the plots are roughly linear in the range pH 2.41–4.73; the positive slope is comparable for Nd(III) and Gd(III) (around 0.22–0.24), while Sc(III) shows a negative slope (close to −0.3). This confirms the opposite trends for Sc(III) versus Nd(III) and Gd(III), although Sc(III) is frequently associated with the REEs family due to very similar chemical properties, as shown in Table S8. Figure S12 plots the speciation diagrams for the five metals ions (in multi-component solutions and under selected experimental conditions). Nd(III) and Gd(III) show similar species distributions, while Sc(III) shows a greater sensibility to the pH and the formation of hydrolyzed species at pH above 4 (contrary to REEs).

Table S11 shows the SEM micrographs and the semi-quantitative EDX analysis of the surface of beads after being in contact with the multi-component solution at pH 5 (equilibrium pH 4.73). This is roughly consistent with the trends discussed above; the binding of metal ions is ranked according the series (in terms of atomic percentage, %): Gd(III)(1.34) > Sc(III)(1.07) ≈ Nd(III)(0.96) >> Ca(II)(0.27) >> Mg(II)(0.06). The main discrepancy concerns the relatively high fraction of scandium at pH 5 contrary to the weak Sc(III) enrichment factor reported in Figure S11a.

#### 2.2.6. Metal Desorption and Sorbent Recycling

**Figure 10:**
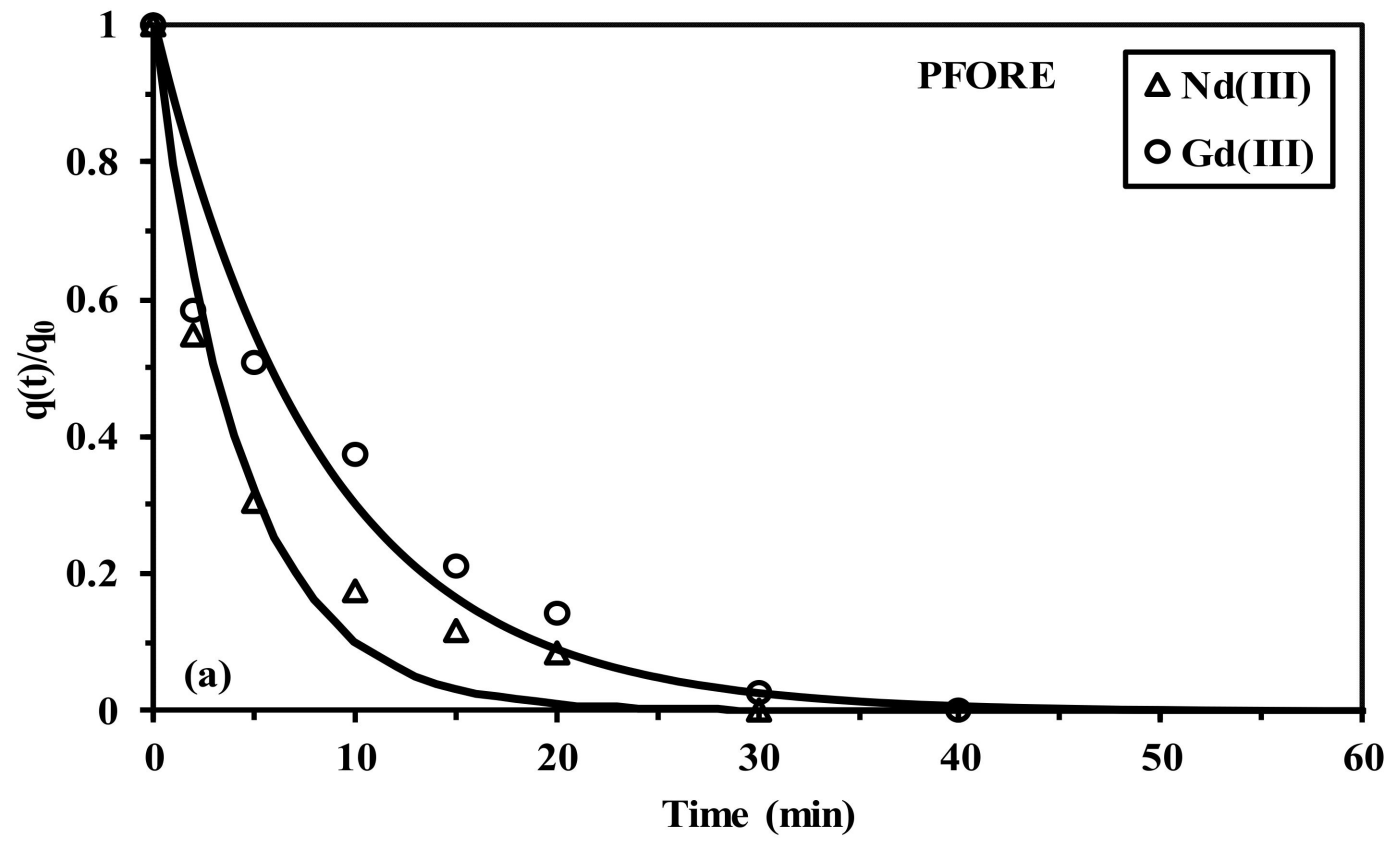

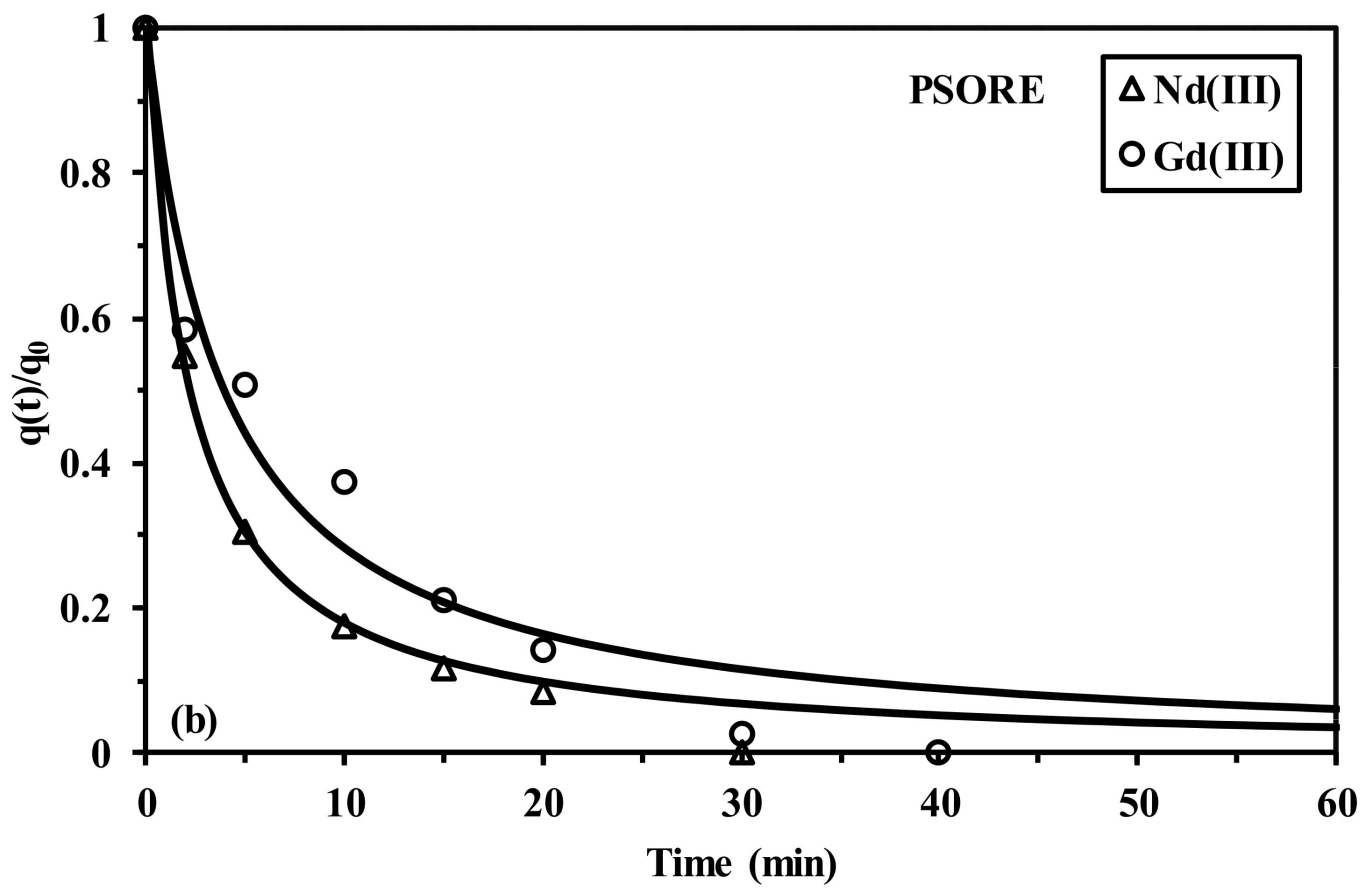
Desorption kinetics for Nd(III) and Gd(III) loaded on mesoporous composite functionalized sorbent—modeling with (**a**) the pseudo-first-order rate equation (PFORE) and (**b**) the pseudo-second-order rate equation (PSORE) applied to desorption profiles (loaded samples obtained from uptake kinetic experiments; eluent: 0.5 M HCl solution; SD: 1.25 g L^−1^; agitation speed: 170 rpm; T: 22 ± 2 °C).

The sensitivity of Nd(III) and Gd(III) sorption to low pH values is a first incentive to use acidic solutions for the elution of metal loaded on the composite functionalized sorbent. Hydrochloric acid solutions (0.5 M) are used for testing the desorption of REEs. Desorption kinetics are reported in Figure 10. The contact time for achieving the complete elution of the metals ranges between 30 min and 40 min. Desorption and uptake kinetics are comparable in terms of equilibrium time. Figure 10 shows the modeling of the desorption profiles with the PFORE and PSORE [73]; the parameters of the models are summarized in Table 3. The plots of the simulated curve fail to describe the complete time range—the PSORE fits well the fast initial section of the kinetic profiles, while the PFORE succeeds in fitting the final section of the curves (corresponding to complete desorption). The comparison of the apparent rate coefficients (for both k_D1_ and k_D2_) shows a reciprocal trend compared with sorption: the coefficients for Nd(III) desorption are almost two times greater than those of Gd(III) elution (contrary to uptake kinetics).

Table 4 compares the sorption and desorption efficiencies for both Nd(III) and Gd(III) along with five successive cycles. The sorbent shows remarkable stability in sorption performance: the decrease does not exceed 3% over 69% (i.e., an effective loss of about 4%). The stability of the sorbent is even better in terms of desorption efficiencies—the desorption is affected by the number of cycles. These results demonstrate the high stability of the sorbent. This is confirmed by SEM observations and EDX analyses of the sorbent after metal elution (Table S7) that shows the binding of chloride (dissociation of HCl), and after the fifth regeneration (Table S12). After metal elution, the Cl element (for chloride) replaces the S element (for sulfate).

### 2.3. Treatment of Industrial Effluent (PPLS)

Table S13 summarizes the composition of the gibbsite ore after burning at 800 °C (oxidative roasting). Alumina and silica represent more than 58% of the solid, iron oxide counts for about 7%, while alkali and alkali-earth oxides represent 23.5%. Table 5 compares the semi-quantitative EDX analyses of the ore (pristine) with the surface composition of the sorbent after being exposed to the PPLS (pH_0_: 5), and the oxalic acid precipitate (elaborated from the eluate of the sorbent; 800 °C—calcined precipitate). Table S14 shows the EDX spectra of ore and final REE oxides.

From Table 5, it appears that the leaching and pH control combined with the sorption on the composite functionalized sorbent allows binding the REE family without selectivity but allows discriminating most of the alkali, alkali-earth, and heavy metals (with the exception of Cu, Zn, and Al, appearing with atomic fractions in the range 0.11–0.15). This series of treatments allows selectively concentrating the REEs at the surface of the sorbent. Intermediary analyses were not carried out; it is thus difficult to evaluate the respective contributions of (a) the leaching, (b) the pH control (precipitation), and (c) the sorption steps. After elution of the metals from loaded sorbent, oxalic-acid precipitation and calcination, EDX analysis show that the produced material is mainly constituted of REE_2_O_3_. The sole impurity identified in the EDX spectrum is alumina but its fraction is negligible (about 0.01 %, atomic fraction). Focusing the attention on the REEs, Figure S13 shows the relative percentage of the individual REEs in the three materials (ore, loaded sorbent, and raffinate), considering specifically the REEs contents. In the ore, the most represented REEs are Nd (29.0%) >> Gd (12.6%) > Pm (9.5%) > Ho (6.9%) > Sm (6.5%) ≈ La (6.5%) > Eu (6.1%) > Dy (5.7%) > Er (3.4%) > Pr (3.1%) ≈ Tb (3.1%) ≈ Lu (3.1 %); other REEs represent less than 1.5 % of total REEs. After pre-treatment (acid leaching, pH control at 5, and sorption), the differences in the atomic fractions present at the surface of the sorbent tend to level off: the atomic fractions of marginal REEs are substantially increased, especially with Ce, Tm, Yb.

In the raffinate (after elution, oxalic acid precipitation and burning), the major elements are Nd (50.6%) >> Gd (25.4%) >> Sm (8.5%) >> Y (3.1%) > Pr (2.8%) > Ce (2.5%) > Eu (2.1%) > La (2.0% > Pm (1.0%). Other REEs (most of them being heavy REEs (HREEs)) are not detected (such as Tb) or represent less than 0.5 % (total fraction of these HREEs, is close to 2% of total REEs). The process is thus capable of purifying the REE concentrate from heavy metals but it is also strongly enriching the relative fraction of lighter REEs (76% being constituted of Nd and Gd).

This concentration effect (versus ore) is highlighted in Figure S14. The pretreatment of the ore and the sorption process concentrate Ce, Tm, and Yb (factor close to 3). The complete process has a strong concentration effect (higher than 20 times) for Ce, Y, Gd, Nd, Sm, and Pr.

## 3. Materials and Methods

### 3.1. Materials

Silica particles were supplied by Asahi Chemicals, Co Ltd. (Osaka, Japan). Glycidyl methacrylate (GMA), *N*′–methylenebis(acrylamide) (MBA), and azobis isobutyro nitrile (AIBN) were purchased from Sigma Aldrich (Taufkirchen, Germany). Methyl benzoate (MB), diethylenetriamine (DETA), and polyvinyl alcohol (PVA) were supplied from Sinopharm Chemical Reagent Co. Ltd. (Shanghai, China), dioctyl phthalate (DPh), Nd(III) sulfate (Nd_2_(SO_4_)_3_.5H_2_O), and Gd(III) sulfate (Gd_2_(SO_4_)_3_.8H_2_O) were supplied by National Engineering Research Centre of Rare Earth Metallurgy and Functional Materials Co., Ltd. (Baotou Rare Earth Development Zone, Inner Mongolia, China). Scandium sulfate, calcium chloride, and magnesium sulfate were purchased from Guangdong Guanghua Sci-Tech Co. (Haizhu District, Guangzhou, China). All other chemicals were used as received and supplied by Prolabo-France, (79, rue des Morillons, 75015 PARIS, France métropolitaine). The experiments were performed using freshly prepared solutions by dilution of the stock solutions (1 g L^−1^) with Milli-Q (Wolfgang-Kuentscher Str. 14, 16761 Hennigsdorf, Germany) water to the desired concentration. The pH was adjusted using 0.1/1 M H_2_SO_4_ and NaOH solutions.

### 3.2. Synthesis of Sorbent 

#### 3.2.1. Synthesis of Base Polymer/SiO_2_ Mesoporous Composite

Grafting of GMA on silica particles (85:100 µm) was performed by a polymerization reaction in the presence of the crosslinking agent (MBA). The silica to polymer and to the total aqueous solution (including monomers and diluents) was set around 4:1 and 1:6 (*w*/*w*), respectively. The polymerization reaction was performed by dissolving 0.5 g of AIBN in a mixture of 10 g of GMA and MBA with a ratio of 9.0:1.0 (*w*/*w*), respectively. This mixture was added drop-wise to a mixture of 40 g silica immersed in 120 g of pore producing solvent (40 g methyl benzoate/80 g dioctyl phthalate), 90 mL 1% PVA, and 20 mL of 0.1% methylcellulose. Monomers were progressively added in the reactor (for 30 min) under strong agitation (speed 230 rpm) at 40 °C. The temperature was increased to 90 °C under continuous agitation for 10 h in an oil bath. The product was filtered off, washed several times with hot water and acetone for removing unreacted substituents and diluents. The solid was finally dried in air at 60 °C for 8 h to produce SiO_2_-glycidyl methacrylate-methylenebisacrylamide (Scheme 1; GMA-MBA/SiO_2_).

#### 3.2.2. Functionalization of Mesoporous GMA-MBA/SiO_2_

The composite was functionalized by the incorporation of amine groups through the grafting of DETA. This was performed by mixing 1 g of GMA-MBA/SiO_2_ with 3 mL of DETA in 20 mL of 1 M Na_2_CO_3_ solution, under reflux at 80 °C for 10 h in an oil bath. After functionalization, the material was washed by water, ethanol and dried at 50 °C for 10 h. Scheme 1 summarizes the reaction pathway for producing the composite functionalized sorbent (DT-GMA-MBA/ SiO_2_).

### 3.3. Sorbent Characterization

FTIR analysis was performed in the range of 400–4000 cm^−1^ using a Shimadzu IRTracer-100 FTIR spectrometer (Shimadzu, Kyoto, Japan). TGA-DSC of composite functionalized sorbent was measured using a Netzsch STA 449 F3 Jupiter (NETZSCH-Gerätebau GmbH, Selb, Germany), the process was performed under N_2_ atmosphere (in a platinum cell, with a heating ramp of 10 °C/min). Elemental analysis for H, C, and N was achieved using PE2400II elemental analyzer (PerkinElmer, Waltham, MA, USA). The contents of amine grafted groups were detected volumetrically by titration that is briefly detailed as follows: a mixing of 0.1 g of sorbent with 30 ml of 0.05 M HCl (C_HCl,1_) was agitated for 15 h. The concentration of HCl (C_HCl,2_) was determined by titration against 0.05 M NaOH solution in the presence of a pH indicator (phenolphthalein). The amine concentration was determined by the mass balance equation as (–NH_2_) = (C_HCl,1_ − C_HCl,2_) × 30/0.1.

Textural analysis (BET surface area data and porosity) was carried out using a TriStar II 3020 surface area and porosity system (Micromeritics Instrument Corporation, Norcross, GA, USA) under N_2_ atmosphere; the sorbent sample was degassed firstly at 120 °C for 12 h under N_2_ flow. The morphological structure and the chemical composition (semi-quantitative analysis) of the different materials were investigated using SEM-EDX analysis on Phenom ProX desktop SEM with an accelerating voltage of 15.0 kV (ThermoFisher Scientific, Waltham, MA, USA) equipped with the integrated elemental identification system. XPS spectra were analyzed using ESCALAB 250XI spectrometer (ThermoFisher Scientific, Waltham, MA, USA). pH_in_ (initial pH value) and pH_eq_ (equilibrium pH) were determined using an S220 Seven Compact/ Ionometer (Mettler Toledo, Colombus, OH, USA). pH_PZC_ was measured using the pH-drift method, 100 mg of material were mixed with 50 mL of 0.1 and 1 M NaCl solutions, at different initial pH values (pH_0_ = 1−11); pH_eq_ was determined after shaking for 48 h. The pH_PZC_ was defined by the pH corresponding to pH_0_ = pH_eq_.

The size of raw silica particles (SiO_2_ spheres) ranged between 85 µm and 100 µm. Table S1 (Supplementary Materials) shows SEM microphotographs of mesoporous silica particles before and after functionalization.

### 3.4. Sorption Studies

Sorption experiments were carried out first in batch systems with synthetic solutions composed of single-component solutions (or multi-component solutions for investigating sorption selectivity). This step was used for evaluating the impact of experimental parameters and the global sorption properties. The metal-containing solution (V, L) at a fixed concentration (C_0_, mmol L^−1^) and fixed initial pH (pH_0_) was mixed with a given amount of sorbent (m, g) corresponding to a sorbent dosage (SD, g L^−1^ = m/V). The agitation speed was set to 170 rpm at room temperature (22 ± 2 °C). After fixed contact times (for uptake kinetics) or after 48 h of contact (for sorption isotherms), samples were collected, the equilibrium pH (pH_eq_) was monitored, and the residual concentration (C_eq_, mmol L^−1^) in filtrated solutions was analyzed by inductively coupled plasma atomic emission spectrometry (ICP-AES, ICPS-7510 Shimadzu, Kyoto, Japan). The sorption capacity (q_eq_, mmol g^−1^) was calculated by the mass balance equation q_eq_ = (C_0_ − C_eq_) × V/m. For desorption studies, a similar procedure was used for eluting metal ions from loaded sorbents. Elution was carried out using 0.5 M HCl solutions. After equilibrium time (determined after investigating desorption kinetics), samples were collected and filtrated for determining the amount of metal eluted. The yield of desorption was determined by comparison of the amounts of target metal initially sorbed with the amount released in the eluate. Sorption and desorption yields were compared for five successive sorption/desorption cycles. The sorbent was washed up with demineralized water between each sorption and desorption step.

These general procedures were used for the different experiments (single-/multi-component solutions, sorption/desorption). The detailed experimental conditions changed for the different parts of the study, and the captions of the figures systematically report the actual processing parameters.

Tables S15 and S16 summarize the conventional equations for modeling uptake kinetics (pseudo-first and pseudo-second-order rate equations (PFOREs and PSOREs, respectively) and the Crank equation, i.e., resistance to the intraparticle diffusion equation (RIDE)) and sorption isotherms (Langmuir, Freundlich, and Sips equations), respectively. Parameters were obtained by non-linear regression analysis using Mathematica^®^ tools (Version 4.0, the software was developped by Wolfram Research/https://www.wolfram.com/mathematica/ (accessed on 9 December 2020)). Metal speciation diagrams were built using VisualMinteq [74].

### 3.5. Processing of Industrial Solution

In order to evaluate the potential of this sorbent for treating real effluents, the last part of the study focuses on the treatment of acidic ore leachates. Ore processing was performed on a gibbsite sample collected in the Abu Mogherat mining area (southwestern Sinai, Egypt, Figure S15, see Supplementary Materials Section). Initial REEs content was 1.695% (*w*/*w*). After calcination of the sample at 800 °C for 2 h (oxidative roasting), weight loss was close to 30%, and REEs content increased to 2.35% (*w*/*w*). Table S14 reports the main constituents of the ore after the burning step (with relevant analytical procedures).

The material was subjected to sulfuric acid leaching, and 100 g of ore sample was reacted with 400 mL of sulfuric acid solution (200 g/L) in stirred tank reactor for 3 h at 100 °C. The pH of the leachate (pregnant liquor solution (PLS)) was finally controlled at pH 5 using NaOH solution; Al and Fe are partially precipitated. The precipitated pregnant leachate (PPLS) contained around 4850 ppm of REEs.

Sorption test was carried out by mixing 150 mL of PPLS with 5 g of composite functionalized sorbent for 2 h. The sorbent was filtered and washed with demineralized water before processing to the elution. Elution was carried out using 2 M HCl. The pH of the eluate was controlled to 1.5 by NaOH/ HCl solutions and treated with oxalic acid (15 mL/10% *w*/*w* oxalic acid solution) for precipitation of REEs as oxalate salts. The solid was dried and burnt at 850 °C for 2 h to produce rare earth oxide Re_2_O_3_. Semi-quantitative EDX analysis was used for evaluating the content of the elements on the sorbent (after PPLS treatment), in the oxalate precipitate, and in the final REE oxide material.

## 4. Conclusions

The successful grafting of diethylenetriamine on glycidyl methacrylate-coated silica micro-beads allows producing a mesoporous amino-coated sorbent with good sorption properties for REEs in the range 0.9–1 mmol g^−1^ at pH 4. The specific surface area reaches 57 m^2^ g^−1^. Nitrogen content is close to 3.25 mmol N g^−1^, which is constituted for 33% of primary ending groups and 67% of secondary amine groups. The binding mechanism involves ion-exchange between protonated amine groups (preferentially primary amine groups) with cationic REEs species. The presence of the S element on the sorbent tends to indicate that REEs are probably bound as REE(SO_4_)^+^ species. However, it is not possible to reject the possibility that the S element could come from the direct binding of sulfate anions (from sulfate salt and/or acid dissociation). The sorbent has similar properties for the binding of Nd(III) and Gd(III); the functionalized mesoporous material cannot separate the two REEs. The uptake kinetics, which is described by the pseudo-first-order rate equation, reach the equilibrium (under selected experimental conditions) within 30–40 min of contact. The desorption kinetics are of the same order of magnitude and also require a 30–40 min contact time with 0.5 M HCl solution for complete elution of REEs. The sorbent can be recycled for a minimum of five cycles with negligible loss in performance. The mesoporous sorbent is selective for REEs (including Sc(III)) against alkali-earth elements, especially at pH 4.5. The sorbent has a marked preference for Sc(III) against Nd(III) and Gd(III) at pH 1.36, while the preference is reversed at pH 4.5; neodymium and gadolinium cannot be separated from each other. The acidic leachate of a polymetallic ore (Gibbsite) is successfully treated using the composite functionalized sorbent: the combination of pH control, sorption on NH_2_/SiO_2,_ and acidic elution, followed by oxalic precipitation and calcination, allow us to produce a pure REEs raffinate (constituted from more than 99.9% of REE_2_O_3_), which is substantially enriched in lighter REEs.

## Figures and Tables

**Table 1:** Nd(III) and Gd(III) uptake kinetics—parameters for models.

Model	Parameter	Nd(III)		Gd(III)	
Series		1	2	1	2
Experimental	q_eq,exp_ (mmol g^−1^)	0.389	0.373	0.374	0.367
PFORE	q_eq,1_ (mmol g^−1^)	0.411	0.394	0.376	0.366
	k1 × 10^2^ (min^−1^)	7.11	6.92	12.2	11.0
	*R* ^2^	0.945	0.944	0.995	0.993
	AIC	−120	−119	−175	−164
PSORE	q_eq,1_ (mmol g^−1^)	0.489	0.470	0.420	0.409
	k2 × 10^2^ (L mmol^−1^ min^−1^)	16.3	16.3	40.5	38.7
	*R* ^2^	0.902	0.901	0.979	0.988
	AIC	−113	−112	−144	−146
RIDE	D_eff_ × 10^11^ (m^2^ min^−1^)	4.71	4.61	7.18	6.49
	*R* ^2^	0.915	0.913	0.970	0.996
	AIC	−117	−115	−169	−164

**Table 2:** Nd(III) and Gd(III) sorption isotherms—parameters for models.

Model	Parameter	Nd(III)	Gd(III)
Experimental	q_max_. (mmol g^−1^)	0.866	1.000
Langmuir	q_m,L_ (mmol g^−1^)	1.063	1.408
	b_L_ (L mmol^−1^)	1.239	0.913
	*R* ^2^	0.971	0.979
	AIC	−119	−114
Freundlich	k_F_	0.546	0.631
	n_F_	2.285	1.980
	*R* ^2^	0.987	0.984
	AIC	−143	−114
Sips	q_m,S_ (mmol g^−1^)	4.054	2.361
	b_S_ (L mmol^−1^)	0.158	0.381
	n_S_	1.985	1.437
	*R* ^2^	0.988	0.985
	AIC	−141	−115

**Table 3:** Kinetics of desorption for Nd(III) and Gd(III)—parameters for models.

Model	Parameter	Nd(III)	Gd(III)
PFORE	k_D1_ × 10^2^ (min^−1^)	22.8	12.0
	*R* ^2^	0.980	0.952
PSORE	β2 (dimensionless)	0.994	1.014
	k_D2_ × 10^2^ (min^−1^)	46.0	25.2
	*R* ^2^	0.995	0.959

PFORE: $\frac{q\left(t\right)}{q_{0}}=\;e^{-k_{D1}t}$ with: k_D1_ the apparent rate coefficient for desorption (min^−1^) PSORE: $\frac{q\left(t\right)}{q_{0}}=\;\frac{1}{\beta_{2}+\;k_{D2}t}$ with: k_D2_ the apparent rate coefficient for desorption (min^−1^) and β2 (dimensionless).

**Table 4:** Sorption recycling—sorption and desorption efficiencies (average value and standard deviation).

Metal	Cycle	Sorption Efficiency (%)		Desorption Efficiency (%)	
		Average	St. Dev.	Average	St. Dev.
Nd(III)	#1	69.2	1.2	99.8	0.4
	#2	68.4	1.9	100.2	0.3
	#3	68.0	1.5	100.4	0.5
	#4	67.3	1.8	100.0	0.1
	#5	66.4	1.0	100.0	0.1
Gd(III)	#1	69.4	1.2	100.1	0.1
	#2	68.6	2.1	100.0	0.1
	#3	68.0	1.8	100.5	0.6
	#4	67.8	2.0	100.4	0.5
	#5	66.8	1.6	100.1	0.2

**Table 5:** Comparison of the composition of unpurified REEs ore, metal-loaded mesoporous composite functionalized sorbent, and REE oxide (after oxalic acid precipitation and calcination) —semi-quantitative EDX analysis.

Element (and Analytical Ray)	Ore		Loaded Sorbent		After Elution	
	Wt%	At%	Wt%	At%	Wt%	At%
C K	0.85	1.89	30.04	43.8	1.03	0.02
N K	0.84	0.21	3.82	4.54	0.98	0.02
O K	21.51	18.78	29.03	32.11	38.65	40.93
Si K	29.34	24.66	29.33	18.1	-	-
Al K	5.37	10.75	0.24	0.15	0.02	0.01
Ca K	7.34	6.13	-	-	-	-
Na K	5.97	9.9	0.07	0.08	0.2	0.01
Mg K	5.19	5.73	0.12	0.06	-	-
K K	0.16	0.23	-	-	-	-
Y L	0.15	0.04	0.02	0.01	1.93	1.85
La L	0.22	0.17	0.32	0.04	1.02	1.16
Ce L	0.05	0.03	0.53	0.1	2.63	1.45
Pr L	0.15	0.08	0.3	0.04	2.35	1.66
Nd L	0.97	0.76	0.22	0.13	32.62	29.83
Pm L	0.56	0.25	0.02	0.01	1.35	0.59
Sm L	0.12	0.17	0.39	0.09	4.35	5.02
Eu L	0.15	0.16	0.32	0.05	2.61	1.25
Gd L	0.51	0.33	0.14	0.02	4.33	14.96
Tb L	0.04	0.08	0.58	0.06	-	-
Dy L	0.11	0.15	0.52	0.05	0.91	0.25
Ho L	0.02	0.18	0.52	0.05	0.86	0.19
Er L	0.18	0.09	0.46	0.05	0.81	0.15
Tm L	0.07	0.03	0.74	0.08	1.01	0.2
Yb L	0.03	0.02	0.59	0.06	1.15	0.28
Lu L	0.14	0.08	0.76	0.08	1.19	0.17
V K	0.36	0.39	-	-	-	-
Fe K	3.9	6.56	-	-	-	-
Ni K	0.05	0.02	-	-	-	-
Cu K	1.43	1.09	0.49	0.13	-	-
Zn K	14.22	11.04	0.43	0.11	-	-

## Data Availability

The data presented in this study are available on request from the corresponding author.

## Supplementary Materials

**Figure S1**: Geological map for ore sampling; **Figure S2**: Textural analysis of mesoporous silica microbeads and mesoporous composite functionalized sorbent (NH/SiO_2_); **Figure S3.** Thermogravimetric analysis of mesoporous composite functionalized sorbent; **Figure S4, Figure S5**: FTIR spectra of mesoporous SiO_2_, mesoporous composite functionalized sorbent, before and after Nd(III) and Gd(III); **Figure S6**: Acid-base properties—pH_PZC_ determined by the pH-drift method; **Figure S7**: Speciation diagrams for Nd(III) and Gd(III) under the experimental conditions selected for the study of pH effect; **Figure S8**: Effect of equilibrium pH on the distribution ratio; **Figure S9**: pH variation during metal sorption; **Figure S10**, **Figure S11**: Nd(III) and Gd(III) uptake kinetics using mesoporous composite functionalized sorbent; **Figure S12**: Effect of pH on the enrichment factor, and the distribution ratio of metal ions on the mesoporous composite functionalized sorbent compared to their initial concentration in the solution; **Figure S13**: Speciation diagram for multicomponent equimolar solutions (experimental conditions corresponding to Figure 6.; **Figure S14**: Atomic fractions of REEs in unpurified ore, mesoporous composite functionalized sorbent and raffinate; **Figure S15**: Concentration factor in the loaded sorbent and in the raffinate; **Table S1**: SEM microphotographs of mesoporous silica gel particles before and after functionalization; **Table S2**: Uptake kinetics modeling—PFORE, PSORE and RIDE; **Table S3**: Sorption isotherm modeling; **Table S4**: Chemical constituents of ore sample collected from Abu Mogherat mining site after burning at 800 °C; **Table S5**: SEM micrograph and semi-quantitative EDX analysis of mesoporous silica gel particles and composite functionalized sorbent; **Table S6**: Assignements of FTIR peaks for mesoporous SiO_2_, composite functionalized sorbent:Assignments peaks and characteristic wavenumbers; **Table S7**: XPS spectra of elements present on mesoporous composite functionalized sorbent before and after sorption of target metal ions; **Table S8**: XPS analysis of signals of mesoporous composite functionalized sorbent before and after sorption of target metal ions; **Table S9**, **Table S10**: Semi-quantitative EDX analysis of mesoporous composite functionalized sorbent before and after loading with Nd(III) and Gd(III); **Table S11**: Solution-phase properties for selected metal ions—Competitive sorption from multi-component equimolar solutions; **Table S12**, **Table S13**: Sorption properties for Nd(III) and Gd(III)—Comparison of performances; **Table S14**: Semi-quantitative EDX analysis of mesoporous composite functionalized sorbent after loading with equimolar Nd, Gd, Sc, Ca and Mg solution and after treatment with polymetallic solution; **Table S15**: SEM microphotographs of mesoporous composite functionalized sorbent after 5 cycles of sorption and desorption; **Table S16**: Semi-quantitative EDX analysis of REEs ore, and after sorption/elution/oxalic acid precipitation.
