# Supplementary material for: Nd(III) and Gd(III) Sorption on Mesoporous Amine-Functionalized Polymer/SiO2 Composite
Source: Molecules. 2021 Feb 17;26(4):1049. doi: 10.3390/molecules26041049 (PMC7922550; doi:10.3390/molecules26041049)
Supplement: Supplementary file 1 [file molecules-26-01049-s001.pdf]

Nd(III) and Gd(III) sorption on mesoporous amine-functionalized polymer/SiO_2 composite

Khalid A.M. Salih,[1] Mohammed F. Hamza, [1,2] Hamed Mira,[2] Yuezhou Wei,[1,3*] Ayman M Atta[*4], Feng Gao,[1] Toyohisa Fujita,[1] Eric Guibal[5*]

[1] Guangxi Key Laboratory of Processing for Non-ferrous Metals and Featured Materials, School of Resources, Environment and Materials, Guangxi University, Nanning 530004, PR China.
[2] Nuclear Materials Authority, POB 530, El-Maadi, Cairo, Egypt.
[3] Shanghai Jiao Tong University, Shanghai, China.
[4] Chemistry Department, College of Science, King Saud University, P.O. Box 2455, Riyadh 11451, Saudi Arabia; ao_ezzat@yahoo.com
[5] Polymers Composites and Hybrids (PCH), IMT Mines Ales, Alès, France

Supplementary Material Section

Table S1. SEM microphotographs of mesoporous silica gel particles before and after functionalization.

Material	Small radius	Large radius
SiO_2	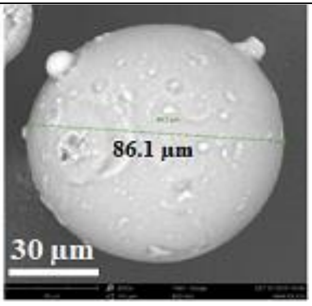	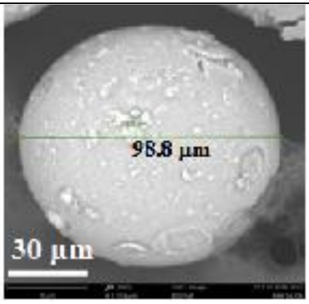
Composite functionalized sorbent	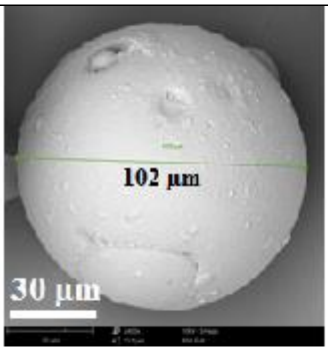	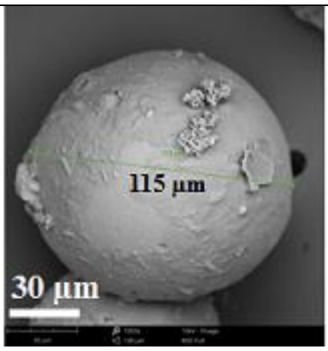

Table S2. Uptake kinetics modeling – PFORE (pseudo-first order rate equation), PSORE (pseudo-second order rate equation) and RIDE (resistance to intraparticle diffusion equation – Crank equation).

Model	Equation	Parameters	
PFORE [1]	$q(t) = q_{eq,1}(1 - e^{k_1 t})$	$q_{eq,1}$ (mg g^{-1})	k_1 (min^{-1})
PSORE [1]	$q(t) = \frac{q_{eq,2}^2 \times k_2 \times t}{1 + q_{eq,2} \times k_2 \times t}$	$q_{eq,2}$ (mg g^{-1})	k_2 (L mg^{-1} min^{-1})
RIDE [2]	$\frac{q(t)}{q_{eq}} = 1 - \sum_{n=1}^{\infty} \frac{6\alpha(\alpha+1)\exp\left(\frac{-D_e q_n^2 t}{r^2}\right)}{9 + 9\alpha + q_n^2 \alpha^2}$ With q_n being the non-zero roots of $\tan q_n = \frac{3 q_n}{3 + \alpha q_n^2}$ and $\frac{m q}{V C_o} = \frac{1}{1+\alpha}$	D_e (m^2 min^{-1})	

Akaike Information Criterion, AIC [3]:

$$AIC = N \ln\left(\frac{\sum_{i=0}^{N}\left(y_{i,exp.} - y_{i,model}\right)^2}{N}\right) + 2N_p + \frac{2N_p\left(N_p + 1\right)}{N - N_p - 1}$$

Where N is the number of experimental points, N_p the number of model parameters, $y_{i,exp.}$ and $y_{i,model}$ the experimental and calculated values of the tested variable

Table S3. Sorption isotherm modeling [4,5]

Model	Langmuir	Freundlich	Sips
Equation	$q = \frac{q_{m,L} \times b_L \times C_{eq}}{1 + b_L \times C_{eq}}$	$q = k_F\, C_{eq}^{1/n}$	$q = \frac{q_{m,S} \times b_S \times C_{eq}{}_{eq}^{1/n_S}}{1 + b_S \times C_{eq}{}_{eq}^{1/n_S}}$
Parameters	$q_{m,L}$ (mg g^{-1})*	k_F (mg$^{1-1/n}$ g^{-1} L$^{-1/n}$)	$q_{m,S}$ (mmol g^{-1})*
	b_L (L mg^{-1})**	n (dimensionless)	b_S (L mg^{-1})**
	-	-	n_S (dimensionless)

*: Sorption capacity at saturation of the monolayer; **: Affinity coefficient

Table S4. Chemical constituents of ore sample collected from Abu Mogherat mining site after burning at 800 °C.

Metal oxide	Content (%)	Metal oxide	Content (%)
SiO_2	44.98	Na_2O	3.09
Al_2O_3	13.71	K_2O	0.2
Fe_2O_3	7.33	ZnO	2.93
MnO	0.53	P_2O_5	0.18
CoO	0.0019	REEs	2.35
NiO	0.0032	U	0.0015
TiO_2	0.0011		
CuO	0.015		
MgO	8.23		
CaO	12.02		

Chemical composition of metal oxides were according the method described by Shapiro [6]: silicate was analyzed from alkaline mineralization while metal oxides (such as Al_2O_3, TiO_2, CaO, MgO, P_2O_5, and Fe oxides) were measured from acidic solution.

Five hundred mg of sample were digested using HF, HCl and HNO_3 until dissolution. The cleared solution was filtrated before being diluted with demineralized water up to 100 mL. The concentrations of metal ions (such as Ni(II), Zn(II), Mn(II), Cu(II),…etc) were determined by a Unicam atomic absorption spectrophotometer model-969 (AAS). Total REE was analyzed by a UV-VIS spectrophotometer (Shimadzu UV-160) using 0.015 % Arsenazo (III) at the wavelength λ:654 nm with reference to Y [7]. U was measured using the oxidimetric titration method by ammonium metavanadate [8].

Table S5. SEM micrograph and semi-quantitative EDX analysis of mesoporous silica gel particles and composite functionalized sorbent.

Material	SEM micrograph	EDX semi-quantitative analysis
SiO_2	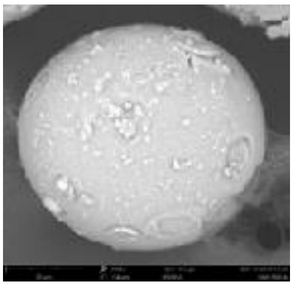	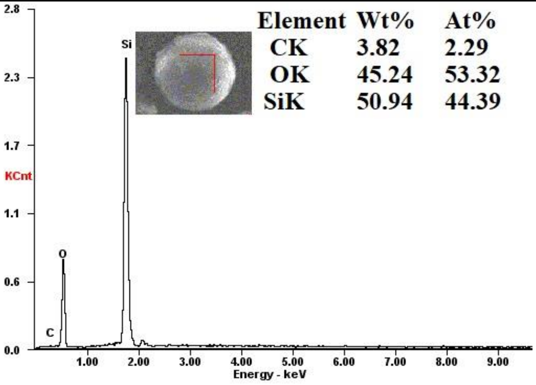
Sorbent	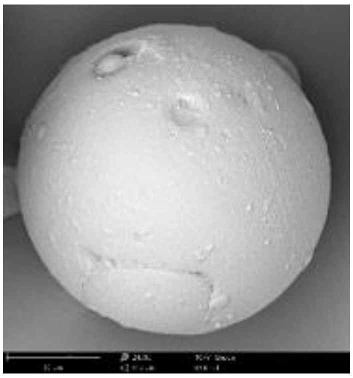	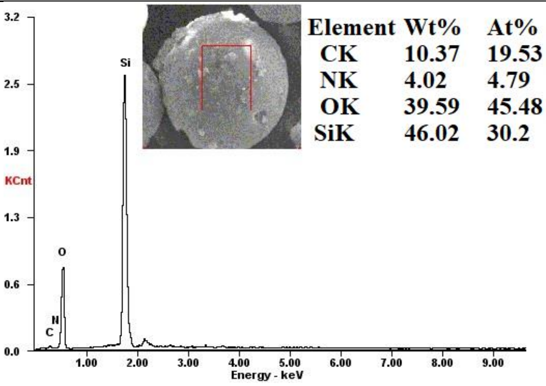

Table S6. Assignements of FTIR peaks (characteristic wavenumber, cm^{-1}) for mesoporous SiO_2, composite functionalized sorbent (before and after Nd(III) or Gd(III) sorption, and after 5 cycles of sorption/desorption) : – Assignments peaks and characteristic wavenumbers (cm^{-1}).

Vibration	Ref.	Wn. in ref.	SiO_2	NH/SiO_2	NH/SiO_2+Nd(III)		NH/SiO_2+Gd(III) After 5	
					Loaded	5 cycles elution	Loaded	5 cycles elution
O-H and N-H stretching bands	[9] [10]	3500 -3000	3439	3453	3465	3443	3518, 3159	3449
Stretching C-H aliphatic	[10-12]	2970–2950		2931	2932, 2857	2935	2939	2923
C=O stretching of ester	[11,12]	1750–1725		1738	1727	1736	overlapped	1738
C=O Stretching of amide	[11,13]	1690-1630		1632	1626	1632	1630	1635
C-N of amide and -OH bending (1º and 2º)	[14,15]	1450-1330		1452	1452	1450	==	1456
Si-O- Si bands	[12,16-19]	1095–500	1112, 802, 474	1109, 802, 472	1107, 798, 470	1109, 800, 472	1107, 800, 468	1109, 802, 470
C-N Stretching + Asymmetric C-O-C stretching	[10-12,20-22]	1090-1020		1109	1107	1109	1107	1109
C-C Stretching band	[19]	1350–1000		802	798	800	800	802

Table S7. XPS spectra of elements present on mesoporous composite functionalized sorbent before and after sorption of target metal ions.

Signal	NH/SiO_2	NH/SiO_2+Nd(III)	NH/SiO_2+Gd(III)
C *1s*	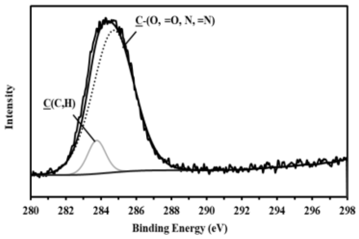	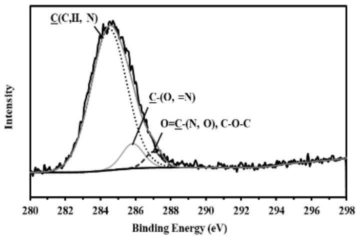	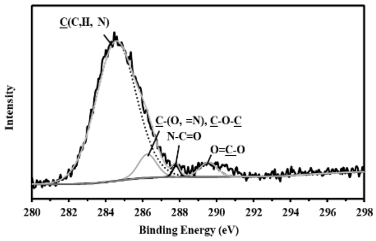
N *1s*	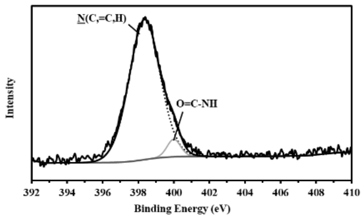	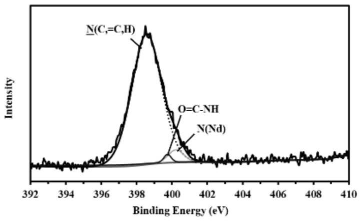	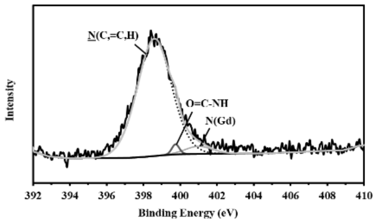
O *1s*	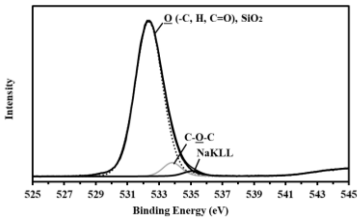	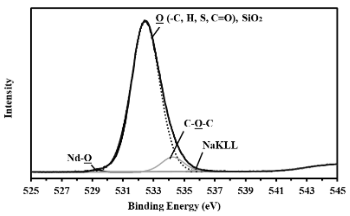	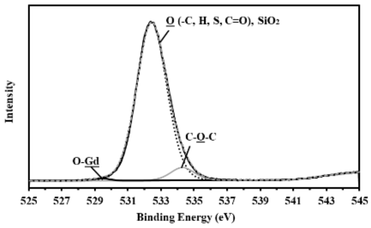
Si *2p*	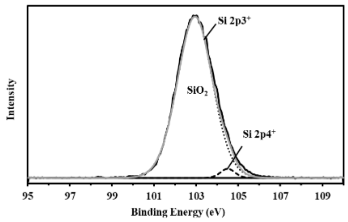	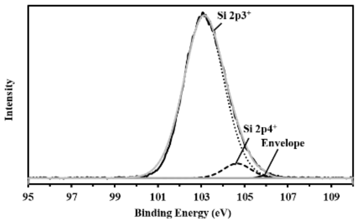	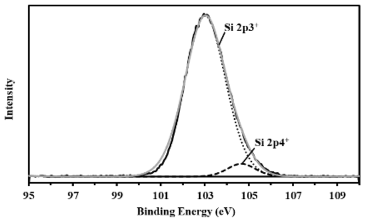
S *2p*		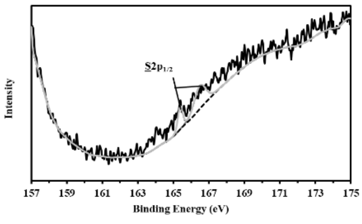	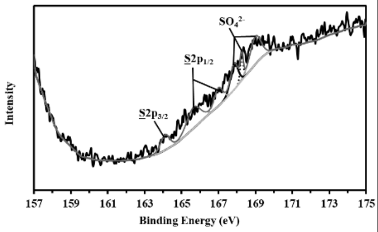
Na *1s*		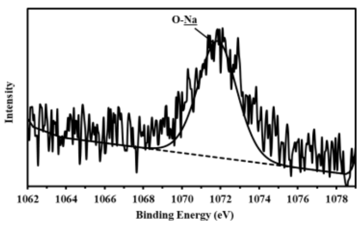	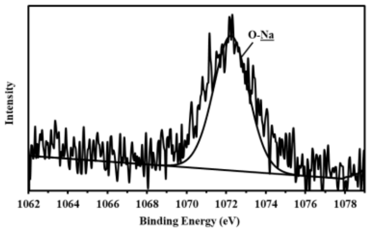

Cont' Table S7

<table>
<tr><td>Signal</td><td>$NH/SiO_2+Nd(III)$</td><td>$NH/SiO_2+Gd(III)$</td></tr>
<tr><td>M 3d</td><td>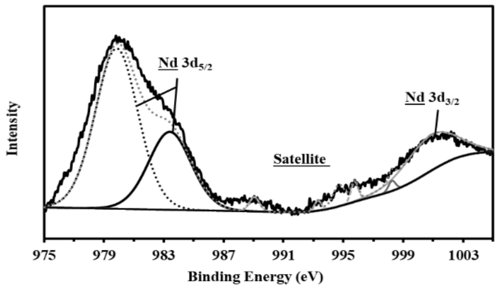
</td><td>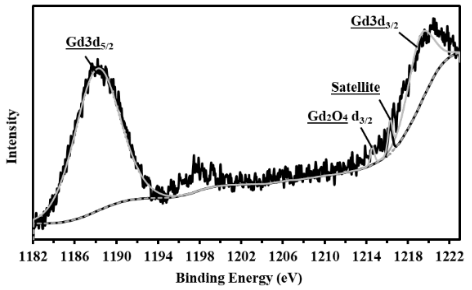
</td></tr>
<tr><td>M 4d</td><td>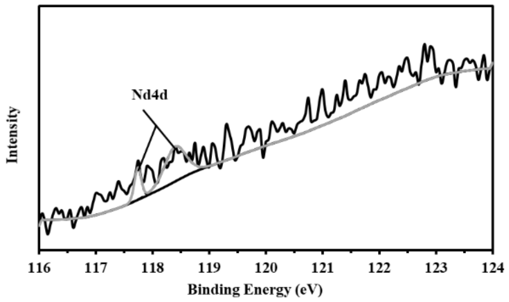
</td><td>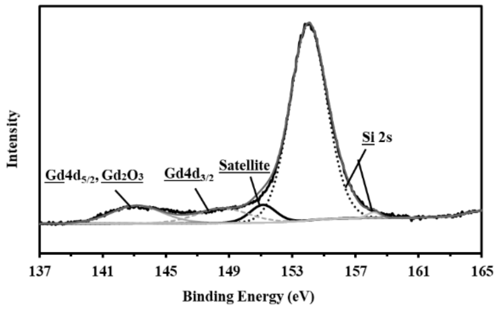
</td></tr>
<tr><td>M 4s</td><td>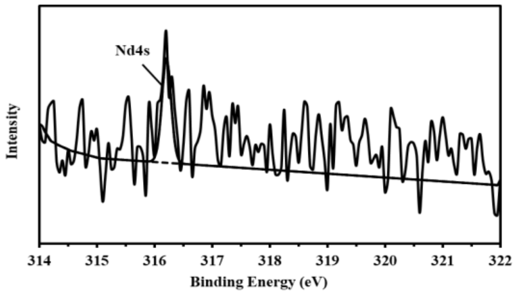
</td><td>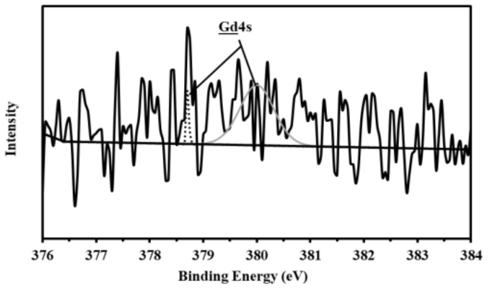
</td></tr>
<tr><td>Nd 3p</td><td>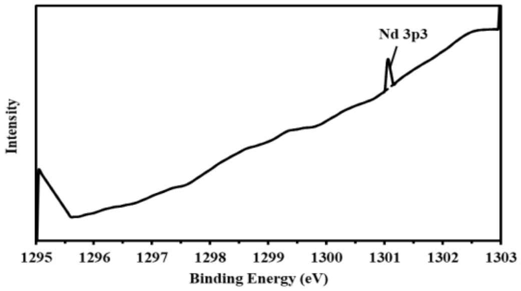
</td><td></td></tr>
<tr><td>Gd 4p</td><td></td><td>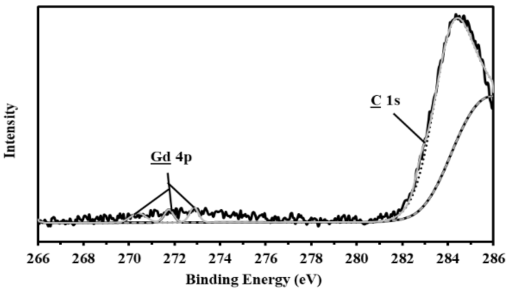
</td></tr>
</table>

Table S8. XPS analysis of signals of mesoporous composite functionalized sorbent before and after sorption of target metal ions (BEs, binding energies and AF, atomic fraction)

<table>
<tr><td rowspan="2">Signal</td><td>NH/SiO_2</td><td>NH/SiO_2+Nd(III)</td><td>NH/SiO_2+Gd(III)</td><td rowspan="2">Assignments</td></tr>
<tr><td>BE (eV) AF (%)</td><td>BE (eV) AF (%)</td><td>BE (eV) AF (%)</td></tr>
<tr><td rowspan="6">C *1s*</td><td>283.75 (9.24)</td><td rowspan="2">284.48 (85.66)</td><td rowspan="2">284.56 (87.97)</td><td>C (C, H)</td></tr>
<tr><td rowspan="5">284.72 (90.76)</td><td>C (N)</td></tr>
<tr><td>285.82 (9.23)</td><td rowspan="2">286.25 (6.83)</td><td>C(-O, =N)</td></tr>
<tr><td rowspan="3">287.01 (4.46)</td><td>C-O-C</td></tr>
<tr><td>287.8 (1.22)</td><td>N-C=O</td></tr>
<tr><td>289.55 (3.98)</td><td>O-C=O</td></tr>
<tr><td rowspan="5">O *1s*</td><td>532.35 (93.27)</td><td>532.47 (91.97)</td><td>532.43 (93.5)</td><td>O(C, H,=C, S) SiO_2</td></tr>
<tr><td>533.95 (5.2)</td><td>534.25 (7.07)</td><td>534.3 (5.87)</td><td>C-C-O</td></tr>
<tr><td>535.3 (1.53)</td><td>535.75 (0.38)</td><td></td><td>Na KLL</td></tr>
<tr><td></td><td>529.3 (0.58)</td><td></td><td>O-Nd</td></tr>
<tr><td></td><td></td><td>529.3 (0.63)</td><td>O-Gd</td></tr>
<tr><td rowspan="3">N *1s*</td><td>398.36 (94.81)</td><td>398.57 (95.33)</td><td>398.6 (93.45)</td><td>N(C,=C, H)</td></tr>
<tr><td>400 (5.19)</td><td>399.95 (1.75)</td><td>399.85 (1.81)</td><td>O=C-NH</td></tr>
<tr><td></td><td>400.65 (2.92)</td><td>401 (4.74)</td><td>N(M)</td></tr>
<tr><td rowspan="3">Si *2p*</td><td>102.94 (97.83)</td><td>103.12 (93.98)</td><td>103.01 (94.81)</td><td>Si $2p^{3+}$</td></tr>
<tr><td>104.5 (2.17)</td><td>104.65 (5.74)</td><td>104.65 (5.19)</td><td>Si $2p^{4+}$</td></tr>
<tr><td></td><td>105.85 (0.28)</td><td></td><td>Envelop</td></tr>
<tr><td rowspan="3">S *2p*</td><td></td><td>165.39 (29.57)
166.5 (70.43)</td><td>165.65 (24.87), 167 (13.13)</td><td>S $2p_{1/2}$</td></tr>
<tr><td></td><td></td><td>164.1 (12.07)</td><td>S $2p_{3/2}$</td></tr>
<tr><td></td><td></td><td>169 (26.21), 168.25 (6.6), 167.85 (17.12)</td><td>SO_4^{2-}</td></tr>
<tr><td>Na *1s*</td><td></td><td>1071.84 (100)</td><td>1072.14 (100)</td><td>O-Na</td></tr>
</table>

Cont' Table S8

Signal	NH/SiO_2+Nd(III) BE (eV) AF (%)	Assignments	NH/SiO_2+Gd(III) BE (eV) AF (%)	Assignments
M3d	980.16 (78.55), 984.05 (5.11)	Nd *3*d$_{5/2}$	1188.17 (76.36)	Gd *3*d$_{5/2}$
	994.7 (1.69), 995.75 (0.95), 989.05 (0.93), 993.3 (0.45), 998.25 (0.47)	Satellite peaks	1214.6 (0.87)	Gd_2O_3 d$_{3/2}$
	1000.85 (11.86)	Nd *3*d$_{7/2}$	1216.3 (1.53)	Satellite peaks
			1219.2 (21.24)	Gd *3*d$_{3/2}$
M *4d*	117.74 (22.6), 118.4 (77.4)	Nd *4*d	143.15 (10.29)	Gd *4*d$_{5/2}$, Gd_2O_3
			148.6 (8.6)	Gd *4*d$_{3/2}$
			151.15 (6.65)	Satellite peaks
			154.02 (73.02)	Si
			158.15 (1.44)	
Gd *4p*			270.4 (4.54), 271.75 (3.12), 272.85 (2.82)	Gd *4*p
			284.56 (89.52)	C 1s
M *4s*	316.21 (100)	Nd *4*s	378.72 (9.18), 380 (90.82)	Gd *4*s
Nd *3*p3	1301.07 (100)	Nd *3*p3		

Table S9. Semi-quantitative EDX analysis of mesoporous composite functionalized sorbent before and after loading with Nd(III).

<table>
<tr><td>Material</td><td>SEM micrograph</td><td>EDX semi-quantitative analysis</td></tr>
<tr><td>Sorbent</td><td>

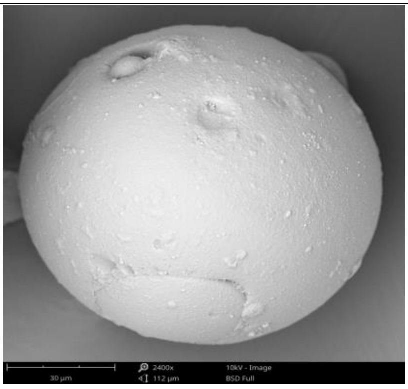

</td><td>

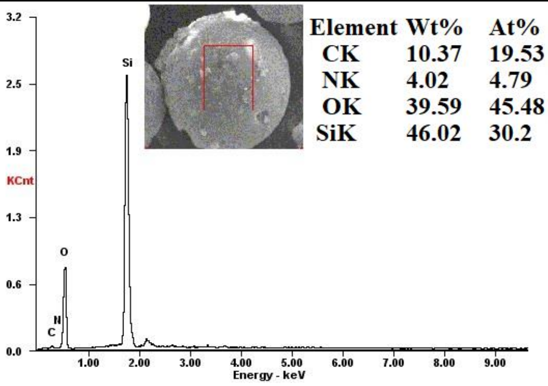

</td></tr>
<tr><td>Sorbent
+ Nd(III)</td><td>

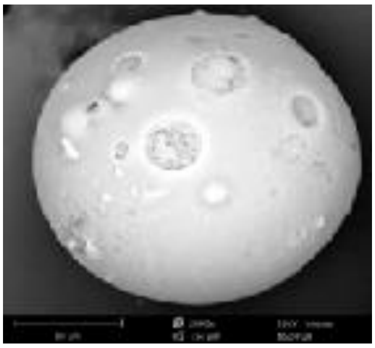

</td><td>

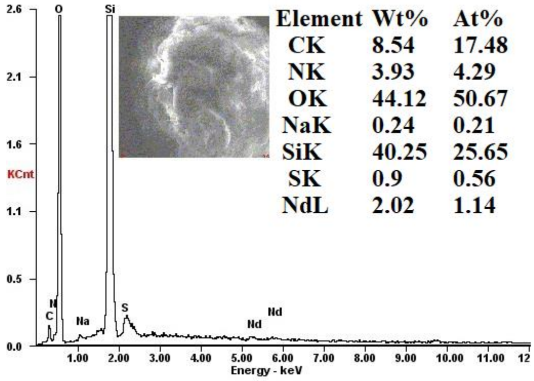

</td></tr>
<tr><td>Sorbent
after elution</td><td>

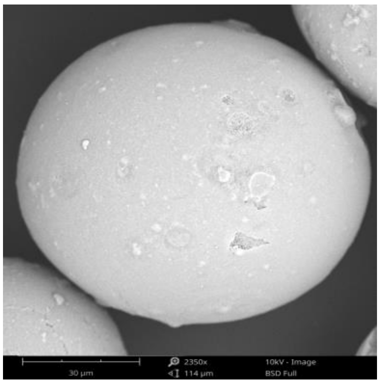

</td><td>

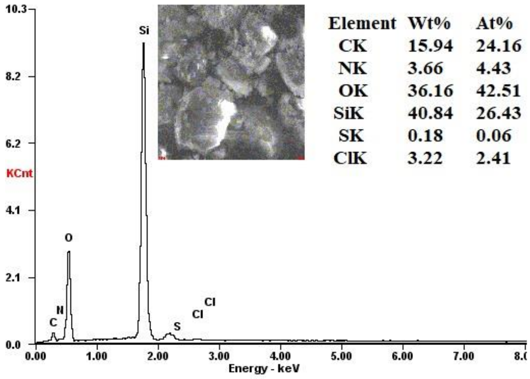

</td></tr>
</table>

Table S10. Semi-quantitative EDX analysis of mesoporous composite functionalized sorbent before and after loading with Gd(III).

<table>
<tr><th>Material</th><th>SEM micrograph</th><th>EDX semi-quantitative analysis</th></tr>
<tr><td>Sorbent</td><td>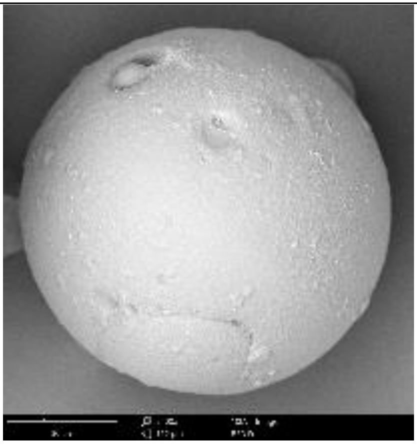</td><td>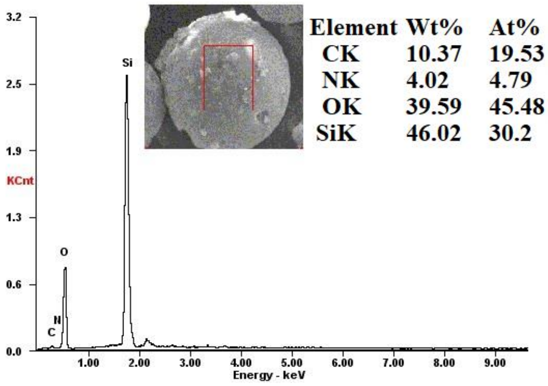
</td></tr>
<tr><td>Sorbent
+ Gd(III)</td><td>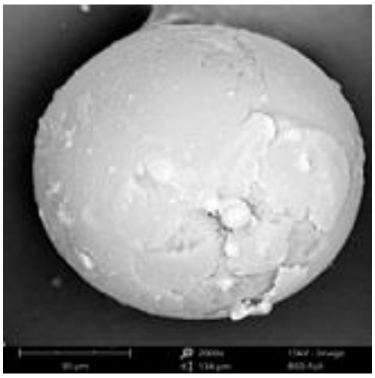</td><td>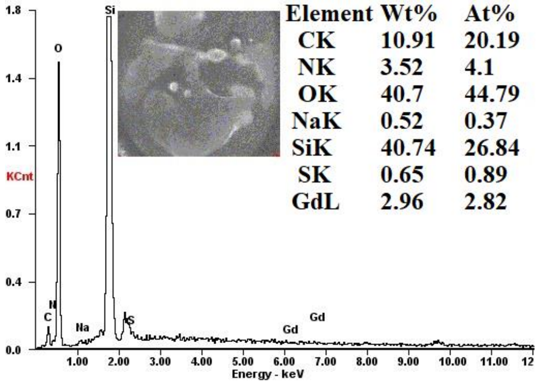
</td></tr>
<tr><td>Sorbent
after elution</td><td>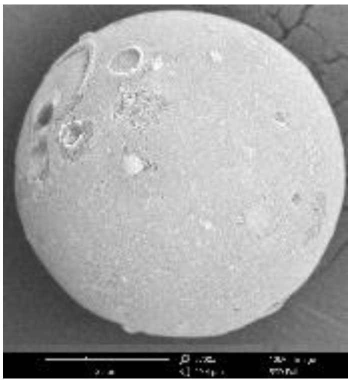</td><td>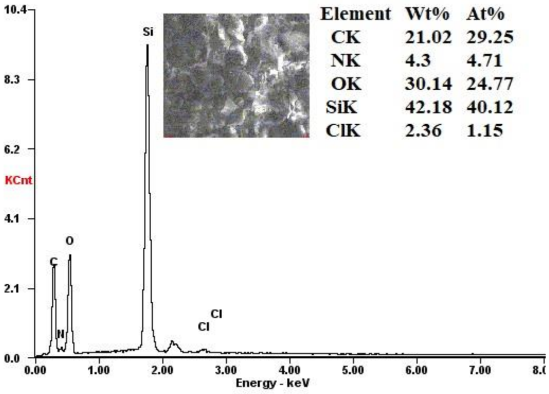
</td></tr>
</table>

Table S11. Solution-phase properties for selected metal ions – Competitive sorption from multi-component equimolar solutions.

Aqua complex	M-O distance	Shannon radius (Å)	Configuration	Coordination number	Electronegativity	pK_a	pK_s	$-\Delta G^{\circ}_{hydra}$ (kcal/mol)
$Nd(H_2O)_9^{2+}$	2.49	1.163	Tricapped trigonal prism	9	3.085	8	25.2	783.9
$Gd(H_2O)_9^{2+}$	2.415	1.053	Tricapped trigonal prism	8	3.111	-	24.5	806.6
$Sc(H_2O)_8^{3+}$	2.17+2.33	0.87	Tricapped trigonal prism	8	3.133	4.8	29.4	907
$Ca(H_2O)_8^{2+}$	2.46	1.12	Square antiprism	8	1.862	12.7	5.06	359.7
$Mg(H_2O)_6^{2+}$	2.10	0.72	Octaehedron	6	2.158	11.42	9.2	437.4

Data from Li et al. [23]

Table S12. Sorption properties for Nd(III) – Comparison of performances (q_{mL}, mmol Nd g^{-1}; b_L, L $mmol^{-1}$).

Sorbent	pH	Equilibrium time (min)	$q_{m,L}$	b_L	Reference
Ion-imprinted polymer particles	7.7	10	0.24	175	[24]
Sargassum sp.	5	180	0.70	27.77	[25]
Kluyveromyces marxianus	1.5	1440	0.083	5.63	[26]
Phosphorus-based sol-gel sorbent	6	180	1.13	-	[27]
Extractant impregnated magnetic microcapsules	4	600-720	1.04	4904	[28]
Calixarene-functionalized graphene oxide composite	7	240	2.16	3.38	[29]
Cysteine/magnetite NPs	7	30	0.59	261.4	[30]
IL-impregnated silica	3.5	200	0.145	267	[31]
Fumarated polystyrene	5	50	0.30	5.87	[32]
Chlorella vulgaris	5	30	0.87	4.18	[33]
Poly-γ glutamic acid sorbent	3	-	1.64	8.47	[34]
Graphitic-C_3N_4 nanosheets	8	360	0.91	140	[35]
Carboxylic acid modified corn stalk gel	3	360	2.44	591	[36]
Diatomaceous earth	5	150	1.17	26.1	[37]
Lanthanide MOF	6	120	0.99	5.19	[38]
Mesoporous composite functionalized sorbent	5	40	1.06	1.24	This work

Table S13. Sorption properties for Gd(III) – Comparison of performances (q_{mL}, mmol Gd g^{-1}; b_L, L $mmol^{-1}$).

Sorbent	pH	Equilibrium time (min)	$q_{m,L}$	b_L	Reference
Sargassum sp.	5	180	0.67	28.79	[25]
Kaolinite	5	3600	0.0031	17.6	[39]
Tulsion CH-93 resin	a	360	0.072	396.9	[40]
Dowex-HCR S/S resin	4	40	0.42	127.2	[41]
Carbamoylmethyl phosphonated-based polymer	1	1200	0.6	-	[42]
Extractant/microcapsules	7	3600	0.44	1.06	[43]
Cysteine/magnetite NPs	7	30	0.62	127.4	[30]
IL-impregnated silica	3.5	200	0.154	2129	[31]
Functionalized chitosan	5	60	1.48	38.3	[44]
CNT/graphene oxide	5.9	90	3.40	66.0	[45]
Banana peel	5.2	1440	0.294	330	[46]
DTPA-chitosan/magnetite	5	120	10.6	7.55	[47]
Crown-ether grafted polystyrene	5	240	0.0112	13.7	[48] [48]
Functionalized cellulose/magnetite NPs	6	40	0.43	100	[49]
Imprinted mesoporous carboxymethyl chitosan film	7	360	0.16	6.29	[50]
Amino-phosphonic acid functionalized hollow silica nanospheres	5	20	2.56	2.66	[51]
Mesoporous composite functionalized sorbent	5	40	1.41	0.93	This work

a: 0.1 M H_3PO_4 solution.

Table S14. Semi-quantitative EDX analysis of mesoporous composite functionalized sorbent after loading with equimolar Nd, Gd, Sc, Ca and Mg solution and after treatment with polymetallic (REEs) solution.

<table>
<tr><td>Material</td><td>SEM micrograph</td><td>EDX semi-quantitative analysis</td></tr>
<tr><td>Sorbent + Nd(III), Gd(III), Sc(III), Ca(II) and Mg(II) at pH 5</td><td>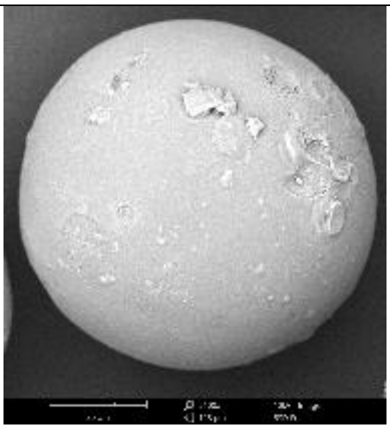</td><td>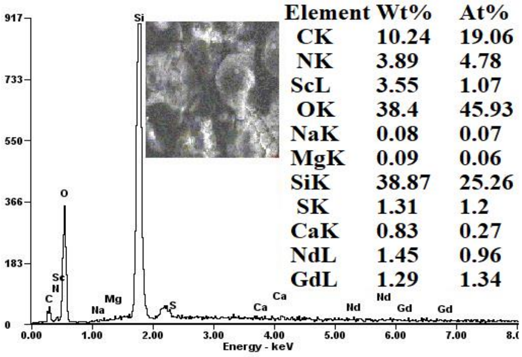
</td></tr>
<tr><td>Sorbent + polymetallic solution (REEs) at pH 5</td><td>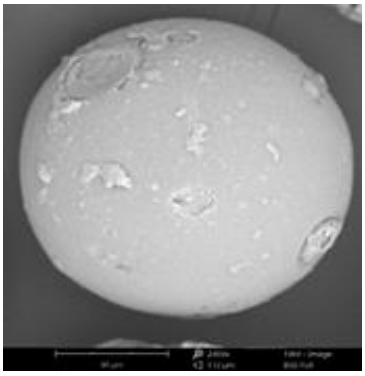</td><td>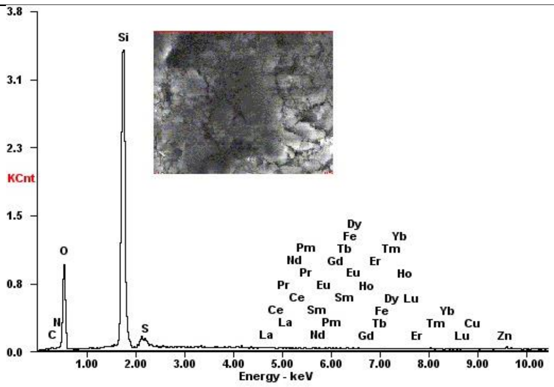
</td></tr>
</table>

Table S15. SEM microphotographs of mesoporous composite functionalized sorbent after 5 cycles of sorption and desorption – Stability

<table>
<tr><th>Loaded metal</th><th>SEM micrograph</th><th>EDX semi-quantitative analysis</th></tr>
<tr><td>Nd(III)</td><td>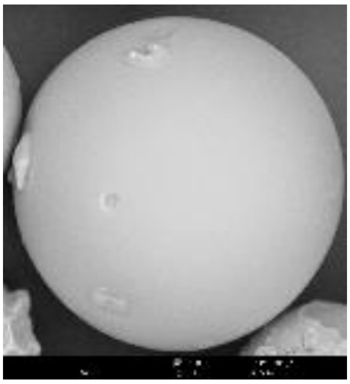</td><td>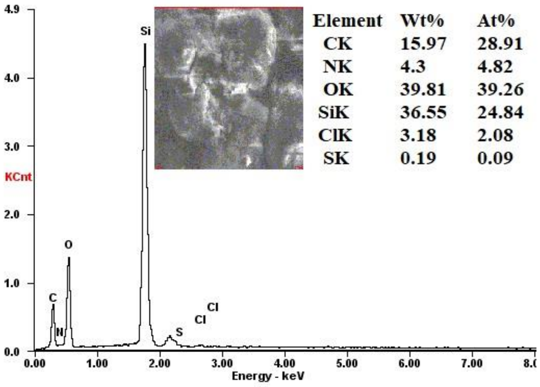
</td></tr>
<tr><td>Gd(III)</td><td>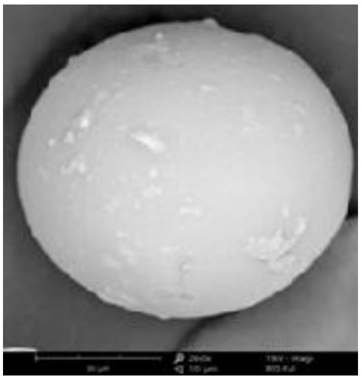</td><td>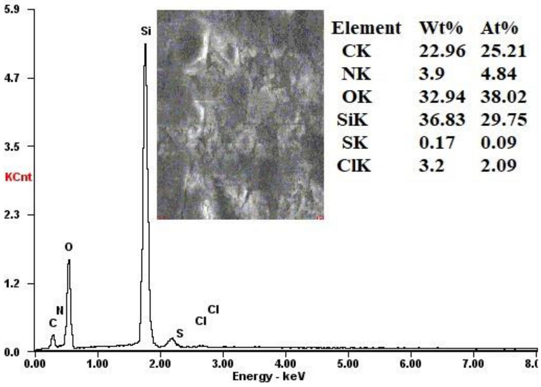
</td></tr>
</table>

Table S16. Semi-quantitative EDX analysis of REEs ore, and after sorption/elution/oxalic acid precipitation.

<table>
<tr><td>Sample</td><td>MEB observation (inset) and EDX semi-quantitative analysis</td></tr>
<tr><td>Unpurified REE (ore)</td><td>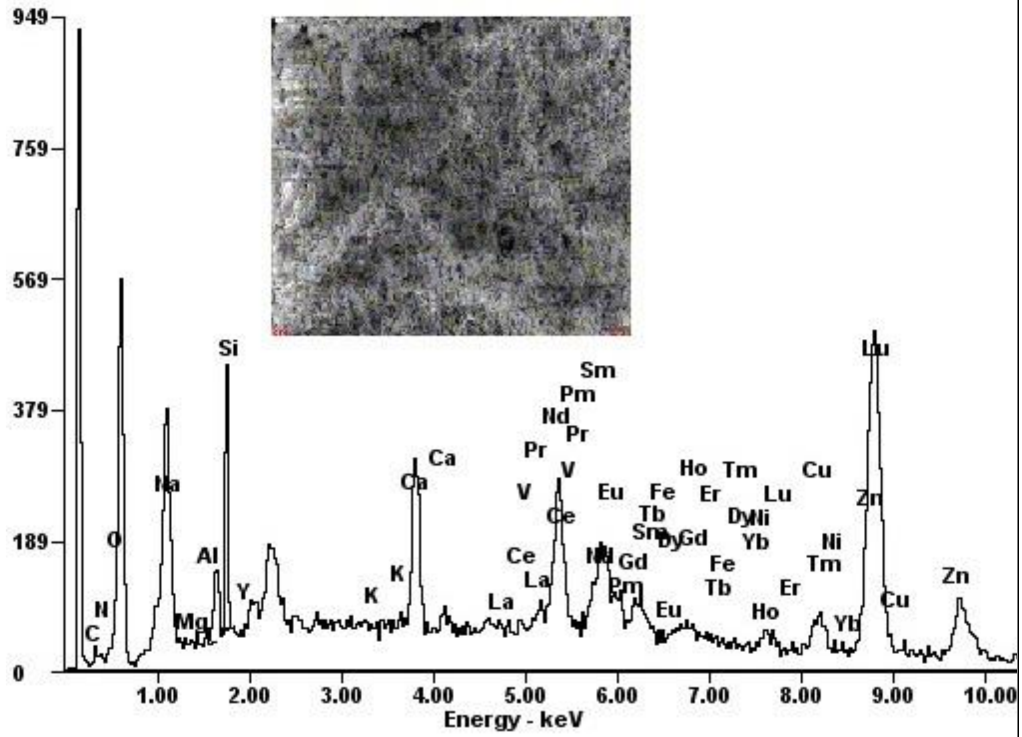
</td></tr>
<tr><td>Purified precipitate of REEs (after leaching, sorption, elution and oxalic acid precipitation)</td><td>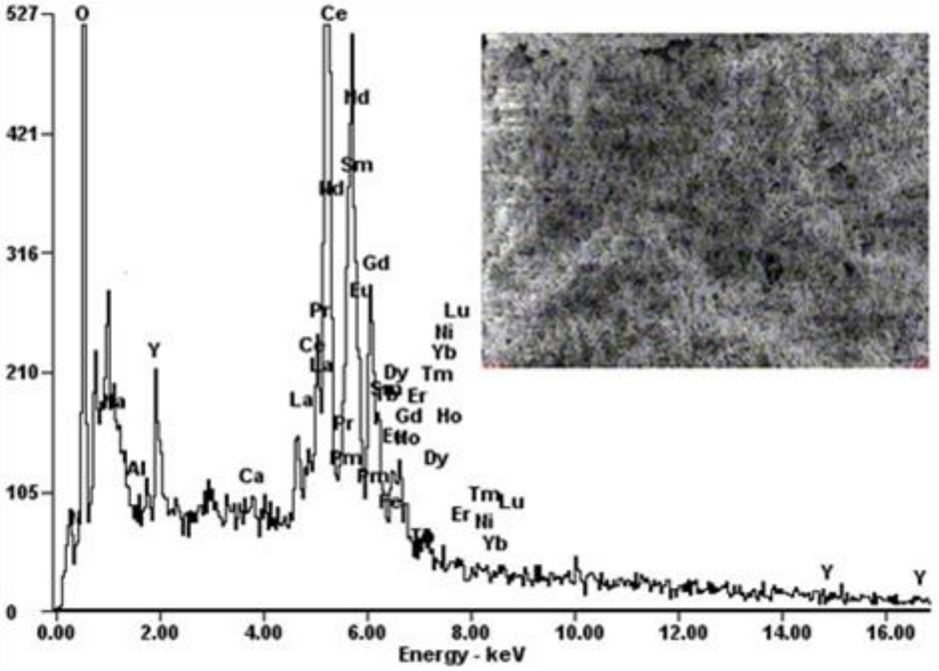
</td></tr>
</table>

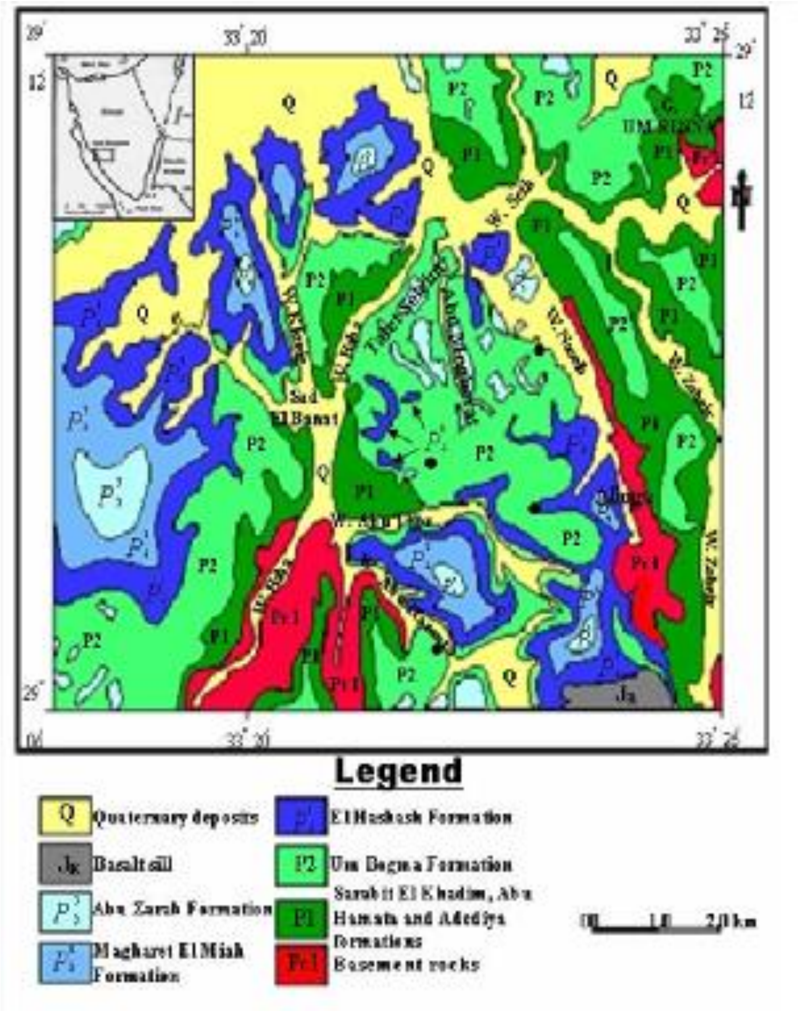

Figure S1. Geological map for ore sampling (gibbsite materials from Abu Mogherat site in South Western Sinai, Egypt).

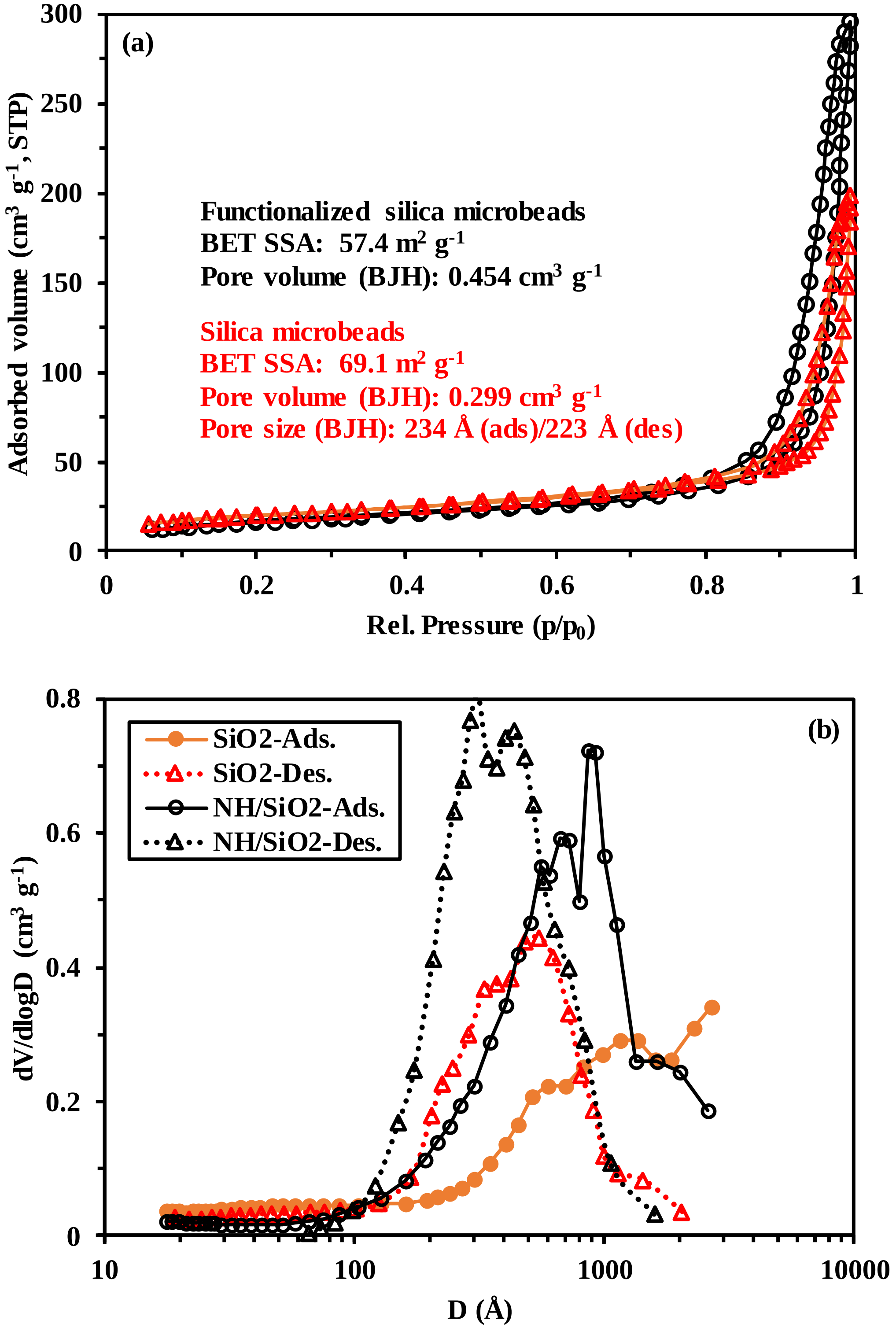

Figure S2. Textural analysis of mesoporous silica microbeads and mesoporous composite functionalized sorbent (NH/SiO_2): (a) nitrogen adsorption/desorption isotherms), (b) pore size distribution (BJH method with Harkins-Jura thickness equation and Faas correction) for adsorption (ads.) and desorption (des.) isotherms.

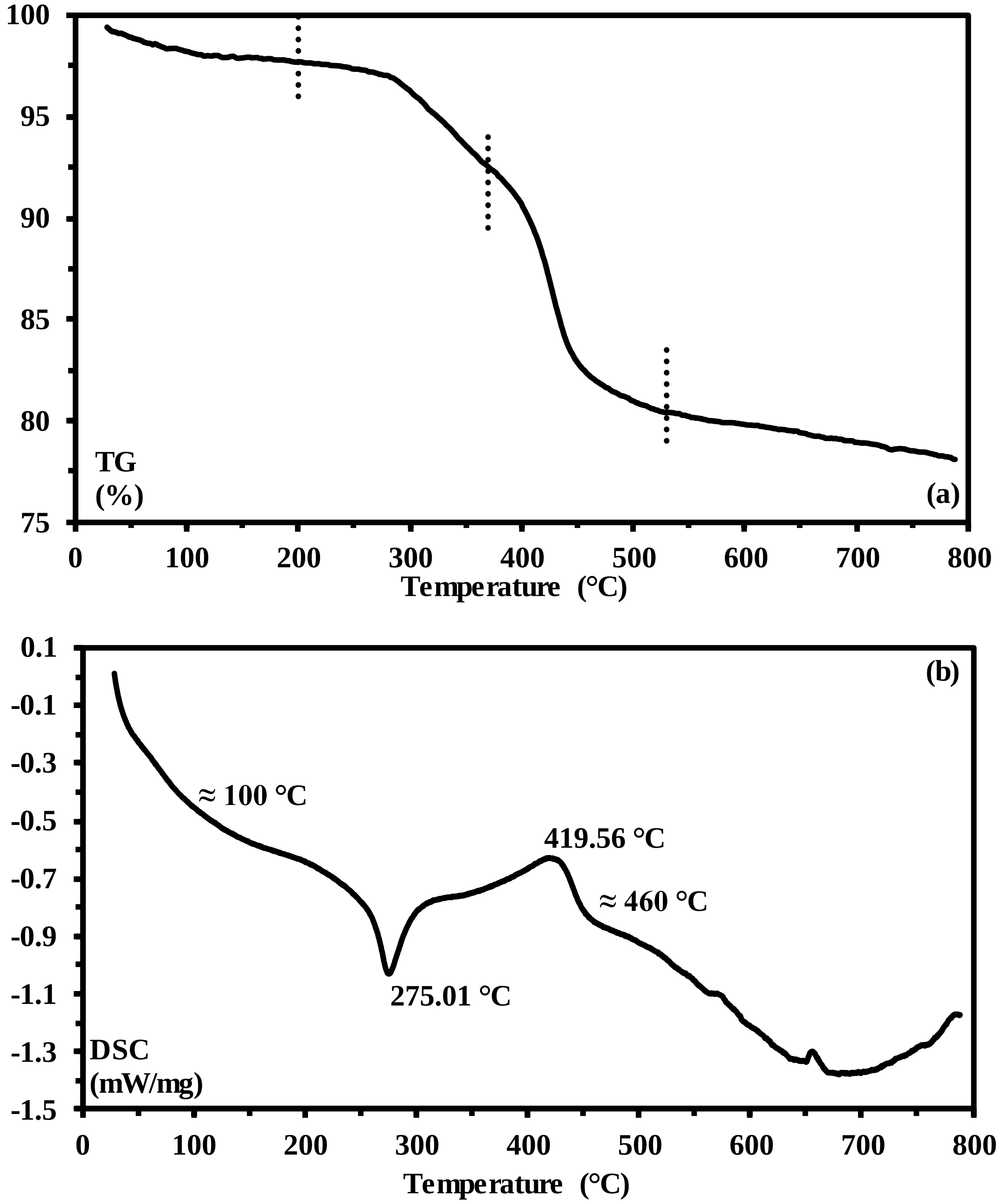

Figure S3. Thermogravimetric analysis of mesoporous composite functionalized sorbent: TGA (a), DSC (b).

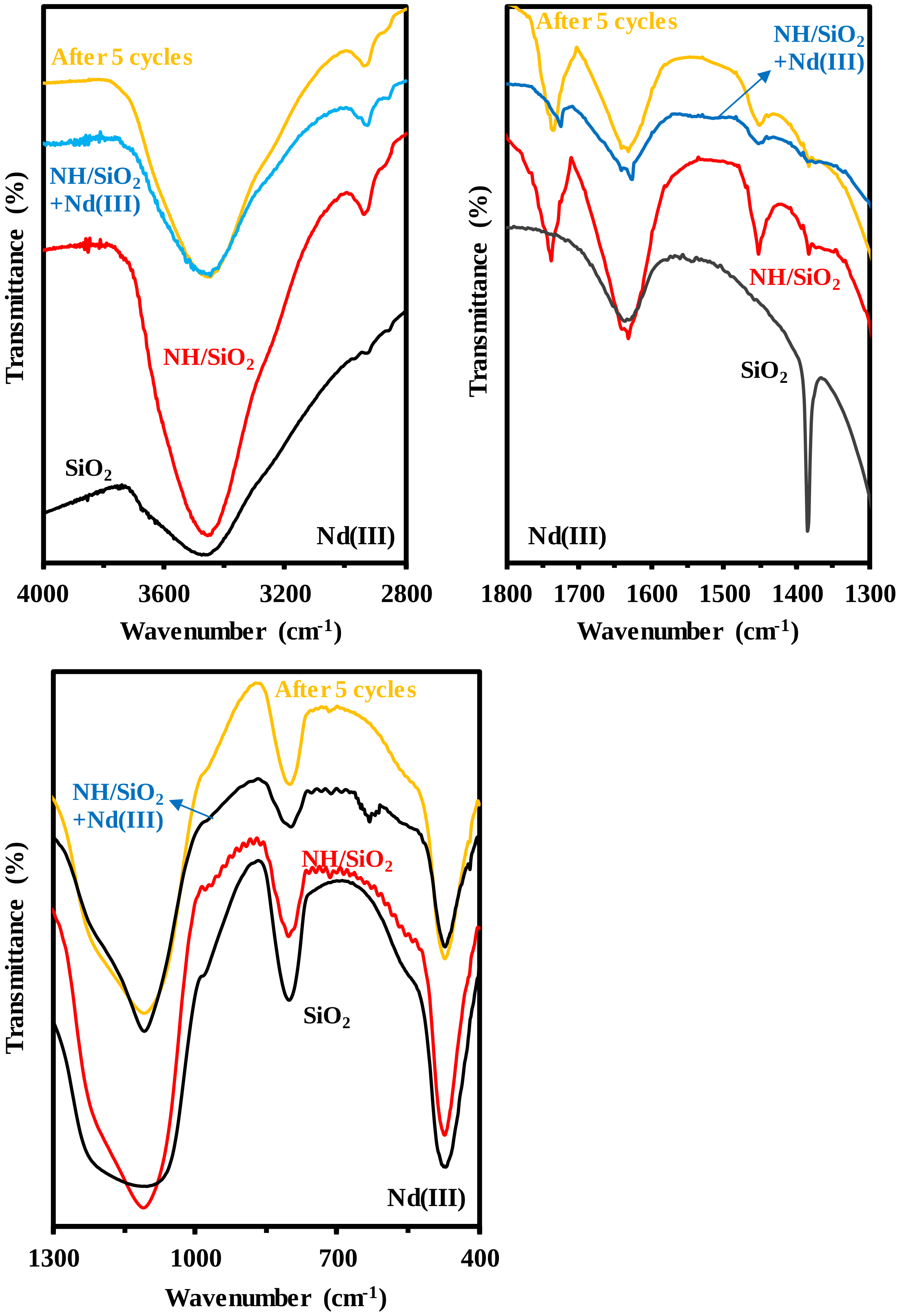

Figure S4. FTIR spectra of mesoporous SiO_2, mesoporous composite functionalized sorbent, before and after Nd(III) sorption, and after 5 cycles of sorption and desorption.

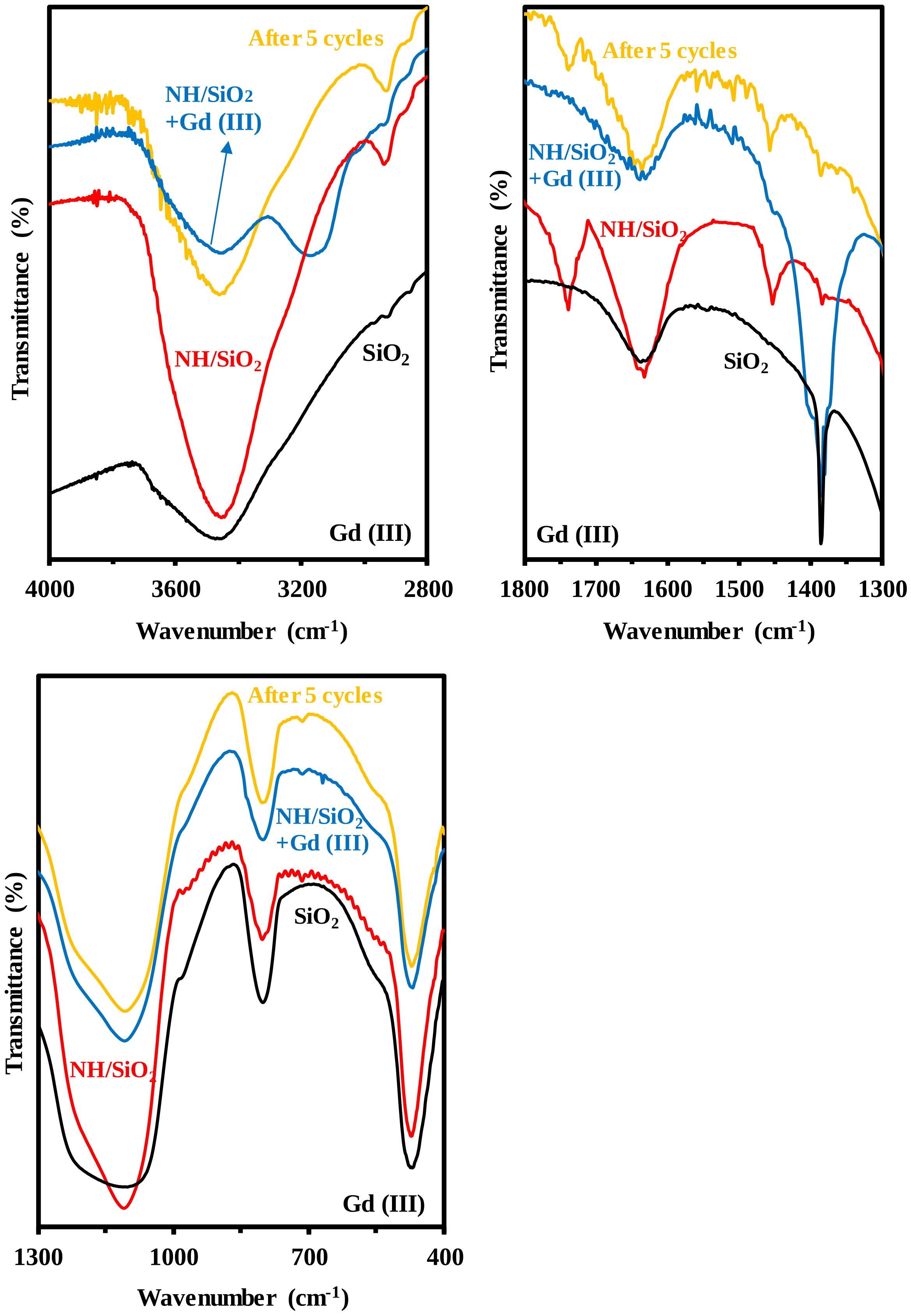

Figure S5. FTIR spectra of mesoporous SiO_2, mesoporous composite functionalized sorbent, before and after Gd(III) sorption, and after 5 cycles of sorption and desorption.

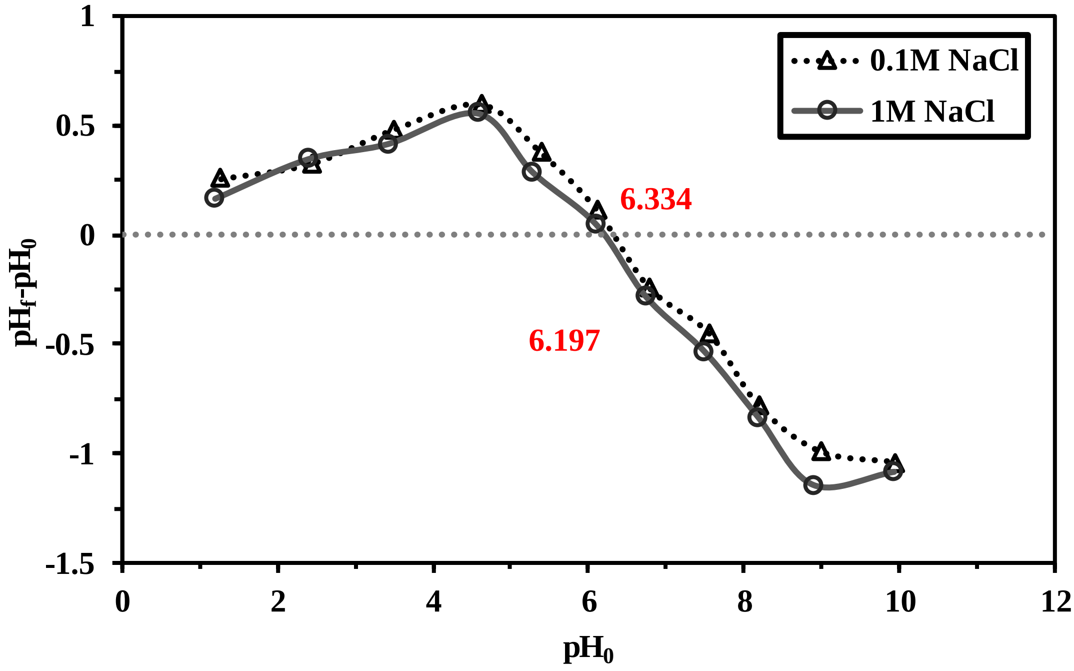

Figure S6. Acid-base properties – pH_{PZC} determined by the pH-drift method (Sorbent dosage, SD: 2 g L^{-1}; contact time: 48 h; T: 22 ±2 °C; background salt: NaCl solution at 0.1 and 1 M concentrations).

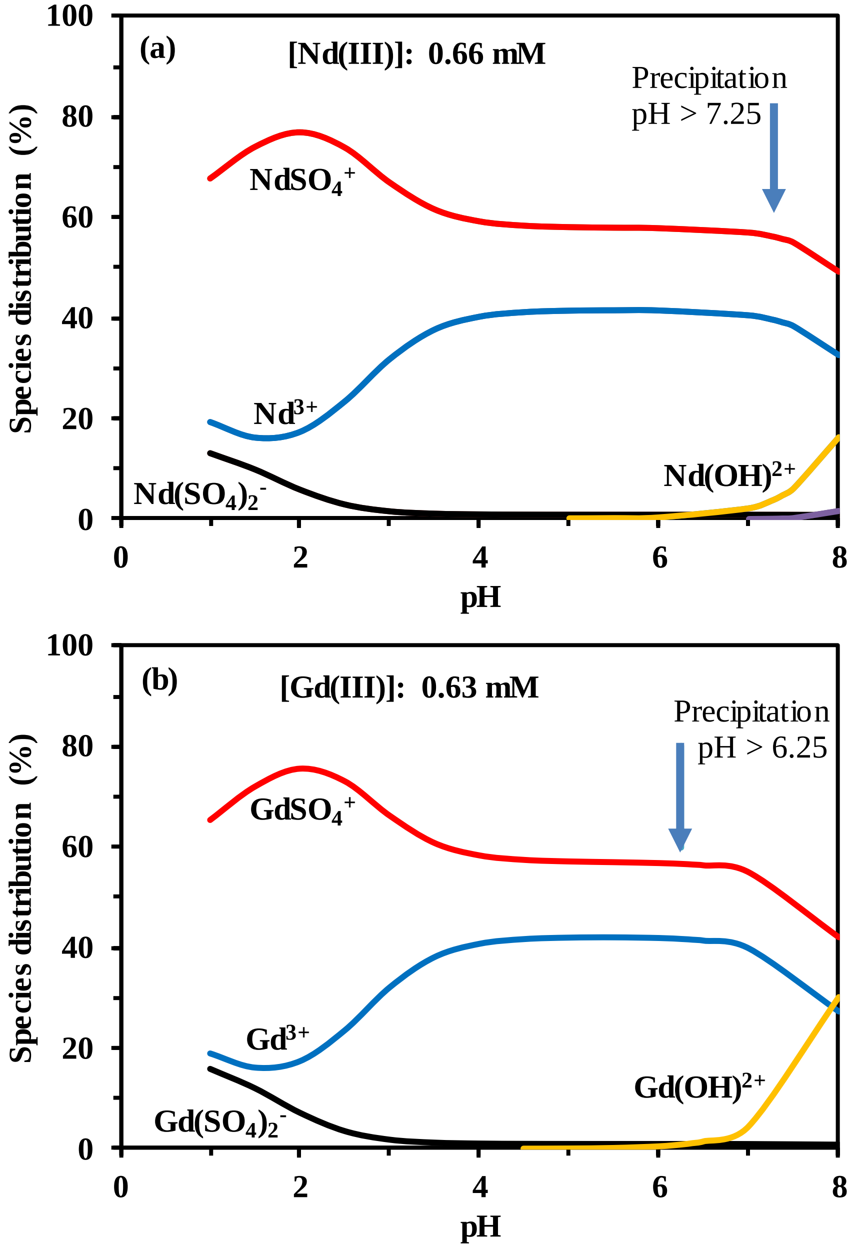

Figure S7. Speciation diagrams for Nd(III) and Gd(III) under the experimental conditions selected for the study of pH effect (C_0: 0.66 mmol Nd L^{-1} or 0.63 mmol Gd L^{-1}; sulfate salts, pH controlled with sulfuric acid and sodium hydroxide).

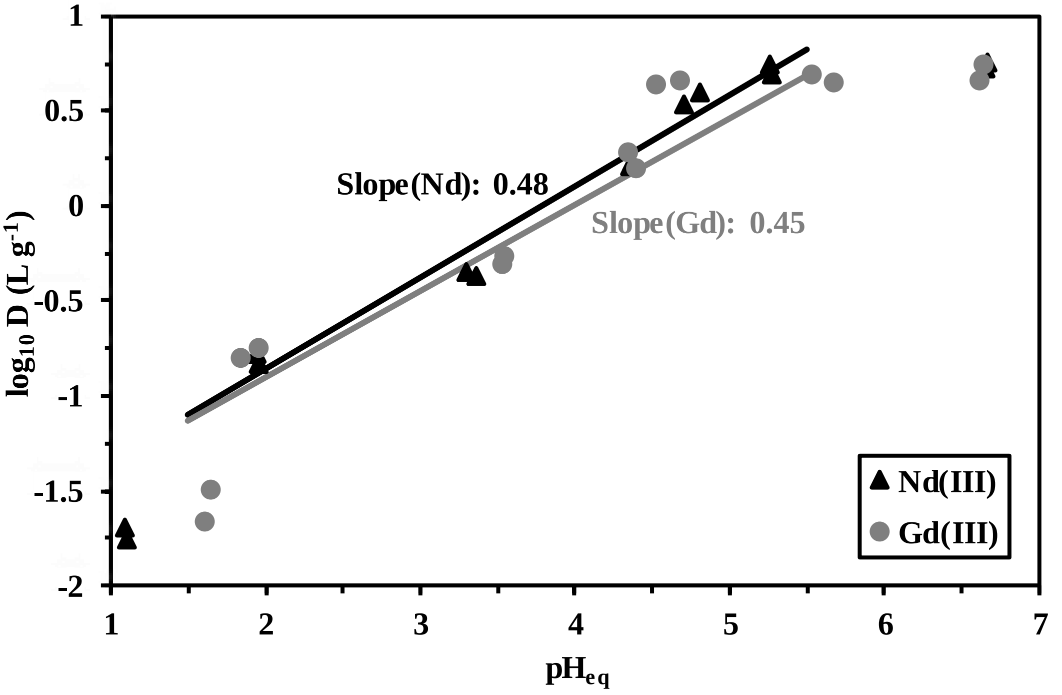

Figure S8. Effect of equilibrium pH on the distribution ratio (D, in log_{10} unit) (C_0: 100 mg L^{-1}; 0.66 mmol Nd L^{-1} or 0.63 mmol Gd L^{-1}; SD: 1.42 g L^{-1}; Contact time: 48 h; agitation speed: 170 rpm; T: 22 ±2 °C).

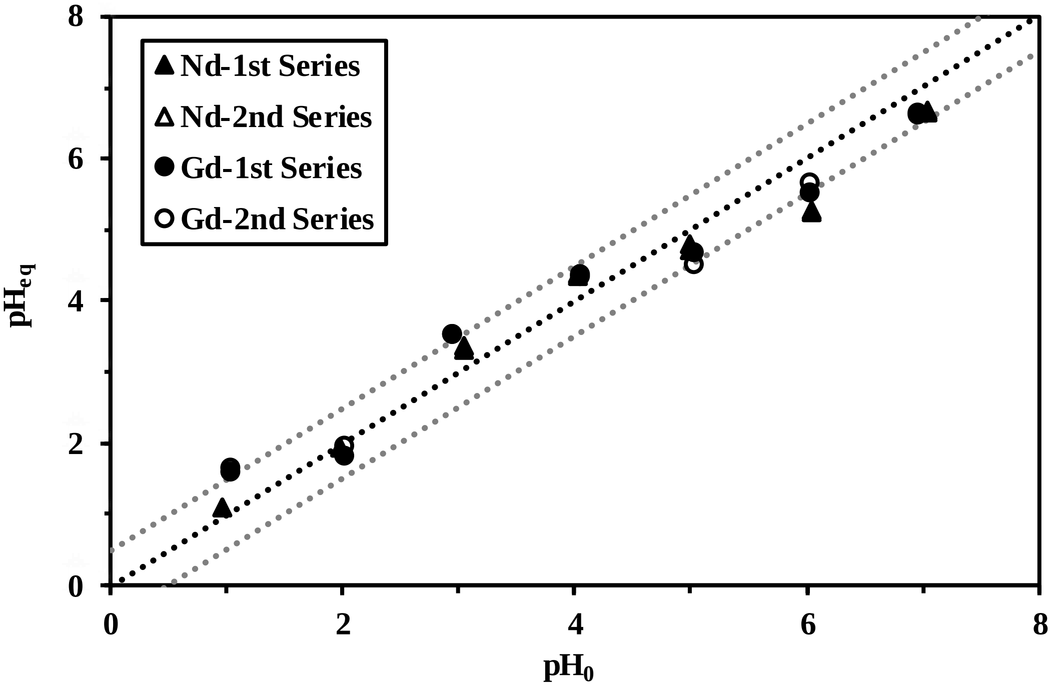

Figure S9. pH variation during metal sorption (C_0: 100 mg L^{-1}; 0.66 mmol Nd L^{-1} or 0.63 mmol Gd L^{-1}; SD: 1.42 g L^{-1}; Contact time: 48 h; agitation speed: 170 rpm; T: 22 ±2 °C).

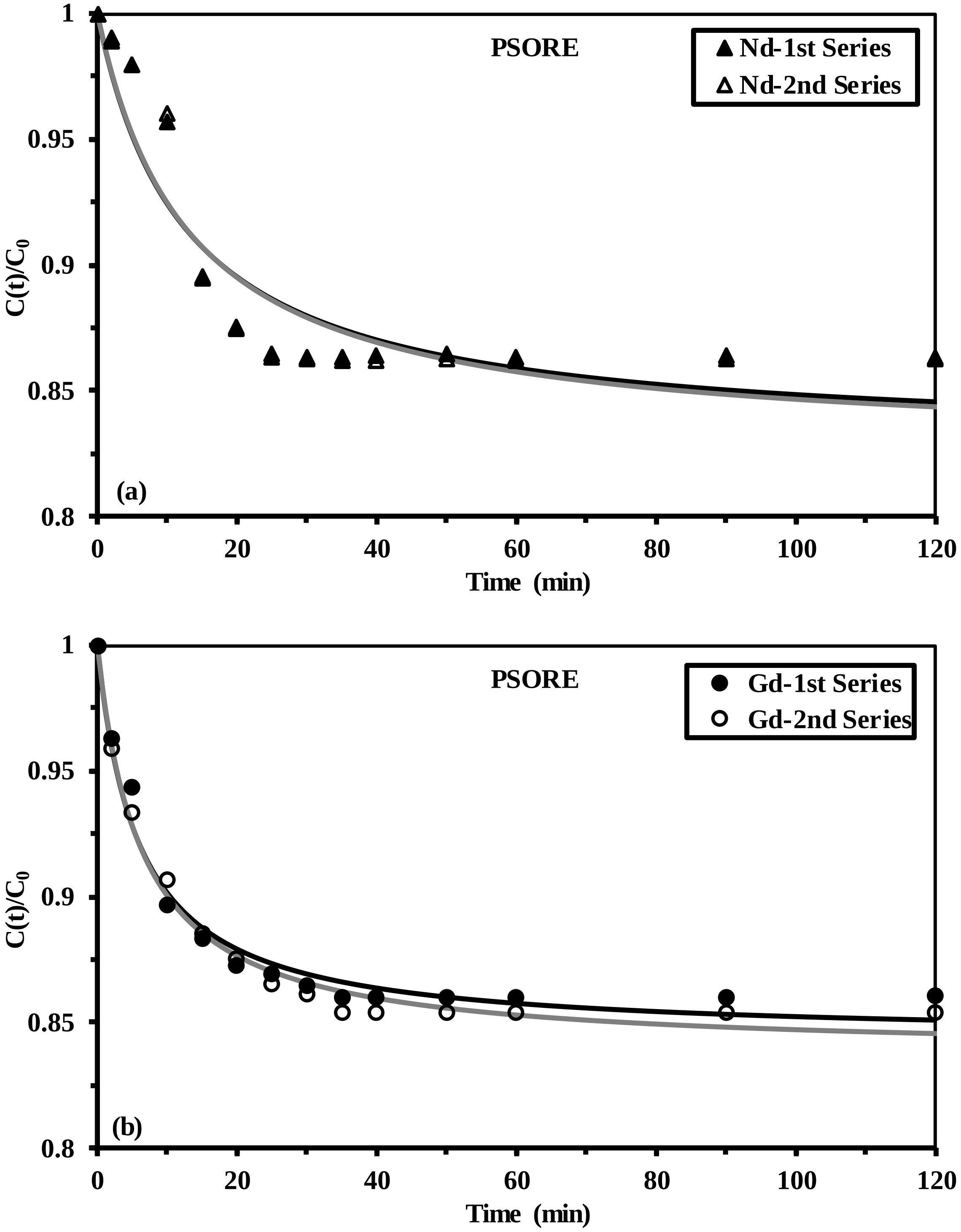

Figure S10. Nd(III) and Gd(III) uptake kinetics using mesoporous composite functionalized sorbent - Modeling with the pseudo-second order rate equation (C_0 : 0.65-0.71 mmol metal L^{-1}; pH_0: 5; pH_{eq}: 4.69-4.76; SD: 0.25 g L^{-1}; agitation speed: 170 rpm; T: 22 ±2 °C).

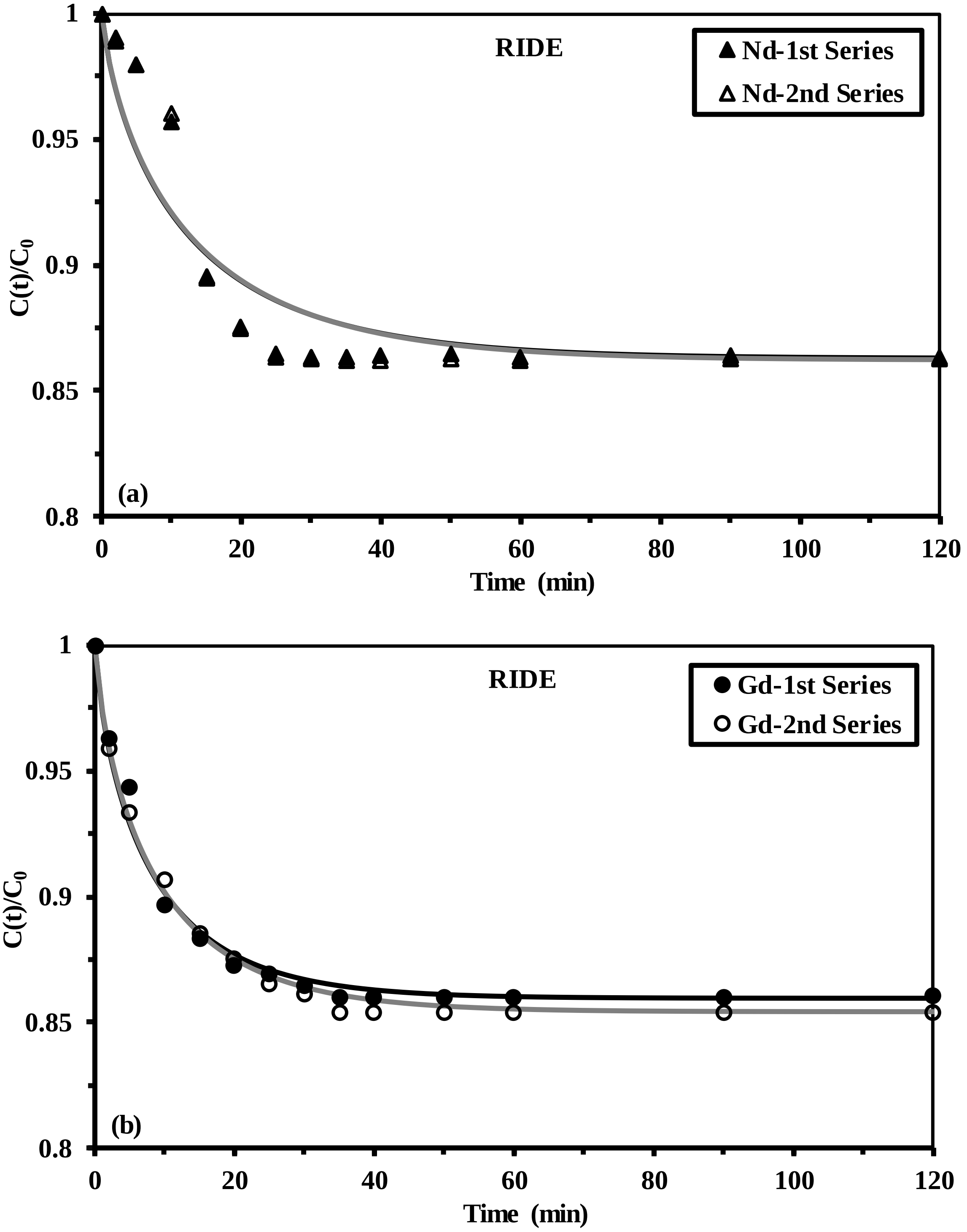

Figure S11. Nd(III) and Gd(III) uptake kinetics using mesoporous composite functionalized sorbent - Modeling with the Crank equation, resistance to intraparticle diffusion model (C_0 : 0.65-0.71 mmol metal L^{-1}; pH_0: 5 ; pH_{eq}: 4.69-4.76; SD: 0.25 g L^{-1}; agitation speed: 170 rpm; T: 22 ±2 °C).

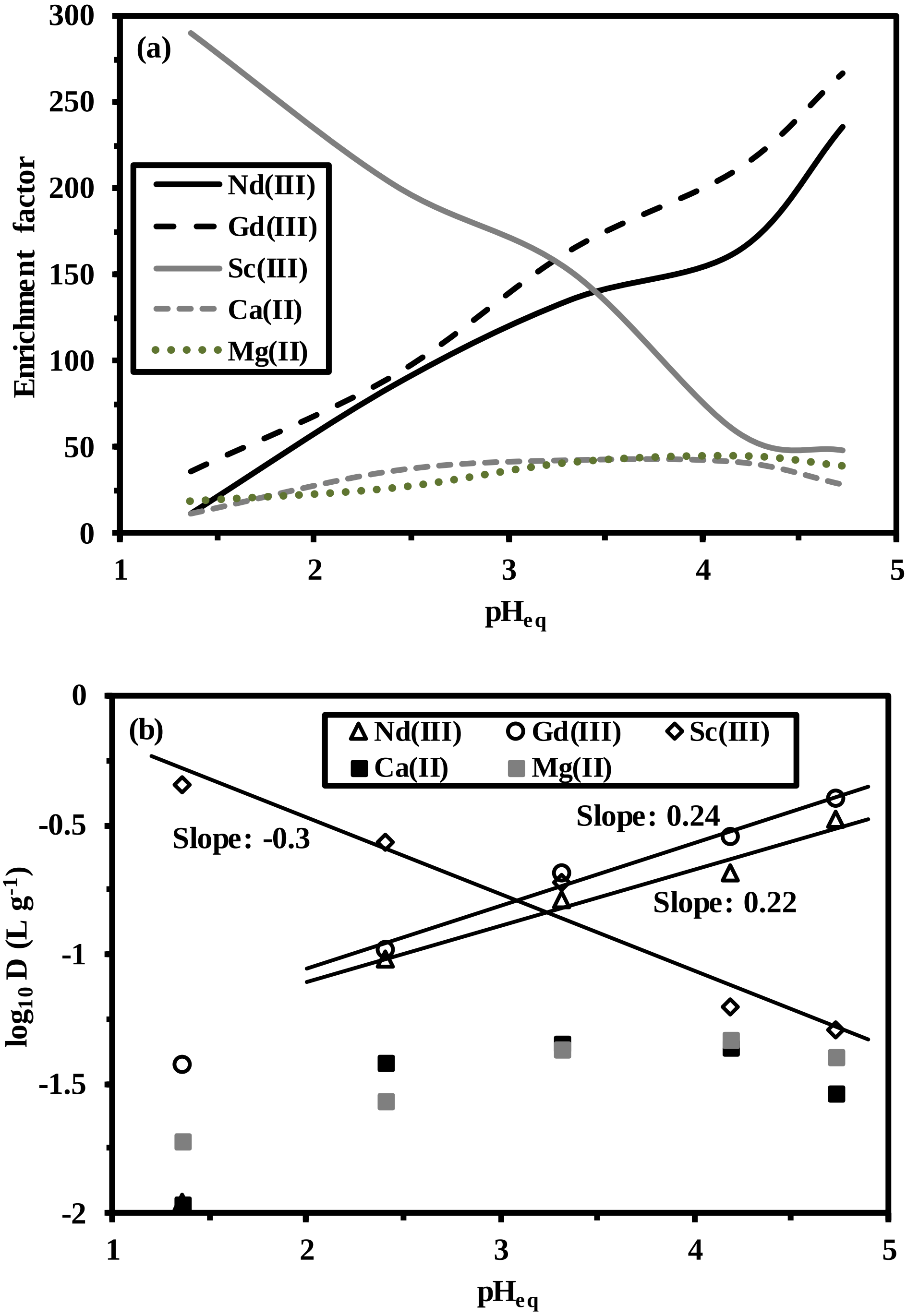

Figure S12. Effect of pH on (a) the enrichment factor, and the distribution ratio, D (EF, L kg^{-1}, EF= q (mmol kg^{-1}) / C_0 (mmol L^{-1})) of metal ions on the mesoporous composite functionalized sorbent compared to their initial concentration in the solution; D, L g^{-1} = q_{eq}/C_{eq}) (equimolar multi-component solutions; C_0: 1 mmol metal L^{-1}; pH_0: 1-5; pH_{eq}: 1.36-4.73; SD: 0.125 g L^{-1}; contact time: 48 h; agitation speed: 170 rpm; T: 22 ±2 °C).

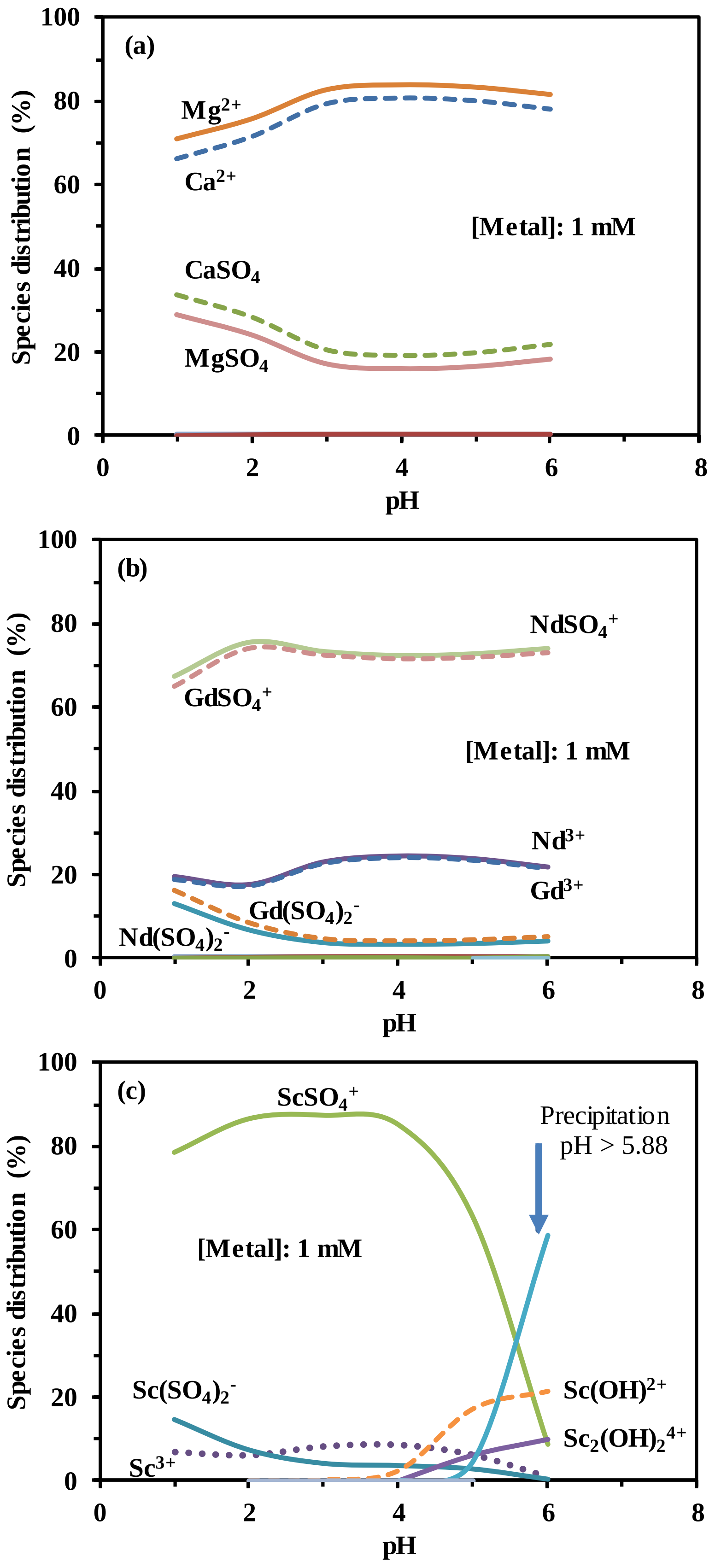

Figure S13. Speciation diagram for multicomponent equimolar solutions (experimental conditions corresponding to Figure 6 (C_0: 1 mmol metal L^{-1}; metal salts and type of acid and base used for pH control taken into account for speciation calculation).

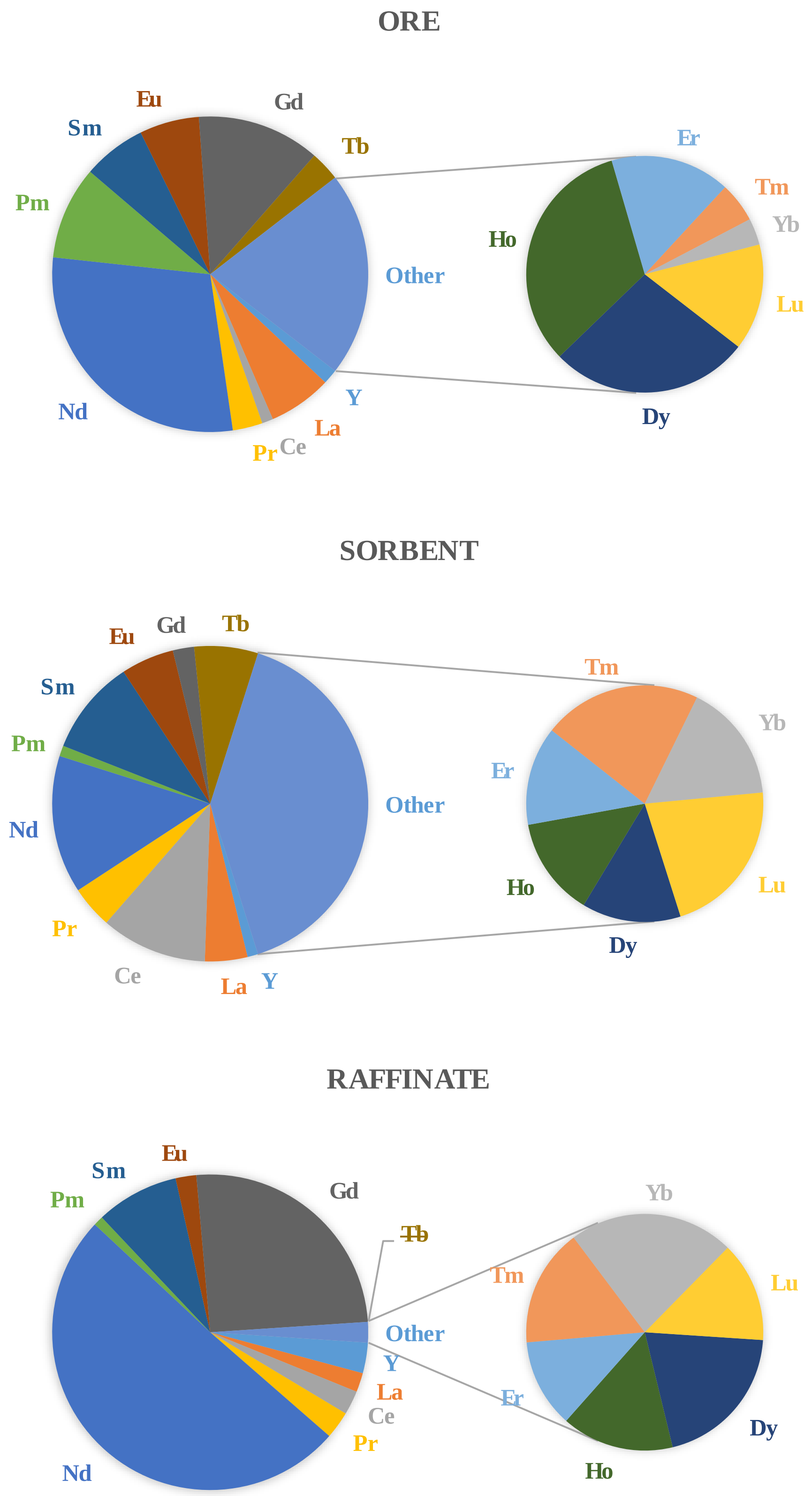

Figure S14. Atomic fractions (semi-quantitative EDX analysis) of REEs in unpurified ore, mesoporous composite functionalized sorbent and raffinate (acid elution (sorbent)/oxalic acid precipitation/calcination).

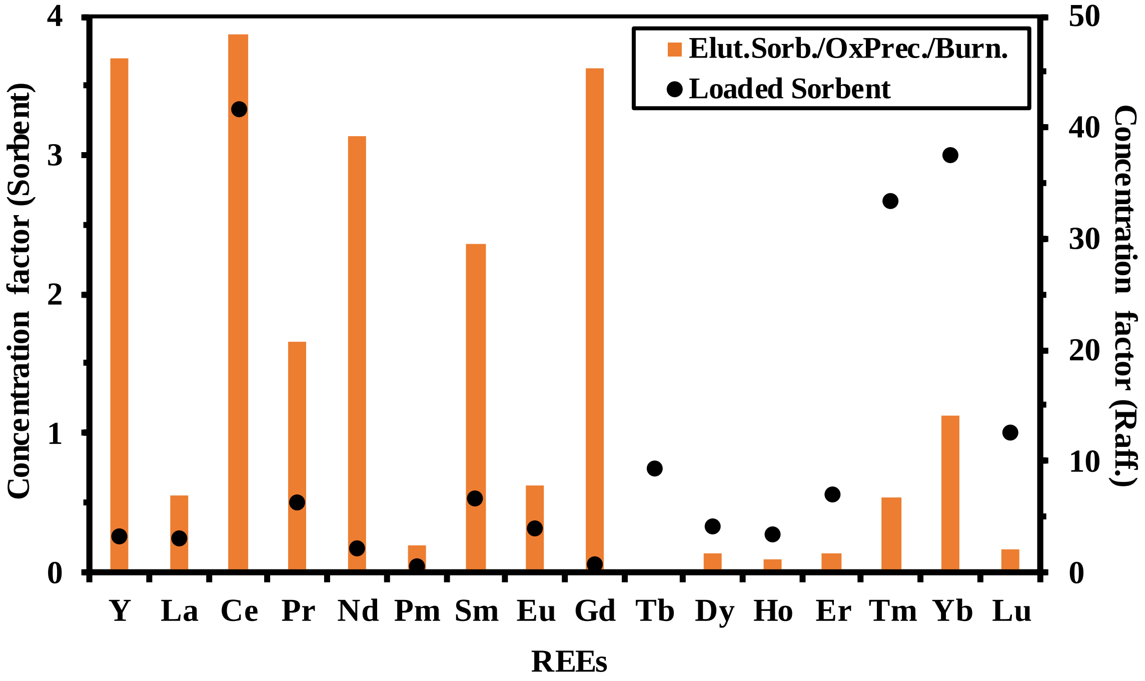

Figure S15. Concentration factor (against atomic fraction of REEs in the ore) in the loaded sorbent and in the raffinate.

References

1. Ho, Y.S.; McKay, G. Pseudo-second order model for sorption processes. *Process Biochem.* **1999**, *34*, 451-465, doi:10.1016/S0032-9592(98)00112-5.
2. Crank, J. *The Mathematics of Diffusion*, 2nd. ed.; Oxford University Press: Oxford, U.K., 1975; pp. 414.
3. Falyouna, O.; Eljamal, O.; Maamoun, I.; Tahara, A.; Sugihara, Y. Magnetic zeolite synthesis for efficient removal of cesium in a lab-scale continuous treatment system. *J. Colloid Interface Sci.* **2020**, *571*, 66-79, doi:10.1016/j.jcis.2020.03.028.
4. Tien, C. *Adsorption Calculations and Modeling*; Butterworth-Heinemann: Newton, MA, 1994; pp. 243.
5. Foo, K.Y.; Hameed, B.H. Insights into the modeling of adsorption isotherm systems. *Chem. Eng. J.* **2010**, *156*, 2-10, doi:10.1016/j.cej.2009.09.013.
6. Shapiro, L. *Rapid analysis of silicate, carbonate, and phosphate rocks*; U.S. Geol. Surv. Bull., Vol. 76, pIII, Report Number 1401: 1975; p Pp. 88.
7. Marczenko , Z. *Spectrophotometric determination of elements*; Ellis Horwood: Chichester (U.K.), 1976; pp. 643.
8. Davies, W.; Gray, W. A rapid and specific titrimetric method for the precise determination of uranium using iron(II) sulphate as reductant. *Talanta* **1964**, *11*, 1203-1211, doi:http://dx.doi.org/10.1016/0039-9140(64)80171-5.
9. Hamza, M.F.; Abdel-Rahman, A.A.H. Extraction studies of some hazardous metal ions using magnetic peptide resins. *J. Dispersion Sci. Technol.* **2015**, *36*, 411-422, doi:10.1080/01932691.2014.905955.
10. Hamza, M.F.; Aly, M.M.; Abdel-Rahman, A.A.H.; Ramadan, S.; Raslan, H.; Wang, S.; Vincent, T.; Guibal, E. Functionalization of magnetic chitosan particles for the sorption of U(VI), Cu(II) and Zn(II)—Hydrazide derivative of glycine-grafted chitosan. *Materials* **2017**, *10*, 539-560, doi:doi:10.3390/ma10050539.
11. Coates, J. Interpretation of Infrared Spectra, A Practical Approach. In *Encyclopedia of Analytical Chemistry*, Meyers, R.A., Ed. John Wiley & Sons Ltd: Chichester, U.K., 2000; pp. 10815-10837.
12. Coates, J. Interpretation of Infrared Spectra, A Practical Approach. In *Encyclopedia of Analytical Chemistry*, John Wiley & Sons, Ltd.: 2006; 10.1002/9780470027318.a5606pp. 1-23.
13. Mohammadi, N.; Ganesan, A.; Chantler, C.T.; Wang, F. Differentiation of ferrocene D-5d and D-5h conformers using IR spectroscopy. *J. Organomet. Chem.* **2012**, *713*, 51-59, doi:10.1016/j.jorganchem.2012.04.009.
14. Colthup, N.B.; Daly, L.H.; Wiberley, S.E. *Introduction to Infrared and Raman Spectroscopy*, 3rd. ed. ed.; Academic Press, Inc.: San Diego, CA (USA), 1990; pp. 560.
15. Hu, X.J.; Wang, J.S.; Liu, Y.G.; Li, X.; Zeng, G.M.; Bao, Z.L.; Zeng, X.X.; Chen, A.W.; Long, F. Adsorption of chromium (VI) by ethylenediamine-modified cross-linked magnetic chitosan resin: Isotherms, kinetics and thermodynamics. *J. Hazard. Mater.* **2011**, *185*, 306-314, doi:10.1016/j.jhazmat.2010.09.034.
16. Liu, N.; Assink, R.A.; Smarsly, B.; Brinker, C.J. Synthesis and characterization of highly ordered functional mesoporous silica thin films with positively chargeable -NH_2 groups. *Chem. Commun.* **2003**, *9*, 1146-1147.
17. Lu, S.; Chen, L.; Hamza, M.F.; He, C.; Wang, X.; Wei, Y.; Guibal, E. Amidoxime functionalization of a poly(acrylonitrile)/silica composite for the sorption of Ga(III) – Application to the treatment of Bayer liquor. *Chem. Eng. J.* **2019**, *368*, 459-473, doi:https://doi.org/10.1016/j.cej.2019.02.094.

18. Zhang, X.; Guan, R.F.; Wu, D.Q.; Chan, K.Y. Enzyme immobilization on amino-functionalized mesostructured cellular foam surfaces, characterization and catalytic properties. *J. Mol. Catal. B: Enzym* **2005**, *33*, 43-50.
19. Zhao, Z.; Xie, X.; Wang, Z.; Tao, Y.; Niu, X.; Huang, X.; Liu, L.; Li, Z. Immobilization of *Lactobacillus rhamnosus* in mesoporous silica-based material: An efficiency continuous cell-recycle fermentation system for lactic acid production. *J. Biosci. Bioeng* **2016**, *121*, 645-651.
20. Namdeo, M.; Bajpai, S.K. Chitosan-magnetite nanocomposites (CMNs) as magnetic carrier particles for removal of Fe(III) from aqueous solutions. *Colloids Surf., A* **2008**, *320*, 161-168, doi:10.1016/j.colsurfa.2008.01.053.
21. Oshita, K.; Takayanagi, T.; Oshima, M.; Motomizu, S. Adsorption behavior of cationic and anionic species on chitosan resins possessing amino acid moieties. *Anal. Sci.* **2007**, *23*, 1431-1434, doi:10.2116/analsci.23.1431.
22. Zhang, X.; Jiao, C.; Wang, J.; Liu, Q.; Li, R.; Yang, P.; Zhang, M. Removal of uranium(VI) from aqueous solutions by magnetic Schiff base: Kinetic and thermodynamic investigation. *Chem. Eng. J.* **2012**, *198*, 412-419, doi:10.1016/j.cej.2012.05.090.
23. Li, K.; Li, M.; Xue, D. Solution-phase electronegativity scale: Insight into the chemical behaviors of metal ions in solution. *J. Phys. Chem. A* **2012**, *116*, 4192-4198, doi:10.1021/jp300603f.
24. Krishna, P.G.; Gladis, J.M.; Rao, T.P.; Naidu, G.R. Selective recognition of neodymium(III) using ion imprinted polymer particles. *J. Mol. Recognit.* **2005**, *18*, 109-116, doi:10.1002/jmr.720.
25. Oliveira, R.C.; Garcia, O., Jr. Study of biosorption of rare earth metals (La, Nd, Eu, Gd) by *Sargassum* sp biomass in batch systems: physicochemical evaluation of kinetics and adsorption models. In *Biohydrometallurgy: A Meeting Point between Microbial Ecology, Metal Recovery Processes and Environmental Remediation*, Donati, E.R., Viera, M.R., Tavani, E.L., Giaveno, M.A., Lavalle, T.L., Chiacchiarini, P.A., Eds. Trans Tech Publications, Switzerland: 2009; Vol. 71-73, pp. 605-608.
26. Vlachou, A.; Symeopoulos, B.D.; Koutinas, A.A. A comparative study of neodymium sorption by yeast cells. *Radiochim. Acta* **2009**, *97*, 437-441, doi:10.1524/ract.2009.1632.
27. Park, H.-J.; Tavlarides, L.L. Adsorption of neodymium(III) from aqueous solutions using a phosphorus functionalized adsorbent. *Ind. Eng. Chem. Res.* **2010**, *49*, 12567-12575, doi:10.1021/ie100403b.
28. Zhang, L.; Wu, D.; Zhu, B.; Yang, Y.; Wang, L. Adsorption and selective separation of neodymium with magnetic alginate microcapsules containing the extractant 2-ethylhexyl phosphonic acid mono-2-ethylhexyl ester. *J. Chem. Eng. Data* **2011**, *56*, 2280-2289, doi:10.1021/je101270j.
29. Zhang, P.; Wang, Y.; Zhang, D.; Bai, H.; Tarasov, V.V. Calixarene-functionalized graphene oxide composites for adsorption of neodymium ions from the aqueous phase. *RSC Adv.* **2016**, *6*, 30384-30394, doi:10.1039/c5ra27509a.
30. Ashour, R.M.; El-Sayed, R.; Abdel-Magied, A.F.; Abdel-Khalek, A.A.; Ali, M.M.; Forsberg, K.; Uheida, A.; Muhammed, M.; Dutta, J. Selective separation of rare earth ions from aqueous solution using functionalized magnetite nanoparticles: kinetic and thermodynamic studies. *Chem. Eng. J.* **2017**, *327*, 286-296, doi:10.1016/j.cej.2017.06.101.
31. Mohamed, W.R.; Metwally, S.S.; Ibrahim, H.A.; El-Sherief, E.A.; Mekhamer, H.S.; Moustafa, I.M.I.; Mabrouk, E.M. Impregnation of task-specific ionic liquid into a

solid support for removal of neodymium and gadolinium ions from aqueous solution. *J. Mol. Liq.* **2017**, *236*, 9-17, doi:10.1016/j.molliq.2017.04.013.
32. Elsalamouny, A.R.; Desouky, O.A.; Mohamed, S.A.; Galhoum, A.A.; Guibal, E. Evaluation of adsorption behavior for U(VI) and Nd(III) ions onto fumarated polystyrene microspheres. *J. Radioanal. Nucl. Chem.* **2017**, *314*, 429-437, doi:10.1007/s10967-017-5389-5.
33. Kucuker, M.A.; Wieczorek, N.; Kuchta, K.; Copty, N.K. Biosorption of neodymium on *Chlorella vulgaris* in aqueous solution obtained from hard disk drive magnets. *PLoS One* **2017**, *12*, Art. N° 0175255, doi:10.1371/journal.pone.0175255.
34. Hisada, M.; Kawase, Y. Recovery of rare-earth metal neodymium from aqueous solutions by poly-gamma-glutamic acid and its sodium salt as biosorbents: Effects of solution pH on neodymium recovery mechanisms. *J. Rare Earths* **2018**, *36*, 528-536, doi:10.1016/j.jre.2018.01.001.
35. Liao, Q.; Zou, D.; Pan, W.; Linghu, W.; Shen, R.; Li, X.; Asiri, A.M.; Alamry, K.A.; Sheng, G.; Zhan, L., et al. Highly efficient capture of Eu(III), La(III), Nd(III), Th(IV) from aqueous solutions using g-C_3N_4 nanosheets. *J. Mol. Liq.* **2018**, *252*, 351-361, doi:10.1016/j.molliq.2017.12.145.
36. Wang, F.; Zhao, J.; Liu, H.; Luo, Y.; Wang, W. Preparation of double carboxylic corn stalk gels and their adsorption properties towards rare earths(III). *Waste Biomass Valorization* **2018**, *9*, 1945-1954, doi:10.1007/s12649-017-9954-5.
37. Hamadneh, I.; Alatawi, A.; Zalloum, R.; Albuqain, R.; Alsotari, S.; Khalili, F.I.; Al-Dujaili, A.H. Comparison of Jordanian and standard diatomaceous earth as an adsorbent for removal of Sm(III) and Nd(III) from aqueous solution. *Environ. Sci. Pollut. Res.* **2019**, *26*, 20969-20980, doi:10.1007/s11356-019-05294-9.
38. Najafi, M.; Chevinli, A.S.; Srivastava, V.; Sillanpaa, M. Augmentation of neodymium ions removal from water using two lanthanides-based MOF: Ameliorated efficiency by synergistic interaction of two lanthanides. *J. Chem. Eng. Data* **2019**, *64*, 3105-3112, doi:10.1021/acs.jced.9b00207.
39. Kautenburger, R.; Beck, H.P. Influence of geochemical parameters on the sorption and desorption behaviour of europium and gadolinium onto kaolinite. *J. Environ. Monit.* **2010**, *12*, 1295-1301, doi:10.1039/b914861b.
40. Radhika, S.; Nagaraju, V.; Nagaphani Kumar, B.; Kantam, M.L.; Reddy, B.R. Solid-liquid extraction of Gd(III) and separation possibilities of rare earths from phosphoric acid solutions using Tulsion CH-93 and Tulsion CH-90 resins. *J. Rare Earths* **2012**, *30*, 1270-1275, doi:https://doi.org/10.1016/S1002-0721(12)60219-1.
41. Hamed, M.M.; Rizk, S.E.; Nayl, A.A. Adsorption kinetics and modeling of gadolinium and cobalt ions sorption by an ion-exchange resin. *Part. Sci. Technol.* **2016**, *34*, 716-724, doi:10.1080/02726351.2015.1112328.
42. Gomes Rodrigues, D.; Monge, S.; Pellet-Rostaing, S.p.; Dacheux, N.; Bouyer, D.; Faur, C. Sorption properties of carbamoylmethylphosphonated-based polymer combining both sorption and thermosensitive properties: New valuable hydrosoluble materials for rare earth elements sorption. *Chem. Eng. J.* **2019**, *355*, 871-880, doi:https://doi.org/10.1016/j.cej.2018.08.190.
43. Kondo, K.; Umetsu, M.; Matsumoto, M. Adsorption characteristics of gadolinium and dysprosium with microcapsules containing an extractant. *J. Water Process Eng.* **2015**, *7*, 237-243, doi:10.1016/j.jwpe.2015.06.006.
44. Wang, F.; Wang, W.; Zhu, Y.; Wang, A. Evaluation of Ce(III) and Gd(III) adsorption from aqueous solution using CTS-g-(AA-co-SS)/ISC hybrid hydrogel adsorbent. *J. Rare Earths* **2017**, *35*, 697-708, doi:10.1016/s1002-0721(17)60966-9.

45. Guo, L.; Xu, Y.; Zhuo, M.; Liu, L.; Xu, Q.; Wang, L.; Shi, C.; Ye, B.; Fan, X.; Chen, W. Highly efficient removal of Gd(III) using hybrid hydrosols of carbon nanotubes/graphene oxide in dialysis bags and synergistic enhancement effect. *Chem. Eng. J.* **2018**, *348*, 535-545, doi:10.1016/j.cej.2018.04.212.
46. Oyewo, O.A.; Onyango, M.S.; Wolkersdorfer, C. Lanthanides removal from mine water using banana peels nanosorbent. *Int. J. Environ. Sci. Technol.* **2018**, *15*, 1265-1274, doi:10.1007/s13762-017-1494-9.
47. Pylypchuk, I.V.; Kolodynska, D.; Gorbyk, P.P. Gd(III) adsorption on the DTPA-functionalized chitosan/magnetite nanocomposites. *Sep. Sci. Technol.* **2018**, *53*, 1006-1016, doi:10.1080/01496395.2017.1330830.
48. Sappidi, P.; Boda, A.; Ali, S.M.; Singh, J.K. Adsorption of gadolinium (Gd^{3+}) ions on the dibenzo crown ether (DBCE) and dicyclo hexano crown ether (DCHCE) grafted on the polystyrene surface: Insights from all atom molecular dynamics simulations and experiments. *J. Phys. Chem. C* **2019**, *123*, 12276-12285, doi:10.1021/acs.jpcc.9b01722.
49. Yin, W.; Liu, L.; Tang, S.; Zhang, H.; Pan, X.; Chi, R. Facile synthesis of triazole and carboxyl-functionalized cellulose-based adsorbent via click chemistry strategy for efficient Gd(III) removal. *Cellulose* **2019**, *26*, 7107-7123, doi:10.1007/s10570-019-02606-7.
50. Zheng, X.; Zhang, Y.; Bian, T.; Zhang, Y.; Zhang, F.; Yan, Y. Selective extraction of gadolinium using free-standing imprinted mesoporous carboxymethyl chitosan films with high capacity. *Cellulose* **2019**, *26*, 1209-1219, doi:10.1007/s10570-018-2124-5.
51. Yin, W.; Liu, L.; Zhang, H.; Tang, S.; Chi, R. A facile solvent-free and one-step route to prepare amino-phosphonic acid functionalized hollow mesoporous silica nanospheres for efficient Gd(III) removal. *J. Cleaner Prod.* **2020**, *243*, Art. N° 118688, doi:10.1016/j.jclepro.2019.118688.
